# Estimating individualized treatment effects from randomized controlled trials: a simulation study to compare risk-based approaches

**DOI:** 10.1186/s12874-023-01889-6

**Published:** 2023-03-28

**Authors:** Alexandros Rekkas, Peter R. Rijnbeek, David M. Kent, Ewout W. Steyerberg, David van Klaveren

**Affiliations:** 1 Department of Medical Informatics, Erasmus Medical Center, P.O. Box 2040, 3000 CA Rotterdam, The Netherlands; 2 Predictive Analytics and Comparative Effectiveness Center, Institute for Clinical Research and Health Policy Studies, Tufts Medical Center, Boston, MA USA; 3 Department of Biomedical Data Sciences, Leiden University Medical Center, Leiden, The Netherlands; 4 Department of Public Health, Erasmus Medical Center, Rotterdam, The Netherlands

**Keywords:** Treatment effect heterogeneity, Absolute benefit, Prediction models

## Abstract

**Background:**

Baseline outcome risk can be an important determinant of absolute treatment benefit and has been used in guidelines for “personalizing” medical decisions. We compared easily applicable risk-based methods for optimal prediction of individualized treatment effects.

**Methods:**

We simulated RCT data using diverse assumptions for the average treatment effect, a baseline prognostic index of risk, the shape of its interaction with treatment (none, linear, quadratic or non-monotonic), and the magnitude of treatment-related harms (none or constant independent of the prognostic index). We predicted absolute benefit using: models with a constant relative treatment effect; stratification in quarters of the prognostic index; models including a linear interaction of treatment with the prognostic index; models including an interaction of treatment with a restricted cubic spline transformation of the prognostic index; an adaptive approach using Akaike’s Information Criterion. We evaluated predictive performance using root mean squared error and measures of discrimination and calibration for benefit.

**Results:**

The linear-interaction model displayed optimal or close-to-optimal performance across many simulation scenarios with moderate sample size (*N* = 4,250; ~ 785 events). The restricted cubic splines model was optimal for strong non-linear deviations from a constant treatment effect, particularly when sample size was larger (*N* = 17,000). The adaptive approach also required larger sample sizes. These findings were illustrated in the GUSTO-I trial.

**Conclusions:**

An interaction between baseline risk and treatment assignment should be considered to improve treatment effect predictions.

**Supplementary Information:**

The online version contains supplementary material available at 10.1186/s12874-023-01889-6.

## Introduction

Predictive approaches to heterogeneity of treatment effects (HTE) aim at the development of models predicting either individualized effects or which of two (or more) treatments is better for an individual with regard to a specific outcome of interest [1]. These predictive approaches include both regression and machine learning techniques and are the subject of active research [2–5]. In prior work, we divided regression-based methods for the evaluation of treatment effect heterogeneity in three broader categories: risk modeling, treatment effect modeling and optimal treatment regime methods [6]. Risk modeling methods use only prognostic factors to define patient subgroups, relying on the mathematical dependency between baseline risk and treatment effect [2, 7]. Treatment effect modeling methods use both prognostic factors and treatment effect modifiers to explore characteristics that interact with the effects of therapy. They can be applied in one stage by directly modeling treatment-covariate interactions, in which case penalization of the interaction effects is needed to reduce the effects of overfitting [8], or in two stages that rely on updating working absolute benefit models [9, 10]. Optimal treatment regime methods focus primarily on treatment effect modifiers in order to classify the trial population into those who benefit from treatment and those who do not [11–14].

In a previous simulation study, modeling treatment-covariate interactions often led to poorly calibrated predictions of benefit on the absolute scale (risk difference between treatment arms), compared to risk-modeling methods [15]. In the presence of true treatment-covariate interactions, however, effect modeling methods were better able to separate lower from higher benefit patients [15, 16]. By assuming treatment effect is a function of baseline risk, risk modeling methods impose a restriction on the shape of treatment effect heterogeneity. With smaller sample sizes or limited information on effect modification, risk modeling methods, because of their reduced complexity, can provide a good option for evaluating treatment effect heterogeneity. Conversely, with larger sample sizes and/or a limited set of well-studied strong effect modifiers, treatment effect modeling methods can potentially result in a better bias-variance tradeoff. Therefore, the setting in which treatment effect heterogeneity is evaluated is crucial for the selection of the optimal approach.

Risk modeling methods predict similar treatment benefit for patients with similar baseline outcome risk, i.e. a similar probability of experiencing the outcome of interest in the absence of treatment. These methods are not new and are quite intuitive to practitioners [6]. Often medical guidelines rely on a risk stratified approach to target treatments to different patients. In addition, re-analyses of studies that only looked at overall results using risk stratification often resulted to important insight on how treatment effects varied for different patients. For example, a risk stratified analysis of patients with acute myocardial infarction (MI) based on the Thrombolysis in Myocardial Infarction (TIMI) risk score found no benefit for patients who underwent primary angioplasty compared to fibrinolysis. However, there was a significant benefit for patients with a high TIMI score [17]. Infants at lower risk of bronchopulmonary dysplasia benefit relatively more from vitamin A therapy than infants at higher risk [18]. Finally, higher risk prediabetic patients benefit relatively more from metformin than lower risk patients [19].

Most often, risk-modeling approaches are carried out in two steps: first a risk prediction model is developed externally or internally on the entire RCT population, “blinded” to treatment; then the RCT population is stratified using this prediction model to evaluate risk-based treatment effect variation [7, 20, 21]. This approach identified substantial absolute treatment effect differences between low-risk and high-risk patients in a re-analysis of 32 large trials [22]. However, even though treatment effect estimates at the risk subgroup level may be accurate, these estimates may not apply to individual patients, as homogeneity of treatment effects is assumed within risk strata. With stronger overall treatment effect and larger variability in predicted risks, patients assigned to the same risk subgroup may still differ substantially with regard to their benefits from treatment.

In the current simulation study, we aim to summarize and compare different risk-based models for predicting treatment effects. We simulate different relations between baseline risk and treatment effects and also consider potential harms of treatment. We illustrate the different models by a case study of predicting individualized effects of treatment for acute myocardial infarction in a large RCT.

## Methods

### Notation

We observe RCT data $\left(Z,X,Y\right)$, where for each patient ${Z}_{i}=0,1$ is the treatment status, ${Y}_{i}=0,1$ is the observed outcome and ${X}_{i}$ is a set of measured covariates. Let $\{{Y}_{i}\left(z\right),z=0,1\}$ denote the unobservable potential outcomes. We observe ${Y}_{i}={Z}_{i}{Y}_{i}\left(1\right)+\left(1-{Z}_{i}\right){Y}_{i}\left(0\right)$. We are interested in predicting the conditional average treatment effect (CATE),

$$\tau \left(x\right)=E\{Y\left(0\right)-Y\left(1\right)|X=x\}$$

Assuming that $\left(Y\left(0\right),Y\left(1\right)\right)\perp Z|X$, as we are in the RCT setting, we can predict CATE from

$$\begin{array}{ll}\tau\left(x\right)&=E\{Y\left(0\right)\hspace{0.25em}\vert\hspace{0.25em}X=x\}-E\{Y\left(1\right)\hspace{0.25em}\vert\hspace{0.25em}X=x\}\\&=E\{Y\hspace{0.25em}\vert\hspace{0.25em}X=x,Z=0\}-E\{Y\hspace{0.25em}\vert\hspace{0.25em}X=x,Z=1\}\end{array}$$

### Simulation scenarios

We simulated a typical RCT, comparing equally-sized treatment and control arms in terms of a binary outcome. For each patient we generated 8 baseline covariates ${X}_{1},\dots ,{X}_{4}\sim N\left(0,1\right)$ and ${X}_{5},\dots ,{X}_{8}\sim B\left(1,0.2\right)$. Outcomes in the control arm were generated from Bernoulli variables with true probabilities following a logistic regression model including all baseline covariates, i.e. $P\left(Y\left(0\right)=1 | X=x\right)={\text{expit}}\left(l{p}_{0}\right)={e}^{l{p}_{0}}/\left(1+{e}^{l{p}_{0}}\right)$, with $l{p}_{0}=l{p}_{0}\left(x\right)={x}^{t}\beta$. In the base scenarios coefficient values $\beta$ were such, that the control event rate was 20% and the discriminative ability of the true prediction model measured using Harrell’s c-statistic was 0.75. The c-statistic represents the probability that for a randomly selected discordant pair from the sample (patients with different outcomes) the prediction model assigns larger risk to the patient with the worse outcome. For the simulations this was achieved by selecting $\beta$ values such that the true prediction model would achieve a c-statistic of 0.75 in a simulated control arm with 500,000 patients. We achieved a true c-statistic of 0.75 by setting $\beta ={\left(-2.08,0.49,\dots ,0.49\right)}^{t}$.

Outcomes in the treatment arm were first generated using 3 simple scenarios for a true constant odds ratio (OR): absent (OR = 1), moderate (OR = 0.8) or strong (OR = 0.5) constant relative treatment effect. We then introduced linear, quadratic and non-monotonic deviations from constant treatment effects using:

$$l{p}_{1}={\gamma }_{0}+{\gamma }_{1}\left(l{p}_{0}-c\right)+{\gamma }_{2}{\left(l{p}_{0}-c\right)}^{2},$$

where $l{p}_{1}$ is the true linear predictor in the treatment arm, so that $P\left(Y\left(1\right)=1 | X=x\right)={\text{expit}}\left(l{p}_{1}\right)$, $\gamma ={\left({\gamma }_{0},{\gamma }_{1},{\gamma }_{2}\right)}^{t}$ controls the shape of the evolution of treatment effect as a function of baseline risk (type and strength of deviations from the constant treatment effect setting), while $c$ allows us to shift the proposed shape function to achieve the desired overall event rates. For example, to simulate a constant treatment effect with ${\text{OR}}=0.8$ we would set $\gamma ={\left(\mathrm{log}\left(0.8\right),1,0\right)}^{t}$ and $c=0$. Finally, we incorporated constant absolute harms for all treated patients, such that $P\left(Y\left(1\right)=1 | X=x\right)={\text{expit}}\left(l{p}_{1}\right)+{\text{harm}}$. The sample size for the base scenarios was set to 4,250 (80% power to find a statistically significant treatment effect at the 5% significance level, when the true treatment effect is an odds ratio of 0.8). We evaluated the impact of smaller or larger sample sizes of 1,063 and 17,000, respectively. We also evaluated the impact of risk model discriminative ability, adjusting the baseline covariate coefficients, such that the c-statistic of the regression model in the control arm was 0.65 and 0.85, respectively. These settings resulted in a simulation study of 648 scenarios covering the HTE observed in 32 large trials as well as many other potential variations of risk-based treatment effect (Supplement, Sects. 2 and 3) [22]. We analyzed the sensitivity of the results to correlation between baseline characteristics. We first sampled 8 continuous variables ${W}_{1},\dots ,{W}_{8}\sim N\left(0,\Sigma \right)$. We then generated four continuous baseline covariates from ${X}_{1}={W}_{1},\dots ,{X}_{4}={W}_{4}$ and four binary covariates with 20% prevalence from ${X}_{5}=I\left({W}_{5}>{z}_{0.8}\right),\dots , {X}_{8}=I({W}_{8}>{z}_{0.8})$, where $I$ is the indicator function and $P\left(U\le 0.8\right)={z}_{0.8}$ for random variable $U\sim N(\mathrm{0,1})$. The covariance matrix $\Sigma$ was such that $cor\left({X}_{i},{X}_{j}\right)=0.5$ for any $i\ne j$. To ensure that the outcome rate in the untreated subset was 20% and that true prediction c-statistic remained equal to the nominal values of the main simulation analyses, we adjusted the coefficients of the true outcome model. More details on the sensitivity analyses can be found in the Supplement, Sect. 9.

### Individualized risk-based benefit predictions

In each simulation run, we internally developed a prediction model on the entire population, using a logistic regression model with main effects for all baseline covariates and treatment assignment. Individual risk predictions were derived by setting treatment assignment to 0*.* A more intuitive approach would be to derive the prediction model solely on the control patients. However, this has been shown to lead to biased benefit predictions, because with limited sample size the model will be overfitted to the control arm and induce spurious treatment interactions [15, 23, 24].

We compared different methods for predicting absolute treatment benefit, that is the risk difference between distinct treatment assignments. We use the term absolute treatment benefit to distinguish from relative treatment benefit that relies on the ratio of predicted risk under different treatment assignments.

A *stratified HTE method* has been suggested as an alternative to traditional subgroup analyses [20, 21]. Patients are stratified into equally-sized risk strata—in this case based on risk quartiles. Absolute treatment effects, within risk strata, expressed as absolute risk differences, are estimated by the difference in event rate between control and treatment arm patients. We considered this approach as a reference, expecting it to perform worse than the other candidates, as its objective is to provide an illustration of HTE rather than to optimize individualized benefit predictions.

Second, we fitted a logistic regression model which assumes *constant relative treatment effect* (constant odds ratio), that is, $P\left(Y=1 | X=x,Z=z;\widehat{\beta }\right)={\text{expit}}\left({\widehat{lp}}_{0}+{\delta }_{1}z\right)$. Hence, absolute benefit is predicted from $\tau \left(x;\widehat{\beta }\right)={\text{expit}}\left({\widehat{lp}}_{0}\right)-{\text{expit}}\left({\widehat{lp}}_{0}+{\delta }_{1}\right)$, where ${\delta }_{1}$ is the log of the assumed constant odds ratio and ${\widehat{lp}}_{0}={\widehat{lp}}_{0}\left(x;\widehat{\beta }\right)={x}^{t}\widehat{\beta }$ the linear predictor of the estimated baseline risk model.

Third, we fitted a logistic regression model including treatment, the risk linear predictor, and their linear interaction, that is, $P\left(Y=1 | X=x,Z=z;\widehat{\beta }\right)={\text{expit}}\left({\delta }_{0}+{\delta }_{1}z+{\delta }_{2}{\widehat{lp}}_{0}+{\delta }_{3}z{\widehat{lp}}_{0}\right)$. Absolute benefit is then estimated from $\tau \left(x;\widehat{\beta }\right)={\text{expit}}\left({\delta }_{0}+{\delta }_{2}{\widehat{lp}}_{0}\right)-{\text{expit}}\left({(\delta }_{0}+{\delta }_{1})+{(\delta }_{2}{+{\delta }_{3})\widehat{lp}}_{0}\right)$. We will refer to this method as the *linear interaction* approach.

Fourth, we used *restricted cubic splines* (RCS) to relax the linearity assumption on the effect of the linear predictor [25]. We considered splines with 3 (RCS-3), 4 (RCS-4) and 5 (RCS-5) knots, together with their interaction with treatment, to compare models with different levels of flexibility (Supplement, Sect. 4).

Finally, we considered an adaptive approach using Akaike’s Information Criterion (AIC) for model selection. More specifically, we ranked the constant relative treatment effect model, the linear interaction model, and the RCS models with 3, 4, and 5 knots based on their AIC and selected the one with the lowest value. The extra degrees of freedom were 1 (linear interaction), 2, 3 and 4 (RCS models) for these increasingly complex interactions with the treatment effect.

### Evaluation metrics

We evaluated the predictive accuracy of the considered methods by the root mean squared error (RMSE):

$${\text{RMSE}}=\sqrt{\frac{1}{n}\sum_{i=1}^{n}(\tau \left({\mathbf{x}}_{i}\right)-\widehat{\tau }\left({\mathbf{x}}_{i}\right){)}^{2}}$$

We compared the discriminative ability of the methods under study using c-for-benefit and the integrated calibration index (ICI) for benefit (Supplement, Sect. 6). Since true patient-specific benefit is unobservable, we calculated observed benefit using the following approach: patients in each treatment arm are ranked based on their predicted benefit and then matched 1:1 on predicted benefit across treatment arms. Observed treatment benefit is defined as the difference of observed outcomes between the untreated and the treated patient of each matched patient pair. Since matching may not be perfect, that is, predicted benefits for the patients of the pair may not be equal, pair-specific predicted benefit is defined as the average of predicted benefit within each matched patient pair [26]. Then, the c-for-benefit represents the probability that from two randomly chosen predicted benefit-matched patient pairs with unequal observed benefit, the pair with greater observed benefit also has a higher predicted benefit. We evaluated calibration in a similar manner, using the integrated calibration index (ICI) for benefit [27]. The observed benefits are regressed on the predicted benefits using a locally weighted scatterplot smoother (loess). The ICI-for-benefit is the average absolute difference between predicted and smooth observed benefit. Values closer to 0 represent better calibration. For each scenario we performed 500 replications, within which all the considered models were fitted. We simulated a super-population of size 500,000 for each scenario within which we calculated RMSE and discrimination and calibration for benefit of all the models in each replication.

### Empirical illustration

We demonstrated the different methods using 30,510 patients with acute myocardial infarction (MI) included in the GUSTO-I trial. 10,348 patients were randomized to tissue plasminogen activator (tPA) treatment and 20,162 were randomized to streptokinase. The outcome of interest was 30-day mortality (total of 2,128 events), recorded for all patients.

This dataset has been used extensively in prior studies [28, 29]. Therefore, we used the same set of seven covariates that was previously used to fit a logistic regression model (age, Killip class, systolic blood pressure, heart rate, an indicator of previous MI, and the location of MI) along with a binary covariate for treatment indication, to predict 30-day mortality risk (Supplement, Sect. 10). Predicted baseline risk is derived by setting the treatment indicator to 0 for all patients.

## Results

### Simulations

**Fig. 1:**
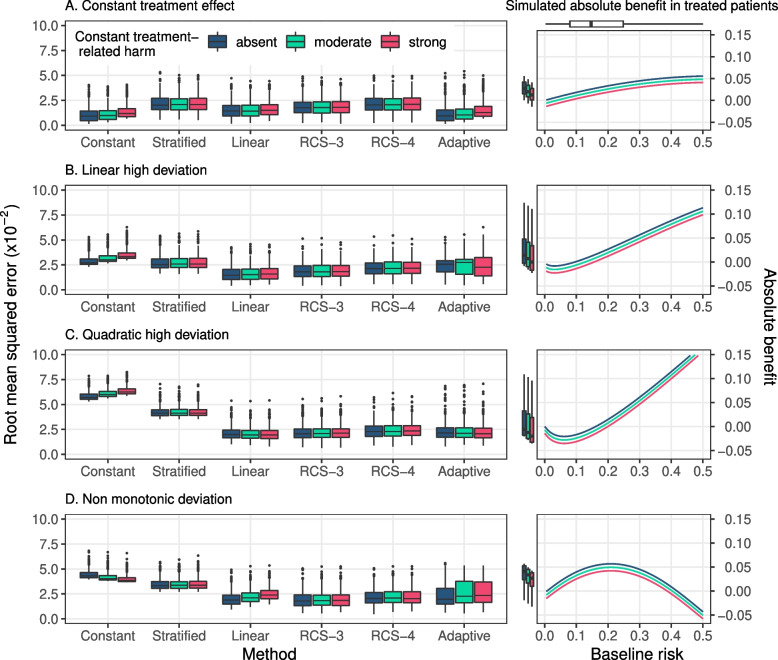
RMSE of the considered methods across 500 replications was calculated from a simulated super-population of size 500,000. The scenario with true constant relative treatment effect (panel **A**) had a true prediction c-statistic of 0.75 and sample size of 4250. The RMSE is also presented for strong linear (panel **B**), strong quadratic (panel **C**), and non-monotonic (panel **D**) deviations from constant relative treatment effects. Panels on the right side present the true relations between baseline risk (x-axis) and absolute treatment benefit (y-axis). The 2.5, 25, 50, 75, and 97.5 percentiles of the risk distribution are expressed by the boxplot on the top. The 2.5, 25, 50, 75, and 97.5 percentiles of the true benefit distributions are expressed by the boxplots on the side of the right-handside panel

The constant treatment effect approach outperformed other approaches in the base case scenario (*N* = 4,250; OR = 0.8; c-statistic = 0.75; no absolute treatment harm) with a true constant treatment effect (median RMSE: constant treatment effect 0.009; linear interaction 0.014; RCS-3 0.018). The linear interaction model was optimal under true linear deviations (median RMSE: constant treatment effect 0.027; linear interaction 0.015; RCS-3 0.018; Fig. 1 panels A-C) and even in the presence of true quadratic deviations (median RMSE: constant treatment effect 0.057; linear interaction 0.020; RCS-3 0.021; Fig. 1 panels A-C) from a constant relative treatment effect. With non-monotonic deviations, RCS-3 slightly outperformed the linear interaction model (median RMSE: linear interaction 0.019; RCS-3 0.018; Fig. 1 panel D). With strong treatment-related harms the results were very similar in most scenarios (Fig. 1 panels A-C). Under non-monotonic deviations the optimal performance of RCS-3 was more pronounced (median RMSE: linear interaction 0.024; RCS-3 0.019; Fig. 1 panel D). A stronger average treatment effect (OR = 0.5) resulted in higher variability of the true treatment effects on the absolute scale (difference in true outcome probabilities between treatment arms) and consequently to larger RMSE for all approaches. When we assumed a stronger relative treatment effect, the relative differences between approaches were similar to the base-case scenario (Supplement, Figure S10).

The adaptive approach had limited loss of performance in terms of the median RMSE to the best-performing method in each scenario. However, compared to the best-performing approach, its RMSE was more variable in scenarios with linear and non-monotonic deviations, especially when also including moderate or strong treatment-related harms. On closer inspection, we found that this behavior was caused by selecting the constant treatment effect model in a substantial proportion of the replications (Supplement, Figure S3).

**Fig. 2:**
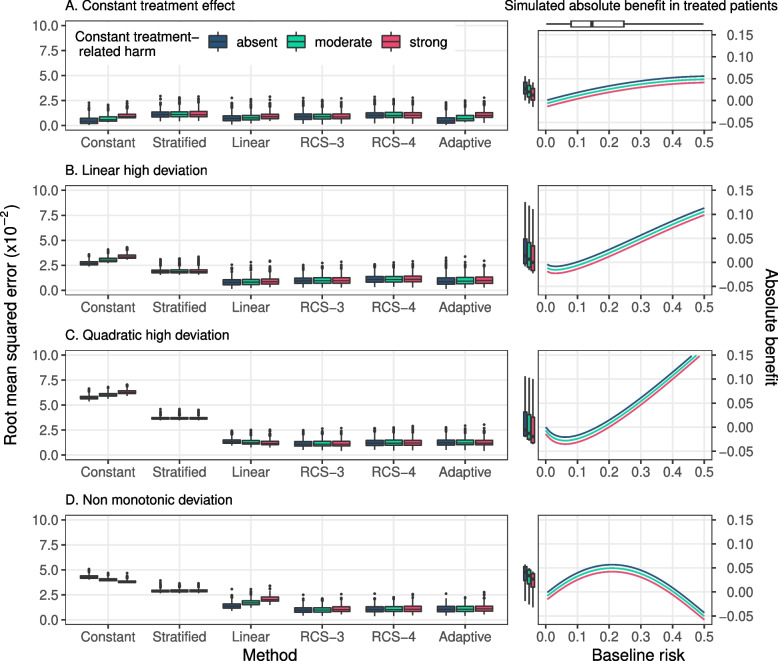
RMSE of the considered methods across 500 replications calculated in simulated samples of size 17,000 rather than 4,250 in Fig. 1. RMSE was calculated on a super-population of size 500,000

Increasing the sample size to 17,000 favored RCS-3 the most (Fig. 2). The difference in performance with the linear interaction approach was more limited in settings with a constant treatment effect (median RMSE: linear interaction 0.007; RCS-3 0.009) and with a true linear interaction (median RMSE: linear interaction 0.008; RCS-3 0.009) and more emphasized in settings with strong quadratic deviations (median RMSE: linear interaction 0.013; RCS-3 0.011) and non-monotonic deviations (median RMSE: linear interaction 0.014; RCS-3 0.010). Due to the large sample size, the RMSE of the adaptive approach was even more similar to the best-performing method, and the constant relative treatment effect model was less often wrongly selected (Supplement, Figure S4).

**Fig. 3:**
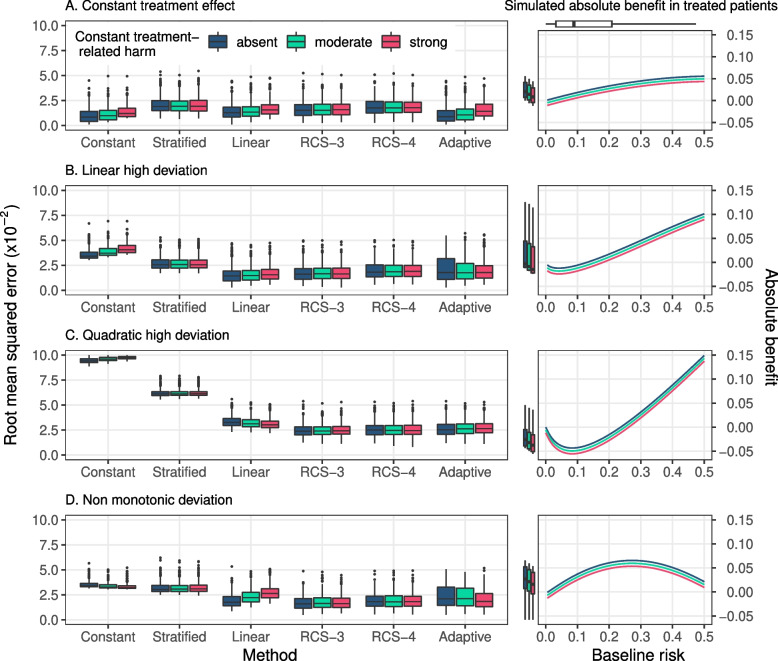
RMSE of the considered methods across 500 replications calculated in simulated samples 4,250. True prediction c-statistic of 0.85. RMSE was calculated on a super-population of size 500,000

Similarly, when we increased the c-statistic of the true prediction model to 0.85 (OR = 0.8 and *N* = 4,250), RCS-3 had the lowest RMSE in the case of strong quadratic or non-monotonic deviations and very comparable performance to the – optimal – linear interaction model in the case of strong linear deviations (median RMSE of 0.016 for RCS-3 compared to 0.014 for the linear interaction model; Fig. 3). Similar to the base case scenario the adaptive approach wrongly selected the constant treatment effect model (23% and 25% of the replications in the strong linear and non-monotonic deviation scenarios without treatment-related harms, respectively), leading to increased variability of the RMSE (Supplement, Figure S5).

**Fig. 4:**
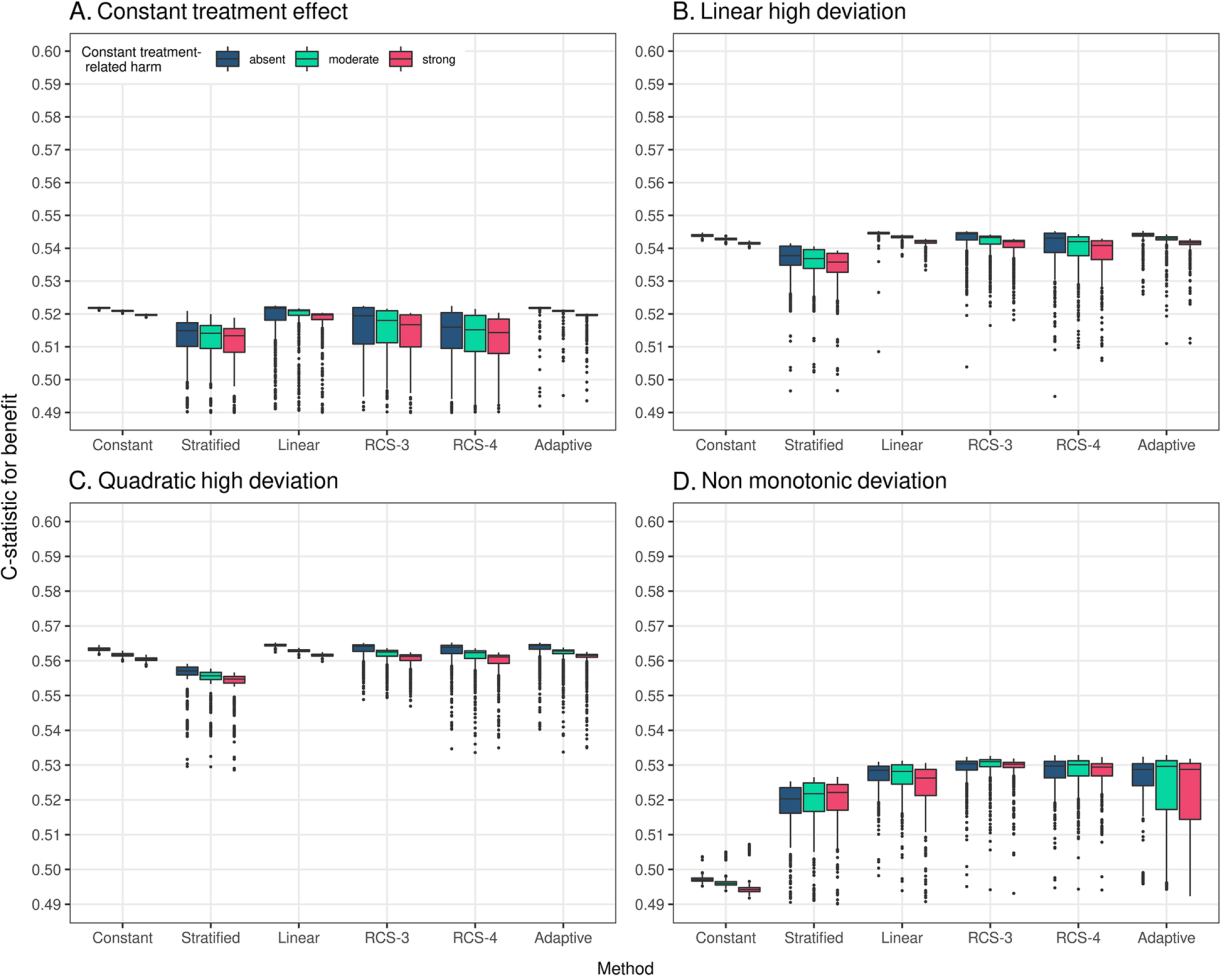
Discrimination for benefit of the considered methods across 500 replications calculated in simulated samples of size 4,250 using the c-statistic for benefit. The c-statistic for benefit represents the probability that from two randomly chosen matched patient pairs with unequal observed benefit, the pair with greater observed benefit also has a higher predicted benefit. True prediction c-statistic of 0.75

With a true constant relative treatment effect, discrimination for benefit was only slightly lower for the linear interaction model, but substantially lower for the non-linear RCS approaches (Fig. 4; panel A). With strong linear or quadratic deviations from a constant relative treatment effect, all methods discriminated quite similarly (Fig. 4 panels B-C). With non-monotonic deviations, the constant effect model had much lower discriminative ability compared to all other methods (median c-for-benefit of 0.500 for the constant effects model, 0.528 for the linear interaction model and 0.530 Fig. 4; panel D). The adaptive approach was unstable in terms of discrimination for benefit, especially with treatment-related harms. With increasing number of RCS knots, we observed decreasing median values and increasing variability of the c-for-benefit in all scenarios. When we increased the sample size to 17,000 we observed similar trends, however the performance of all methods was more stable (Supplement, Figure S6). Finally, when we increased the true prediction c-statistic to 0.85 the adaptive approach was, again, more conservative, especially with non-monotonic deviations and null or moderate treatment-related harms (Supplement, Figure S7).

**Fig. 5:**
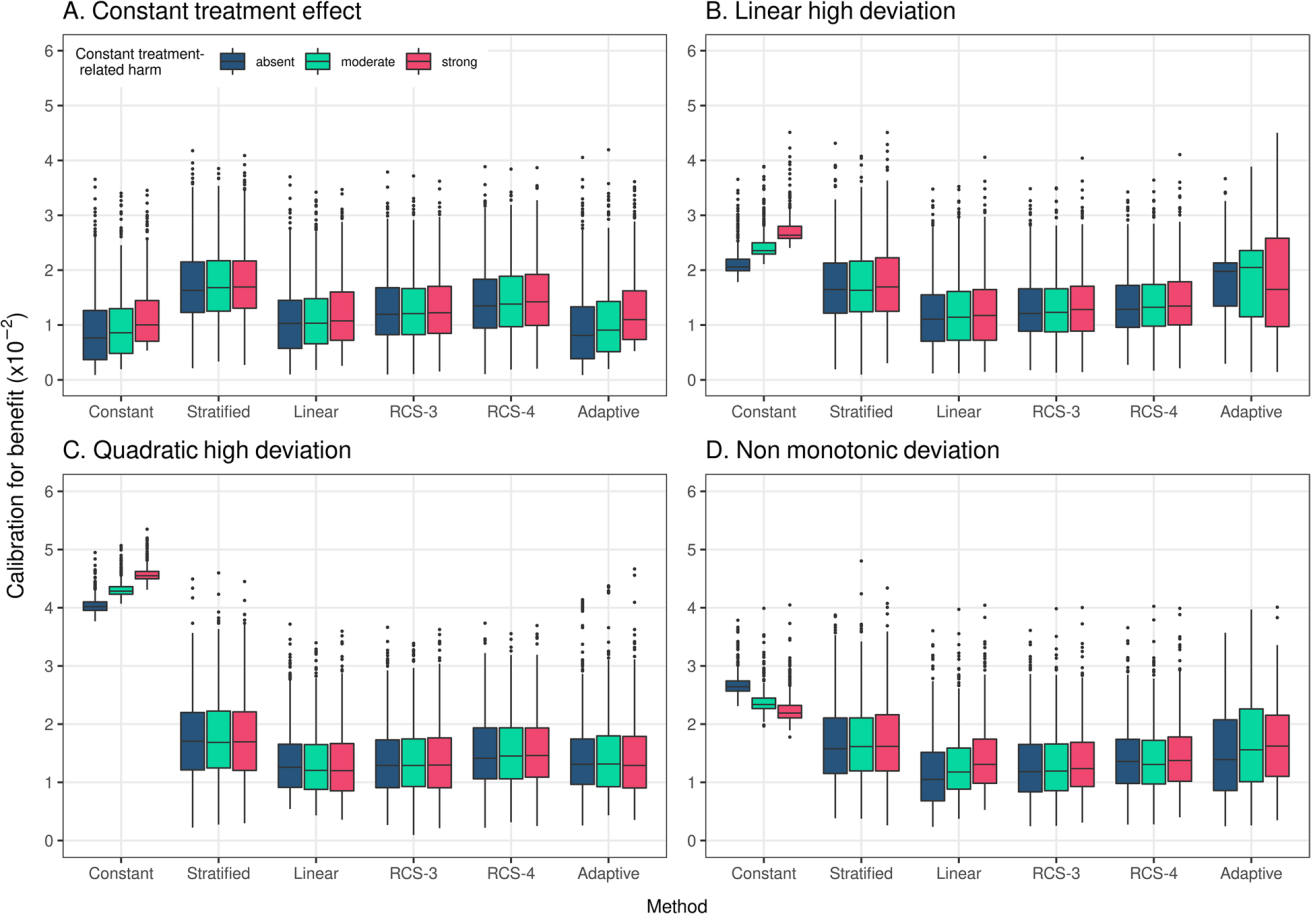
Calibration for benefit of the considered methods across 500 replications calculated in a simulated sample of size 500,000. True prediction c-statistic of 0.75 and sample size of 4,250

In terms of calibration for benefit, the constant effects model outperformed all other models in the scenario with true constant treatment effects, but was miscalibrated for all deviation scenarios (Fig. 5). The linear interaction model showed best or close to best calibration across all scenarios and was only outperformed by RCS-3 in the case of non-monotonic deviations and treatment-related harms (Fig. 5 panel D). The adaptive approach was worse calibrated under strong linear and non-monotonic deviations compared to the linear interaction model and RCS-3. When we increased the sample size to 17,000 (Supplement, Figure S8) or the true prediction c-statistic to 0.85 (Supplement, Figure S9), RCS-3 was somewhat better calibrated than the linear interaction model with strong quadratic deviations.

Our main conclusions remained unchanged in the sensitivity analyses where correlations between baseline characteristics were introduced (Supplement, Figures S16, S17, and S18).

The results from all individual scenarios can be explored online at https://mi-erasmusmc.shinyapps.io/HteSimulationRCT/. Additionally, all the code for the simulations can be found at https://github.com/mi-erasmusmc/HteSimulationRCT

### Empirical illustration

We used the derived prognostic index to fit a constant treatment effect, a linear interaction and an RCS-3 model individualizing absolute benefit predictions. Following our simulation results, RCS-4 and RCS-5 models were excluded. Finally, an adaptive approach with the 3 candidate models was applied.

**Fig. 6:**
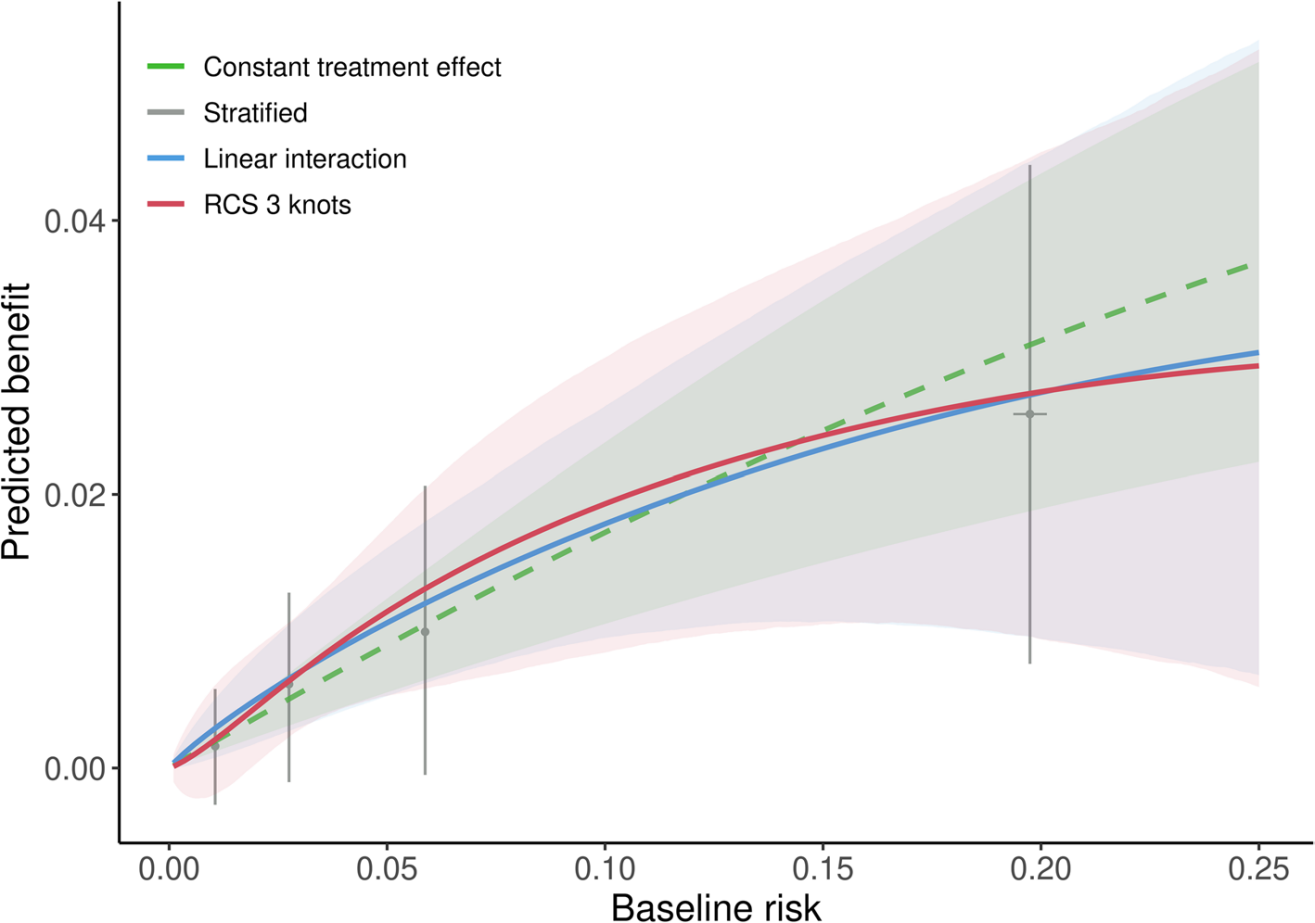
Individualized absolute benefit predictions based on baseline risk when using a constant treatment effect approach, a linear interaction approach and RCS smoothing using 3 knots. Risk stratified estimates of absolute benefit are presented within quartiles of baseline risk as reference. 95% confidence bands were generated using 10,000 bootstrap resamples, where the prediction model was refitted in each run to capture the uncertainty in baseline risk predictions. For the risk stratification approach, we also provide 95% confidence intervals for the baseline risk quarter-specific average predicted risk over the 10,000 bootstrap samples

Predicted absolute benefit was derived as the difference of predicted acute MI risk between treatment arms, if all other predictors remained unchanged. All considered methods provided similar fits, predicting increasing absolute benefits for patients with higher baseline risk predictions, and followed the evolution of the stratified estimates closely (Fig. 6). The constant treatment effect model had somewhat lower AIC compared to the linear interaction model (AIC: versus 9,342), equal cross-validated discrimination (c-for-benefit: 0.525), and slightly better cross-validated calibration (ICI-for benefit: 0.010 versus 0.012). In conclusion, although the sample size (30,510 patients; 2,128 events) allowed for flexible modeling approaches, a simpler constant treatment effect model is adequate for predicting absolute 30-day mortality benefits of treatment with tPA in patients with acute MI.

## Discussion

The linear interaction and the RCS-3 models displayed very good performance under many of the considered simulation scenarios. The linear interaction model was optimal in cases with moderate sample sizes (4.250 patients; ~ 785 events) and moderately performing baseline risk prediction models, that is, it had lower RMSE, was better calibrated for benefit and had better discrimination for benefit, even in scenarios with strong quadratic deviations. In scenarios with true non-monotonic deviations, the linear interaction model was outperformed by RCS-3, especially in the presence of treatment-related harms. Increasing the sample size or the prediction model’s discriminative ability favored RCS-3, especially in scenarios with strong non-linear deviations from a constant treatment effect.

Our simulation results clearly express the trade-off between the advantages of flexibly modeling the relationship between baseline risk and treatment effect and the disadvantages of overfitting this relationship to the sample at hand. With infinite sample size, the more flexible approach (here RCS) will be optimal, but in practice, with limited sample size, parsimonious models may be preferable. Even with the substantial sample size of our base case scenario, the (less flexible) linear interaction model performed better than the (more flexible) RCS approach for most simulation settings. The even less flexible constant treatment effect model, however, was only optimal when the treatment effect was truly constant. Moreover, the assumption of a constant treatment effect may often be too strong [22, 30].

RCS-4 and RCS-5 were too flexible in all considered scenarios, as indicated by higher RMSE, increased variability of discrimination for benefit and worse calibration of benefit predictions. Even with larger sample sizes and strong quadratic or non-monotonic deviations, these more flexible methods did not outperform the simpler RCS-3 approach. Higher flexibility may only be helpful under more extreme patterns of HTE compared to the quadratic deviations considered here. Considering interactions in RCS-3 models as the most complex approach often may be reasonable.

Our results can also be interpreted in terms of bias-variance trade-off. The increasingly complex models considered allow for more degrees of freedom which, in turn, increase the variance of our absolute benefit estimates. However, as was clear in our simulations, this increased complexity did not always result in substantial decrease in bias, especially with lower sample sizes and weaker treatment effects. Consequently, in most scenarios the simpler linear interaction model achieved the best bias-variance balance and outperformed the more complex RCS methods, even in the presence of non-linearity in the true underlying relationship between baseline risk and treatment effect. Conversely, the simpler constant treatment effect model was often heavily biased and, despite its lower variance, was outperformed by the other methods in the majority of the considered scenarios.

Increasing the discriminative ability of the risk model reduced RMSE for all methods. Higher discrimination translates in higher variability of predicted risks, which, in turn, allows the considered methods to better capture absolute treatment benefits. As a consequence, better risk discrimination also led to higher discrimination between those with low or high benefit (as reflected in values of c-for-benefit).

The adaptive approach had adequate median performance, following the “true” model in most scenarios. With smaller sample sizes it tended to miss the treatment-baseline risk interaction and selected simpler models (Supplement Sect. 4). This conservative behavior resulted in increased RMSE variability in these scenarios, especially with true strong linear or non-monotonic deviations. Therefore, with smaller sample sizes the simpler linear interaction model may be a safer choice for predicting absolute benefits, especially in the presence of any suspected treatment-related harms.

A limitation of our simulation study is that we assumed treatment benefit to be a function of baseline risk in the majority of the simulation scenarios, thus ignoring any actual treatment effect modification of individual factors. We attempted to expand our scenarios by considering moderate and strong constant treatment-related harms, applied on the absolute scale, in line with previous work [31]. In a limited set of scenarios with true interactions between treatment assignment and covariates, our conclusions remained unchanged (Supplement, Sect. 8). Even though the average error rates increased for all the considered methods, due to the miss-specification of the outcome model, the linear interaction model had the lowest error rates. RCS-3 had very comparable performance. The constant treatment effect model was often biased, especially with moderate or strong treatment-related harms. Future simulation studies could explore the effect of more extensive deviations from risk-based treatment effects.

We only focused on risk-based methods, using baseline risk as a reference in a two-stage approach to individualizing benefit predictions. However, there is a plethora of different methods, ranging from treatment effect modeling to tree-based approaches available in more recent literature [4, 5, 8, 32–36]. Many of these methods rely on incorporating treatment-covariate interactions when predicting benefits. An important caveat of such approaches is their sensitivity to overfitting, which may exaggerate the magnitude of predicted benefits. This can be mitigated using methods such as cross-validation or regularization to penalize the effect of treatment-covariate interactions. In the presence of a limited set of true strong treatment-covariate interactions and adequate sample size, treatment effect modeling methods may outperform risk modeling methods. However, often treatment effect modifiers are unknown and the available sample size does not allow for the exploration of a large number of interaction effects. In these cases, risk modeling approaches like the ones presented here can provide individualized benefit predictions that improve on the “one-size-fits-all” overall RCT result. In a previous simulation study, a simpler risk modeling approach was consistently better calibrated for benefit compared to more complex treatment effect modelling approaches [15]. Similarly, when SYNTAX score II, a model developed for identifying patients with complex coronary artery disease that benefit more from percutaneous coronary intervention or from coronary artery bypass grafting was redeveloped using fewer treatment-covariate interactions had better external performance compared to its predecessor [37, 38].

Finally, in all our simulation scenarios we assumed all covariates to be statistically independent, the effect of continuous covariates to be linear, and no interaction effects between covariates to be present. This can be viewed as a limitation of our extensive simulation study. However, as all our methods are based on the same fitted risk model, we do not expect these assumptions to significantly influence their relative performance.

In conclusion, the linear interaction approach is a viable option with moderate sample sizes and/or moderately performing risk prediction models, assuming a non-constant relative treatment effect plausible. RCS-3 is a better option with more abundant sample size and when non-monotonic deviations from a constant relative treatment effect and/or substantial treatment-related harms are anticipated. Increasing the complexity of the RCS models by increasing the number of knots does not improve benefit prediction. Using AIC for model selection is attractive with larger sample size.

## Supplementary Information

**Additional file 1:** **Supplement Figure S1-S18 and Table S1-S4.**

## Data Availability

The dataset supporting the conclusions of this article is available in the Vanderbilt University repository maintained by the Biostatistics Department, https://hbiostat.org/data/gusto.rda.

## Abbreviations

RCT: Randomized Controlled Trial
HTE: Heterogeneity of Treatment Effect
MI: Myocardial Infarction
CATE: Conditional Average Treatment Effect
OR: Odds Ratio
RCS: Restricted Cubic Splines
AIC: Akaike’s Information Criterion
RMSE: Root Mean Squared Error
ICI: Integrated Calibration Index
loess: Locally Estimated Scatterplot Smoother
tPA: Tissue Plasminogen Activator
GUSTO: Global Utilization of Streptokinase and Tissue plasminogen activator for Occluded coronary arteries
SYNTAX: Synergy Between PCI With Taxus and Cardiac Surgery
