# Supplementary material for: Estimating individualized treatment effects from randomized controlled trials: a simulation study to compare risk-based approaches
Source: BMC Med Res Methodol. 2023 Mar 28;23:74. doi: 10.1186/s12874-023-01889-6 (PMC10045909; doi:10.1186/s12874-023-01889-6)
Supplement: Supplementary file 1 — Additional file 1: Supplement Figure S1-S18 and Table S1-S4. [file 12874_2023_1889_MOESM1_ESM.pdf]

Supplementary material

Contents

1 Notation **2**

2 Simulation settings **2**

2.1 Base-case scenario 3

2.2 Deviations from base-case 3

3 Plausible scenario settings **19**

4 Approaches to individualize benefit predictions **19**

4.1 Risk modeling 19

4.2 Risk stratification 20

4.3 Constant treatment effect 20

4.4 Linear interaction 20

4.5 Restricted cubic splines 20

5 Adaptive model selection frequencies **21**

6 Discrimination and calibration for benefit **24**

7 Strong relative treatment effect **28**

8 Treatment interactions **31**

9 Correlated covariates **36**

10 Empirical illustration **42**

10.1 Prediction model 42

10.2 Bootstrap confidence intervals 42

11 References **43**

1 Notation

We observe RCT data (Z, X, Y), where for each patient $Z_i = 0, 1$ is the treatment status, $Y_i = 0, 1$ is the observed outcome and X_i is a set of covariates measured. Let $\{Y_i(z), z = 0, 1\}$ denote the unobservable potential outcomes. We observe $Y_i = Z_iY_i(1) + (1 - Z_i)Y_i(0)$. We are interested in predicting the conditional average treatment effect (CATE),

$$\tau(x) = E\{Y(0) - Y(1)|X = x\}$$

Assuming that (Z, X, Y) is a random sample from the target population and that $\big(Y(0), Y(1)\big) \perp\!\!\!\perp Z|X$, as we are in the RCT setting, we can predict CATE from

$$\begin{aligned}\tau(x) &= E\{Y(0) \mid X = x\} - E\{Y(1) \mid X = x\}\\ &= E\{Y \mid X = x, Z = 0\} - E\{Y \mid X = x, Z = 1\}\end{aligned}$$

Based on an estimate of baseline risk

$$E\{Y \mid X = x, Z = 0\} = g\big(\hat{lp}(x)\big)$$

with $\hat{u} = \hat{lp}(x) = x^t\hat{\beta}$ the linear predictor and g the link function, we predict CATE from

$$\hat{\tau}(x) = g\big(f(\hat{u}, 0)\big) - g\big(f(\hat{u}, 1)\big)$$

where $f(u, z)$ describes interactions of the baseline risk linear predictor with treatment.

2 Simulation settings

For all patients we observe covariates $X_1, \dots, X_8$, of which 4 are continuous and 4 are binary. More specifically,

$$\begin{aligned}X_1, \dots, X_4 &\sim N(0, 1)\\ X_5, \dots, X_8 &\sim B(1, 0.2)\end{aligned}$$

We first, generate the binary outcomes Y for the untreated patients ($Z = 0$), based on

$$P(Y(0) = 1 \mid X = x) = g(\beta_0 + \beta_1x_1 + \dots + \beta_8x_8) = g(lp_0), \tag{1}$$

where

$$g(x) = \frac{e^x}{1 + e^x}$$

For treated patients, outcomes are generated from:

$$P(Y = 1 \mid X = x, Z = 1) = g(lp_1) \tag{2}$$

where

$$lp_1 = \gamma_2(lp_0 - c)^2 + \gamma_1(lp_0 - c) + \gamma_0$$

2.1 Base-case scenario

The base-case scenario assumes a constant odds ratio of 0.8 in favor of treatment. The simulated datasets are of size $n = 4250$, where treatment is allocated at random using a 50/50 split (80% power for the detection of an unadjusted OR of 0.8, assuming an event rate of 20% in the untreated arm). Outcome incidence in the untreated population is set at 20%. For the development of the prediction model we use the model defined in (1) including a constant treatment effect. When doing predictions, Z is set to 0. The value of the true β is such that the above prediction model has an AUC of 0.75.

The previously defined targets are achieved when $\beta = (-2.08, 0.49, \ldots, 0.49)^t$. For the derivations in the treatment arm we use $\gamma = (\log(0.8), 1, 0)^t$.

2.2 Deviations from base-case

We deviate from the base-case scenario in two ways. First, we alter the overall target settings of sample size, overall treatment effect and prediction model AUC. In a second stage, we consider settings that violate the assumption of a constant relative treatment effect, using a model-based approach.

For the first part, we consider:

- Sample size:
  - $n = 1064$
  - $n = 17000$
- Overall treatment effect:
  - $OR = 0.5$
  - $OR = 1$
- Prediction performance:
  - $AUC = 0.65$
  - $AUC = 0.85$

We set the true risk model coefficients to be $\boldsymbol{\beta} = \big(-1.63, 0.26, \ldots, 0.26\big)^t$ for $AUC = 0.65$ and $\boldsymbol{\beta} = \big(-2.7, 0.82, \ldots, 0.82\big)^t$ for $AUC = 0.85$. In both cases, β_0 is selected so that an event rate of 20% is maintained in the control arm.

For the second part linear, quadratic and non-monotonic deviations from the assumption of constant relative effect are considered. We also consider different intensity levels of these deviations. Finally, constant absolute treatment-related harms are introduced, i.e. positive ($0.25 \times$ true average benefit), strong positive ($0.50 \times$ true average benefit) and negative ($-0.25 \times$ true average benefit; i.e. constant absolute treatment-related benefit). In case of true absent treatment effects, treatment-related harms are set to $1\%, 2\%$ and -1% for positive, strong positive and negative setting, respectively. The settings for these deviations are defined in Table S1.

Table S1: Scenario settings of the entire simulation study.

Analysis ID					Baseline risk									True treatment effect			
Scenario	Base	N	AUC	Treatment-related harm	b0	b1	b2	b3	b4	b5	b6	b7	b8	g0	g1	g2	c
1	absent	4,250	0.75	absent	-2.08	0.49	0.49	0.49	0.49	0.49	0.49	0.49	0.49	0.000	1.000	0.000	0
2	absent	4,250	0.75	moderate-positive	-2.08	0.49	0.49	0.49	0.49	0.49	0.49	0.49	0.49	0.000	1.000	0.000	0
3	absent	4,250	0.75	strong-positive	-2.08	0.49	0.49	0.49	0.49	0.49	0.49	0.49	0.49	0.000	1.000	0.000	0
4	absent	4,250	0.75	negative	-2.08	0.49	0.49	0.49	0.49	0.49	0.49	0.49	0.49	0.000	1.000	0.000	0
5	absent	4,250	0.65	absent	-1.63	0.26	0.26	0.26	0.26	0.26	0.26	0.26	0.26	0.000	1.000	0.000	0
6	absent	4,250	0.65	moderate-positive	-1.63	0.26	0.26	0.26	0.26	0.26	0.26	0.26	0.26	0.000	1.000	0.000	0
7	absent	4,250	0.65	strong-positive	-1.63	0.26	0.26	0.26	0.26	0.26	0.26	0.26	0.26	0.000	1.000	0.000	0
8	absent	4,250	0.65	negative	-1.63	0.26	0.26	0.26	0.26	0.26	0.26	0.26	0.26	0.000	1.000	0.000	0
9	absent	4,250	0.85	absent	-2.70	0.82	0.82	0.82	0.82	0.82	0.82	0.82	0.82	0.000	1.000	0.000	0
10	absent	4,250	0.85	moderate-positive	-2.70	0.82	0.82	0.82	0.82	0.82	0.82	0.82	0.82	0.000	1.000	0.000	0
11	absent	4,250	0.85	strong-positive	-2.70	0.82	0.82	0.82	0.82	0.82	0.82	0.82	0.82	0.000	1.000	0.000	0
12	absent	4,250	0.85	negative	-2.70	0.82	0.82	0.82	0.82	0.82	0.82	0.82	0.82	0.000	1.000	0.000	0
13	absent	1,063	0.75	absent	-2.08	0.49	0.49	0.49	0.49	0.49	0.49	0.49	0.49	0.000	1.000	0.000	0
14	absent	1,063	0.75	moderate-positive	-2.08	0.49	0.49	0.49	0.49	0.49	0.49	0.49	0.49	0.000	1.000	0.000	0
15	absent	1,063	0.75	strong-positive	-2.08	0.49	0.49	0.49	0.49	0.49	0.49	0.49	0.49	0.000	1.000	0.000	0
16	absent	1,063	0.75	negative	-2.08	0.49	0.49	0.49	0.49	0.49	0.49	0.49	0.49	0.000	1.000	0.000	0
17	absent	1,063	0.65	absent	-1.63	0.26	0.26	0.26	0.26	0.26	0.26	0.26	0.26	0.000	1.000	0.000	0
18	absent	1,063	0.65	moderate-positive	-1.63	0.26	0.26	0.26	0.26	0.26	0.26	0.26	0.26	0.000	1.000	0.000	0
19	absent	1,063	0.65	strong-positive	-1.63	0.26	0.26	0.26	0.26	0.26	0.26	0.26	0.26	0.000	1.000	0.000	0
20	absent	1,063	0.65	negative	-1.63	0.26	0.26	0.26	0.26	0.26	0.26	0.26	0.26	0.000	1.000	0.000	0
21	absent	1,063	0.85	absent	-2.70	0.82	0.82	0.82	0.82	0.82	0.82	0.82	0.82	0.000	1.000	0.000	0
22	absent	1,063	0.85	moderate-positive	-2.70	0.82	0.82	0.82	0.82	0.82	0.82	0.82	0.82	0.000	1.000	0.000	0
23	absent	1,063	0.85	strong-positive	-2.70	0.82	0.82	0.82	0.82	0.82	0.82	0.82	0.82	0.000	1.000	0.000	0
24	absent	1,063	0.85	negative	-2.70	0.82	0.82	0.82	0.82	0.82	0.82	0.82	0.82	0.000	1.000	0.000	0
25	absent	17,000	0.75	absent	-2.08	0.49	0.49	0.49	0.49	0.49	0.49	0.49	0.49	0.000	1.000	0.000	0
26	absent	17,000	0.75	moderate-positive	-2.08	0.49	0.49	0.49	0.49	0.49	0.49	0.49	0.49	0.000	1.000	0.000	0
27	absent	17,000	0.75	strong-positive	-2.08	0.49	0.49	0.49	0.49	0.49	0.49	0.49	0.49	0.000	1.000	0.000	0
28	absent	17,000	0.75	negative	-2.08	0.49	0.49	0.49	0.49	0.49	0.49	0.49	0.49	0.000	1.000	0.000	0
29	absent	17,000	0.65	absent	-1.63	0.26	0.26	0.26	0.26	0.26	0.26	0.26	0.26	0.000	1.000	0.000	0
30	absent	17,000	0.65	moderate-positive	-1.63	0.26	0.26	0.26	0.26	0.26	0.26	0.26	0.26	0.000	1.000	0.000	0
31	absent	17,000	0.65	strong-positive	-1.63	0.26	0.26	0.26	0.26	0.26	0.26	0.26	0.26	0.000	1.000	0.000	0
32	absent	17,000	0.65	negative	-1.63	0.26	0.26	0.26	0.26	0.26	0.26	0.26	0.26	0.000	1.000	0.000	0
33	absent	17,000	0.85	absent	-2.70	0.82	0.82	0.82	0.82	0.82	0.82	0.82	0.82	0.000	1.000	0.000	0
34	absent	17,000	0.85	moderate-positive	-2.70	0.82	0.82	0.82	0.82	0.82	0.82	0.82	0.82	0.000	1.000	0.000	0
35	absent	17,000	0.85	strong-positive	-2.70	0.82	0.82	0.82	0.82	0.82	0.82	0.82	0.82	0.000	1.000	0.000	0
36	absent	17,000	0.85	negative	-2.70	0.82	0.82	0.82	0.82	0.82	0.82	0.82	0.82	0.000	1.000	0.000	0
37	absent	4,250	0.75	absent	-2.08	0.49	0.49	0.49	0.49	0.49	0.49	0.49	0.49	-0.060	0.947	0.000	0
38	absent	4,250	0.75	moderate-positive	-2.08	0.49	0.49	0.49	0.49	0.49	0.49	0.49	0.49	-0.060	0.947	0.000	0
39	absent	4,250	0.75	strong-positive	-2.08	0.49	0.49	0.49	0.49	0.49	0.49	0.49	0.49	-0.060	0.947	0.000	0
40	absent	4,250	0.75	negative	-2.08	0.49	0.49	0.49	0.49	0.49	0.49	0.49	0.49	-0.060	0.947	0.000	0
41	absent	4,250	0.65	absent	-1.63	0.26	0.26	0.26	0.26	0.26	0.26	0.26	0.26	-0.080	0.934	0.000	0

42	absent	4,250	0.65	moderate-positive	-1.63	0.26	0.26	0.26	0.26	0.26	0.26	0.26	0.26	-0.080	0.934	0.000	0
43	absent	4,250	0.65	strong-positive	-1.63	0.26	0.26	0.26	0.26	0.26	0.26	0.26	0.26	-0.080	0.934	0.000	0
44	absent	4,250	0.65	negative	-1.63	0.26	0.26	0.26	0.26	0.26	0.26	0.26	0.26	-0.080	0.934	0.000	0
45	absent	4,250	0.85	absent	-2.70	0.82	0.82	0.82	0.82	0.82	0.82	0.82	0.82	-0.070	0.930	0.000	0
46	absent	4,250	0.85	moderate-positive	-2.70	0.82	0.82	0.82	0.82	0.82	0.82	0.82	0.82	-0.070	0.930	0.000	0
47	absent	4,250	0.85	strong-positive	-2.70	0.82	0.82	0.82	0.82	0.82	0.82	0.82	0.82	-0.070	0.930	0.000	0
48	absent	4,250	0.85	negative	-2.70	0.82	0.82	0.82	0.82	0.82	0.82	0.82	0.82	-0.070	0.930	0.000	0
49	absent	1,063	0.75	absent	-2.08	0.49	0.49	0.49	0.49	0.49	0.49	0.49	0.49	-0.060	0.947	0.000	0
50	absent	1,063	0.75	moderate-positive	-2.08	0.49	0.49	0.49	0.49	0.49	0.49	0.49	0.49	-0.060	0.947	0.000	0
51	absent	1,063	0.75	strong-positive	-2.08	0.49	0.49	0.49	0.49	0.49	0.49	0.49	0.49	-0.060	0.947	0.000	0
52	absent	1,063	0.75	negative	-2.08	0.49	0.49	0.49	0.49	0.49	0.49	0.49	0.49	-0.060	0.947	0.000	0
53	absent	1,063	0.65	absent	-1.63	0.26	0.26	0.26	0.26	0.26	0.26	0.26	0.26	-0.080	0.934	0.000	0
54	absent	1,063	0.65	moderate-positive	-1.63	0.26	0.26	0.26	0.26	0.26	0.26	0.26	0.26	-0.080	0.934	0.000	0
55	absent	1,063	0.65	strong-positive	-1.63	0.26	0.26	0.26	0.26	0.26	0.26	0.26	0.26	-0.080	0.934	0.000	0
56	absent	1,063	0.65	negative	-1.63	0.26	0.26	0.26	0.26	0.26	0.26	0.26	0.26	-0.080	0.934	0.000	0
57	absent	1,063	0.85	absent	-2.70	0.82	0.82	0.82	0.82	0.82	0.82	0.82	0.82	-0.070	0.930	0.000	0
58	absent	1,063	0.85	moderate-positive	-2.70	0.82	0.82	0.82	0.82	0.82	0.82	0.82	0.82	-0.070	0.930	0.000	0
59	absent	1,063	0.85	strong-positive	-2.70	0.82	0.82	0.82	0.82	0.82	0.82	0.82	0.82	-0.070	0.930	0.000	0
60	absent	1,063	0.85	negative	-2.70	0.82	0.82	0.82	0.82	0.82	0.82	0.82	0.82	-0.070	0.930	0.000	0
61	absent	17,000	0.75	absent	-2.08	0.49	0.49	0.49	0.49	0.49	0.49	0.49	0.49	-0.060	0.947	0.000	0
62	absent	17,000	0.75	moderate-positive	-2.08	0.49	0.49	0.49	0.49	0.49	0.49	0.49	0.49	-0.060	0.947	0.000	0
63	absent	17,000	0.75	strong-positive	-2.08	0.49	0.49	0.49	0.49	0.49	0.49	0.49	0.49	-0.060	0.947	0.000	0
64	absent	17,000	0.75	negative	-2.08	0.49	0.49	0.49	0.49	0.49	0.49	0.49	0.49	-0.060	0.947	0.000	0
65	absent	17,000	0.65	absent	-1.63	0.26	0.26	0.26	0.26	0.26	0.26	0.26	0.26	-0.080	0.934	0.000	0
66	absent	17,000	0.65	moderate-positive	-1.63	0.26	0.26	0.26	0.26	0.26	0.26	0.26	0.26	-0.080	0.934	0.000	0
67	absent	17,000	0.65	strong-positive	-1.63	0.26	0.26	0.26	0.26	0.26	0.26	0.26	0.26	-0.080	0.934	0.000	0
68	absent	17,000	0.65	negative	-1.63	0.26	0.26	0.26	0.26	0.26	0.26	0.26	0.26	-0.080	0.934	0.000	0
69	absent	17,000	0.85	absent	-2.70	0.82	0.82	0.82	0.82	0.82	0.82	0.82	0.82	-0.070	0.930	0.000	0
70	absent	17,000	0.85	moderate-positive	-2.70	0.82	0.82	0.82	0.82	0.82	0.82	0.82	0.82	-0.070	0.930	0.000	0
71	absent	17,000	0.85	strong-positive	-2.70	0.82	0.82	0.82	0.82	0.82	0.82	0.82	0.82	-0.070	0.930	0.000	0
72	absent	17,000	0.85	negative	-2.70	0.82	0.82	0.82	0.82	0.82	0.82	0.82	0.82	-0.070	0.930	0.000	0
73	absent	4,250	0.75	absent	-2.08	0.49	0.49	0.49	0.49	0.49	0.49	0.49	0.49	-0.250	0.796	0.000	0
74	absent	4,250	0.75	moderate-positive	-2.08	0.49	0.49	0.49	0.49	0.49	0.49	0.49	0.49	-0.250	0.796	0.000	0
75	absent	4,250	0.75	strong-positive	-2.08	0.49	0.49	0.49	0.49	0.49	0.49	0.49	0.49	-0.250	0.796	0.000	0
76	absent	4,250	0.75	negative	-2.08	0.49	0.49	0.49	0.49	0.49	0.49	0.49	0.49	-0.250	0.796	0.000	0
77	absent	4,250	0.65	absent	-1.63	0.26	0.26	0.26	0.26	0.26	0.26	0.26	0.26	-0.290	0.776	0.000	0
78	absent	4,250	0.65	moderate-positive	-1.63	0.26	0.26	0.26	0.26	0.26	0.26	0.26	0.26	-0.290	0.776	0.000	0
79	absent	4,250	0.65	strong-positive	-1.63	0.26	0.26	0.26	0.26	0.26	0.26	0.26	0.26	-0.290	0.776	0.000	0
80	absent	4,250	0.65	negative	-1.63	0.26	0.26	0.26	0.26	0.26	0.26	0.26	0.26	-0.290	0.776	0.000	0
81	absent	4,250	0.85	absent	-2.70	0.82	0.82	0.82	0.82	0.82	0.82	0.82	0.82	-0.220	0.785	0.000	0
82	absent	4,250	0.85	moderate-positive	-2.70	0.82	0.82	0.82	0.82	0.82	0.82	0.82	0.82	-0.220	0.785	0.000	0
83	absent	4,250	0.85	strong-positive	-2.70	0.82	0.82	0.82	0.82	0.82	0.82	0.82	0.82	-0.220	0.785	0.000	0
84	absent	4,250	0.85	negative	-2.70	0.82	0.82	0.82	0.82	0.82	0.82	0.82	0.82	-0.220	0.785	0.000	0
85	absent	1,063	0.75	absent	-2.08	0.49	0.49	0.49	0.49	0.49	0.49	0.49	0.49	-0.250	0.796	0.000	0
86	absent	1,063	0.75	moderate-positive	-2.08	0.49	0.49	0.49	0.49	0.49	0.49	0.49	0.49	-0.250	0.796	0.000	0

87	absent	1,063	0.75	strong-positive	-2.08	0.49	0.49	0.49	0.49	0.49	0.49	0.49	0.49	-0.250	0.796	0.000	0
88	absent	1,063	0.75	negative	-2.08	0.49	0.49	0.49	0.49	0.49	0.49	0.49	0.49	-0.250	0.796	0.000	0
89	absent	1,063	0.65	absent	-1.63	0.26	0.26	0.26	0.26	0.26	0.26	0.26	0.26	-0.290	0.776	0.000	0
90	absent	1,063	0.65	moderate-positive	-1.63	0.26	0.26	0.26	0.26	0.26	0.26	0.26	0.26	-0.290	0.776	0.000	0
91	absent	1,063	0.65	strong-positive	-1.63	0.26	0.26	0.26	0.26	0.26	0.26	0.26	0.26	-0.290	0.776	0.000	0
92	absent	1,063	0.65	negative	-1.63	0.26	0.26	0.26	0.26	0.26	0.26	0.26	0.26	-0.290	0.776	0.000	0
93	absent	1,063	0.85	absent	-2.70	0.82	0.82	0.82	0.82	0.82	0.82	0.82	0.82	-0.220	0.785	0.000	0
94	absent	1,063	0.85	moderate-positive	-2.70	0.82	0.82	0.82	0.82	0.82	0.82	0.82	0.82	-0.220	0.785	0.000	0
95	absent	1,063	0.85	strong-positive	-2.70	0.82	0.82	0.82	0.82	0.82	0.82	0.82	0.82	-0.220	0.785	0.000	0
96	absent	1,063	0.85	negative	-2.70	0.82	0.82	0.82	0.82	0.82	0.82	0.82	0.82	-0.220	0.785	0.000	0
97	absent	17,000	0.75	absent	-2.08	0.49	0.49	0.49	0.49	0.49	0.49	0.49	0.49	-0.250	0.796	0.000	0
98	absent	17,000	0.75	moderate-positive	-2.08	0.49	0.49	0.49	0.49	0.49	0.49	0.49	0.49	-0.250	0.796	0.000	0
99	absent	17,000	0.75	strong-positive	-2.08	0.49	0.49	0.49	0.49	0.49	0.49	0.49	0.49	-0.250	0.796	0.000	0
100	absent	17,000	0.75	negative	-2.08	0.49	0.49	0.49	0.49	0.49	0.49	0.49	0.49	-0.250	0.796	0.000	0
101	absent	17,000	0.65	absent	-1.63	0.26	0.26	0.26	0.26	0.26	0.26	0.26	0.26	-0.290	0.776	0.000	0
102	absent	17,000	0.65	moderate-positive	-1.63	0.26	0.26	0.26	0.26	0.26	0.26	0.26	0.26	-0.290	0.776	0.000	0
103	absent	17,000	0.65	strong-positive	-1.63	0.26	0.26	0.26	0.26	0.26	0.26	0.26	0.26	-0.290	0.776	0.000	0
104	absent	17,000	0.65	negative	-1.63	0.26	0.26	0.26	0.26	0.26	0.26	0.26	0.26	-0.290	0.776	0.000	0
105	absent	17,000	0.85	absent	-2.70	0.82	0.82	0.82	0.82	0.82	0.82	0.82	0.82	-0.220	0.785	0.000	0
106	absent	17,000	0.85	moderate-positive	-2.70	0.82	0.82	0.82	0.82	0.82	0.82	0.82	0.82	-0.220	0.785	0.000	0
107	absent	17,000	0.85	strong-positive	-2.70	0.82	0.82	0.82	0.82	0.82	0.82	0.82	0.82	-0.220	0.785	0.000	0
108	absent	17,000	0.85	negative	-2.70	0.82	0.82	0.82	0.82	0.82	0.82	0.82	0.82	-0.220	0.785	0.000	0
109	absent	4,250	0.75	absent	-2.08	0.49	0.49	0.49	0.49	0.49	0.49	0.49	0.49	-4.810	1.000	-0.013	-5
110	absent	4,250	0.75	moderate-positive	-2.08	0.49	0.49	0.49	0.49	0.49	0.49	0.49	0.49	-4.810	1.000	-0.013	-5
111	absent	4,250	0.75	strong-positive	-2.08	0.49	0.49	0.49	0.49	0.49	0.49	0.49	0.49	-4.810	1.000	-0.013	-5
112	absent	4,250	0.75	negative	-2.08	0.49	0.49	0.49	0.49	0.49	0.49	0.49	0.49	-4.810	1.000	-0.013	-5
113	absent	4,250	0.65	absent	-1.63	0.26	0.26	0.26	0.26	0.26	0.26	0.26	0.26	-4.770	1.000	-0.016	-5
114	absent	4,250	0.65	moderate-positive	-1.63	0.26	0.26	0.26	0.26	0.26	0.26	0.26	0.26	-4.770	1.000	-0.016	-5
115	absent	4,250	0.65	strong-positive	-1.63	0.26	0.26	0.26	0.26	0.26	0.26	0.26	0.26	-4.770	1.000	-0.016	-5
116	absent	4,250	0.65	negative	-1.63	0.26	0.26	0.26	0.26	0.26	0.26	0.26	0.26	-4.770	1.000	-0.016	-5
117	absent	4,250	0.85	absent	-2.70	0.82	0.82	0.82	0.82	0.82	0.82	0.82	0.82	-4.710	1.000	-0.016	-5
118	absent	4,250	0.85	moderate-positive	-2.70	0.82	0.82	0.82	0.82	0.82	0.82	0.82	0.82	-4.710	1.000	-0.016	-5
119	absent	4,250	0.85	strong-positive	-2.70	0.82	0.82	0.82	0.82	0.82	0.82	0.82	0.82	-4.710	1.000	-0.016	-5
120	absent	4,250	0.85	negative	-2.70	0.82	0.82	0.82	0.82	0.82	0.82	0.82	0.82	-4.710	1.000	-0.016	-5
121	absent	1,063	0.75	absent	-2.08	0.49	0.49	0.49	0.49	0.49	0.49	0.49	0.49	-4.810	1.000	-0.013	-5
122	absent	1,063	0.75	moderate-positive	-2.08	0.49	0.49	0.49	0.49	0.49	0.49	0.49	0.49	-4.810	1.000	-0.013	-5
123	absent	1,063	0.75	strong-positive	-2.08	0.49	0.49	0.49	0.49	0.49	0.49	0.49	0.49	-4.810	1.000	-0.013	-5
124	absent	1,063	0.75	negative	-2.08	0.49	0.49	0.49	0.49	0.49	0.49	0.49	0.49	-4.810	1.000	-0.013	-5
125	absent	1,063	0.65	absent	-1.63	0.26	0.26	0.26	0.26	0.26	0.26	0.26	0.26	-4.770	1.000	-0.016	-5
126	absent	1,063	0.65	moderate-positive	-1.63	0.26	0.26	0.26	0.26	0.26	0.26	0.26	0.26	-4.770	1.000	-0.016	-5
127	absent	1,063	0.65	strong-positive	-1.63	0.26	0.26	0.26	0.26	0.26	0.26	0.26	0.26	-4.770	1.000	-0.016	-5
128	absent	1,063	0.65	negative	-1.63	0.26	0.26	0.26	0.26	0.26	0.26	0.26	0.26	-4.770	1.000	-0.016	-5
129	absent	1,063	0.85	absent	-2.70	0.82	0.82	0.82	0.82	0.82	0.82	0.82	0.82	-4.710	1.000	-0.016	-5
130	absent	1,063	0.85	moderate-positive	-2.70	0.82	0.82	0.82	0.82	0.82	0.82	0.82	0.82	-4.710	1.000	-0.016	-5
131	absent	1,063	0.85	strong-positive	-2.70	0.82	0.82	0.82	0.82	0.82	0.82	0.82	0.82	-4.710	1.000	-0.016	-5

132	absent	1,063	0.85	negative	-2.70	0.82	0.82	0.82	0.82	0.82	0.82	0.82	0.82	-4.710	1.000	-0.016	-5
133	absent	17,000	0.75	absent	-2.08	0.49	0.49	0.49	0.49	0.49	0.49	0.49	0.49	-4.810	1.000	-0.013	-5
134	absent	17,000	0.75	moderate-positive	-2.08	0.49	0.49	0.49	0.49	0.49	0.49	0.49	0.49	-4.810	1.000	-0.013	-5
135	absent	17,000	0.75	strong-positive	-2.08	0.49	0.49	0.49	0.49	0.49	0.49	0.49	0.49	-4.810	1.000	-0.013	-5
136	absent	17,000	0.75	negative	-2.08	0.49	0.49	0.49	0.49	0.49	0.49	0.49	0.49	-4.810	1.000	-0.013	-5
137	absent	17,000	0.65	absent	-1.63	0.26	0.26	0.26	0.26	0.26	0.26	0.26	0.26	-4.770	1.000	-0.016	-5
138	absent	17,000	0.65	moderate-positive	-1.63	0.26	0.26	0.26	0.26	0.26	0.26	0.26	0.26	-4.770	1.000	-0.016	-5
139	absent	17,000	0.65	strong-positive	-1.63	0.26	0.26	0.26	0.26	0.26	0.26	0.26	0.26	-4.770	1.000	-0.016	-5
140	absent	17,000	0.65	negative	-1.63	0.26	0.26	0.26	0.26	0.26	0.26	0.26	0.26	-4.770	1.000	-0.016	-5
141	absent	17,000	0.85	absent	-2.70	0.82	0.82	0.82	0.82	0.82	0.82	0.82	0.82	-4.710	1.000	-0.016	-5
142	absent	17,000	0.85	moderate-positive	-2.70	0.82	0.82	0.82	0.82	0.82	0.82	0.82	0.82	-4.710	1.000	-0.016	-5
143	absent	17,000	0.85	strong-positive	-2.70	0.82	0.82	0.82	0.82	0.82	0.82	0.82	0.82	-4.710	1.000	-0.016	-5
144	absent	17,000	0.85	negative	-2.70	0.82	0.82	0.82	0.82	0.82	0.82	0.82	0.82	-4.710	1.000	-0.016	-5
145	absent	4,250	0.75	absent	-2.08	0.49	0.49	0.49	0.49	0.49	0.49	0.49	0.49	-4.220	1.000	-0.052	-5
146	absent	4,250	0.75	moderate-positive	-2.08	0.49	0.49	0.49	0.49	0.49	0.49	0.49	0.49	-4.220	1.000	-0.052	-5
147	absent	4,250	0.75	strong-positive	-2.08	0.49	0.49	0.49	0.49	0.49	0.49	0.49	0.49	-4.220	1.000	-0.052	-5
148	absent	4,250	0.75	negative	-2.08	0.49	0.49	0.49	0.49	0.49	0.49	0.49	0.49	-4.220	1.000	-0.052	-5
149	absent	4,250	0.65	absent	-1.63	0.26	0.26	0.26	0.26	0.26	0.26	0.26	0.26	-4.160	1.000	-0.059	-5
150	absent	4,250	0.65	moderate-positive	-1.63	0.26	0.26	0.26	0.26	0.26	0.26	0.26	0.26	-4.160	1.000	-0.059	-5
151	absent	4,250	0.65	strong-positive	-1.63	0.26	0.26	0.26	0.26	0.26	0.26	0.26	0.26	-4.160	1.000	-0.059	-5
152	absent	4,250	0.65	negative	-1.63	0.26	0.26	0.26	0.26	0.26	0.26	0.26	0.26	-4.160	1.000	-0.059	-5
153	absent	4,250	0.85	absent	-2.70	0.82	0.82	0.82	0.82	0.82	0.82	0.82	0.82	-3.950	1.000	-0.059	-5
154	absent	4,250	0.85	moderate-positive	-2.70	0.82	0.82	0.82	0.82	0.82	0.82	0.82	0.82	-3.950	1.000	-0.059	-5
155	absent	4,250	0.85	strong-positive	-2.70	0.82	0.82	0.82	0.82	0.82	0.82	0.82	0.82	-3.950	1.000	-0.059	-5
156	absent	4,250	0.85	negative	-2.70	0.82	0.82	0.82	0.82	0.82	0.82	0.82	0.82	-3.950	1.000	-0.059	-5
157	absent	1,063	0.75	absent	-2.08	0.49	0.49	0.49	0.49	0.49	0.49	0.49	0.49	-4.220	1.000	-0.052	-5
158	absent	1,063	0.75	moderate-positive	-2.08	0.49	0.49	0.49	0.49	0.49	0.49	0.49	0.49	-4.220	1.000	-0.052	-5
159	absent	1,063	0.75	strong-positive	-2.08	0.49	0.49	0.49	0.49	0.49	0.49	0.49	0.49	-4.220	1.000	-0.052	-5
160	absent	1,063	0.75	negative	-2.08	0.49	0.49	0.49	0.49	0.49	0.49	0.49	0.49	-4.220	1.000	-0.052	-5
161	absent	1,063	0.65	absent	-1.63	0.26	0.26	0.26	0.26	0.26	0.26	0.26	0.26	-4.160	1.000	-0.059	-5
162	absent	1,063	0.65	moderate-positive	-1.63	0.26	0.26	0.26	0.26	0.26	0.26	0.26	0.26	-4.160	1.000	-0.059	-5
163	absent	1,063	0.65	strong-positive	-1.63	0.26	0.26	0.26	0.26	0.26	0.26	0.26	0.26	-4.160	1.000	-0.059	-5
164	absent	1,063	0.65	negative	-1.63	0.26	0.26	0.26	0.26	0.26	0.26	0.26	0.26	-4.160	1.000	-0.059	-5
165	absent	1,063	0.85	absent	-2.70	0.82	0.82	0.82	0.82	0.82	0.82	0.82	0.82	-3.950	1.000	-0.059	-5
166	absent	1,063	0.85	moderate-positive	-2.70	0.82	0.82	0.82	0.82	0.82	0.82	0.82	0.82	-3.950	1.000	-0.059	-5
167	absent	1,063	0.85	strong-positive	-2.70	0.82	0.82	0.82	0.82	0.82	0.82	0.82	0.82	-3.950	1.000	-0.059	-5
168	absent	1,063	0.85	negative	-2.70	0.82	0.82	0.82	0.82	0.82	0.82	0.82	0.82	-3.950	1.000	-0.059	-5
169	absent	17,000	0.75	absent	-2.08	0.49	0.49	0.49	0.49	0.49	0.49	0.49	0.49	-4.220	1.000	-0.052	-5
170	absent	17,000	0.75	moderate-positive	-2.08	0.49	0.49	0.49	0.49	0.49	0.49	0.49	0.49	-4.220	1.000	-0.052	-5
171	absent	17,000	0.75	strong-positive	-2.08	0.49	0.49	0.49	0.49	0.49	0.49	0.49	0.49	-4.220	1.000	-0.052	-5
172	absent	17,000	0.75	negative	-2.08	0.49	0.49	0.49	0.49	0.49	0.49	0.49	0.49	-4.220	1.000	-0.052	-5
173	absent	17,000	0.65	absent	-1.63	0.26	0.26	0.26	0.26	0.26	0.26	0.26	0.26	-4.160	1.000	-0.059	-5
174	absent	17,000	0.65	moderate-positive	-1.63	0.26	0.26	0.26	0.26	0.26	0.26	0.26	0.26	-4.160	1.000	-0.059	-5
175	absent	17,000	0.65	strong-positive	-1.63	0.26	0.26	0.26	0.26	0.26	0.26	0.26	0.26	-4.160	1.000	-0.059	-5
176	absent	17,000	0.65	negative	-1.63	0.26	0.26	0.26	0.26	0.26	0.26	0.26	0.26	-4.160	1.000	-0.059	-5

177	absent	17,000	0.85	absent	-2.70	0.82	0.82	0.82	0.82	0.82	0.82	0.82	0.82	-3.950	1.000	-0.059	-5
178	absent	17,000	0.85	moderate-positive	-2.70	0.82	0.82	0.82	0.82	0.82	0.82	0.82	0.82	-3.950	1.000	-0.059	-5
179	absent	17,000	0.85	strong-positive	-2.70	0.82	0.82	0.82	0.82	0.82	0.82	0.82	0.82	-3.950	1.000	-0.059	-5
180	absent	17,000	0.85	negative	-2.70	0.82	0.82	0.82	0.82	0.82	0.82	0.82	0.82	-3.950	1.000	-0.059	-5
181	absent	4,250	0.75	absent	-2.08	0.49	0.49	0.49	0.49	0.49	0.49	0.49	0.49	2.490	4.210	0.540	0
182	absent	4,250	0.75	moderate-positive	-2.08	0.49	0.49	0.49	0.49	0.49	0.49	0.49	0.49	2.490	4.210	0.540	0
183	absent	4,250	0.75	strong-positive	-2.08	0.49	0.49	0.49	0.49	0.49	0.49	0.49	0.49	2.490	4.210	0.540	0
184	absent	4,250	0.75	negative	-2.08	0.49	0.49	0.49	0.49	0.49	0.49	0.49	0.49	2.490	4.210	0.540	0
185	absent	4,250	0.65	absent	-1.63	0.26	0.26	0.26	0.26	0.26	0.26	0.26	0.26	2.150	3.320	0.380	0
186	absent	4,250	0.65	moderate-positive	-1.63	0.26	0.26	0.26	0.26	0.26	0.26	0.26	0.26	2.150	3.320	0.380	0
187	absent	4,250	0.65	strong-positive	-1.63	0.26	0.26	0.26	0.26	0.26	0.26	0.26	0.26	2.150	3.320	0.380	0
188	absent	4,250	0.65	negative	-1.63	0.26	0.26	0.26	0.26	0.26	0.26	0.26	0.26	2.150	3.320	0.380	0
189	absent	4,250	0.85	absent	-2.70	0.82	0.82	0.82	0.82	0.82	0.82	0.82	0.82	1.740	4.100	0.550	0
190	absent	4,250	0.85	moderate-positive	-2.70	0.82	0.82	0.82	0.82	0.82	0.82	0.82	0.82	1.740	4.100	0.550	0
191	absent	4,250	0.85	strong-positive	-2.70	0.82	0.82	0.82	0.82	0.82	0.82	0.82	0.82	1.740	4.100	0.550	0
192	absent	4,250	0.85	negative	-2.70	0.82	0.82	0.82	0.82	0.82	0.82	0.82	0.82	1.740	4.100	0.550	0
193	absent	1,063	0.75	absent	-2.08	0.49	0.49	0.49	0.49	0.49	0.49	0.49	0.49	2.490	4.210	0.540	0
194	absent	1,063	0.75	moderate-positive	-2.08	0.49	0.49	0.49	0.49	0.49	0.49	0.49	0.49	2.490	4.210	0.540	0
195	absent	1,063	0.75	strong-positive	-2.08	0.49	0.49	0.49	0.49	0.49	0.49	0.49	0.49	2.490	4.210	0.540	0
196	absent	1,063	0.75	negative	-2.08	0.49	0.49	0.49	0.49	0.49	0.49	0.49	0.49	2.490	4.210	0.540	0
197	absent	1,063	0.65	absent	-1.63	0.26	0.26	0.26	0.26	0.26	0.26	0.26	0.26	2.150	3.320	0.380	0
198	absent	1,063	0.65	moderate-positive	-1.63	0.26	0.26	0.26	0.26	0.26	0.26	0.26	0.26	2.150	3.320	0.380	0
199	absent	1,063	0.65	strong-positive	-1.63	0.26	0.26	0.26	0.26	0.26	0.26	0.26	0.26	2.150	3.320	0.380	0
200	absent	1,063	0.65	negative	-1.63	0.26	0.26	0.26	0.26	0.26	0.26	0.26	0.26	2.150	3.320	0.380	0
201	absent	1,063	0.85	absent	-2.70	0.82	0.82	0.82	0.82	0.82	0.82	0.82	0.82	1.740	4.100	0.550	0
202	absent	1,063	0.85	moderate-positive	-2.70	0.82	0.82	0.82	0.82	0.82	0.82	0.82	0.82	1.740	4.100	0.550	0
203	absent	1,063	0.85	strong-positive	-2.70	0.82	0.82	0.82	0.82	0.82	0.82	0.82	0.82	1.740	4.100	0.550	0
204	absent	1,063	0.85	negative	-2.70	0.82	0.82	0.82	0.82	0.82	0.82	0.82	0.82	1.740	4.100	0.550	0
205	absent	17,000	0.75	absent	-2.08	0.49	0.49	0.49	0.49	0.49	0.49	0.49	0.49	2.490	4.210	0.540	0
206	absent	17,000	0.75	moderate-positive	-2.08	0.49	0.49	0.49	0.49	0.49	0.49	0.49	0.49	2.490	4.210	0.540	0
207	absent	17,000	0.75	strong-positive	-2.08	0.49	0.49	0.49	0.49	0.49	0.49	0.49	0.49	2.490	4.210	0.540	0
208	absent	17,000	0.75	negative	-2.08	0.49	0.49	0.49	0.49	0.49	0.49	0.49	0.49	2.490	4.210	0.540	0
209	absent	17,000	0.65	absent	-1.63	0.26	0.26	0.26	0.26	0.26	0.26	0.26	0.26	2.150	3.320	0.380	0
210	absent	17,000	0.65	moderate-positive	-1.63	0.26	0.26	0.26	0.26	0.26	0.26	0.26	0.26	2.150	3.320	0.380	0
211	absent	17,000	0.65	strong-positive	-1.63	0.26	0.26	0.26	0.26	0.26	0.26	0.26	0.26	2.150	3.320	0.380	0
212	absent	17,000	0.65	negative	-1.63	0.26	0.26	0.26	0.26	0.26	0.26	0.26	0.26	2.150	3.320	0.380	0
213	absent	17,000	0.85	absent	-2.70	0.82	0.82	0.82	0.82	0.82	0.82	0.82	0.82	1.740	4.100	0.550	0
214	absent	17,000	0.85	moderate-positive	-2.70	0.82	0.82	0.82	0.82	0.82	0.82	0.82	0.82	1.740	4.100	0.550	0
215	absent	17,000	0.85	strong-positive	-2.70	0.82	0.82	0.82	0.82	0.82	0.82	0.82	0.82	1.740	4.100	0.550	0
216	absent	17,000	0.85	negative	-2.70	0.82	0.82	0.82	0.82	0.82	0.82	0.82	0.82	1.740	4.100	0.550	0
217	moderate	4,250	0.75	absent	-2.08	0.49	0.49	0.49	0.49	0.49	0.49	0.49	0.49	-0.223	1.000	0.000	0
218	moderate	4,250	0.75	moderate-positive	-2.08	0.49	0.49	0.49	0.49	0.49	0.49	0.49	0.49	-0.223	1.000	0.000	0
219	moderate	4,250	0.75	strong-positive	-2.08	0.49	0.49	0.49	0.49	0.49	0.49	0.49	0.49	-0.223	1.000	0.000	0
220	moderate	4,250	0.75	negative	-2.08	0.49	0.49	0.49	0.49	0.49	0.49	0.49	0.49	-0.223	1.000	0.000	0
221	moderate	4,250	0.65	absent	-1.63	0.26	0.26	0.26	0.26	0.26	0.26	0.26	0.26	-0.223	1.000	0.000	0

222	moderate	4,250	0.65	moderate-positive	-1.63	0.26	0.26	0.26	0.26	0.26	0.26	0.26	0.26	-0.223	1.000	0.000	0
223	moderate	4,250	0.65	strong-positive	-1.63	0.26	0.26	0.26	0.26	0.26	0.26	0.26	0.26	-0.223	1.000	0.000	0
224	moderate	4,250	0.65	negative	-1.63	0.26	0.26	0.26	0.26	0.26	0.26	0.26	0.26	-0.223	1.000	0.000	0
225	moderate	4,250	0.85	absent	-2.70	0.82	0.82	0.82	0.82	0.82	0.82	0.82	0.82	-0.223	1.000	0.000	0
226	moderate	4,250	0.85	moderate-positive	-2.70	0.82	0.82	0.82	0.82	0.82	0.82	0.82	0.82	-0.223	1.000	0.000	0
227	moderate	4,250	0.85	strong-positive	-2.70	0.82	0.82	0.82	0.82	0.82	0.82	0.82	0.82	-0.223	1.000	0.000	0
228	moderate	4,250	0.85	negative	-2.70	0.82	0.82	0.82	0.82	0.82	0.82	0.82	0.82	-0.223	1.000	0.000	0
229	moderate	1,063	0.75	absent	-2.08	0.49	0.49	0.49	0.49	0.49	0.49	0.49	0.49	-0.223	1.000	0.000	0
230	moderate	1,063	0.75	moderate-positive	-2.08	0.49	0.49	0.49	0.49	0.49	0.49	0.49	0.49	-0.223	1.000	0.000	0
231	moderate	1,063	0.75	strong-positive	-2.08	0.49	0.49	0.49	0.49	0.49	0.49	0.49	0.49	-0.223	1.000	0.000	0
232	moderate	1,063	0.75	negative	-2.08	0.49	0.49	0.49	0.49	0.49	0.49	0.49	0.49	-0.223	1.000	0.000	0
233	moderate	1,063	0.65	absent	-1.63	0.26	0.26	0.26	0.26	0.26	0.26	0.26	0.26	-0.223	1.000	0.000	0
234	moderate	1,063	0.65	moderate-positive	-1.63	0.26	0.26	0.26	0.26	0.26	0.26	0.26	0.26	-0.223	1.000	0.000	0
235	moderate	1,063	0.65	strong-positive	-1.63	0.26	0.26	0.26	0.26	0.26	0.26	0.26	0.26	-0.223	1.000	0.000	0
236	moderate	1,063	0.65	negative	-1.63	0.26	0.26	0.26	0.26	0.26	0.26	0.26	0.26	-0.223	1.000	0.000	0
237	moderate	1,063	0.85	absent	-2.70	0.82	0.82	0.82	0.82	0.82	0.82	0.82	0.82	-0.223	1.000	0.000	0
238	moderate	1,063	0.85	moderate-positive	-2.70	0.82	0.82	0.82	0.82	0.82	0.82	0.82	0.82	-0.223	1.000	0.000	0
239	moderate	1,063	0.85	strong-positive	-2.70	0.82	0.82	0.82	0.82	0.82	0.82	0.82	0.82	-0.223	1.000	0.000	0
240	moderate	1,063	0.85	negative	-2.70	0.82	0.82	0.82	0.82	0.82	0.82	0.82	0.82	-0.223	1.000	0.000	0
241	moderate	17,000	0.75	absent	-2.08	0.49	0.49	0.49	0.49	0.49	0.49	0.49	0.49	-0.223	1.000	0.000	0
242	moderate	17,000	0.75	moderate-positive	-2.08	0.49	0.49	0.49	0.49	0.49	0.49	0.49	0.49	-0.223	1.000	0.000	0
243	moderate	17,000	0.75	strong-positive	-2.08	0.49	0.49	0.49	0.49	0.49	0.49	0.49	0.49	-0.223	1.000	0.000	0
244	moderate	17,000	0.75	negative	-2.08	0.49	0.49	0.49	0.49	0.49	0.49	0.49	0.49	-0.223	1.000	0.000	0
245	moderate	17,000	0.65	absent	-1.63	0.26	0.26	0.26	0.26	0.26	0.26	0.26	0.26	-0.223	1.000	0.000	0
246	moderate	17,000	0.65	moderate-positive	-1.63	0.26	0.26	0.26	0.26	0.26	0.26	0.26	0.26	-0.223	1.000	0.000	0
247	moderate	17,000	0.65	strong-positive	-1.63	0.26	0.26	0.26	0.26	0.26	0.26	0.26	0.26	-0.223	1.000	0.000	0
248	moderate	17,000	0.65	negative	-1.63	0.26	0.26	0.26	0.26	0.26	0.26	0.26	0.26	-0.223	1.000	0.000	0
249	moderate	17,000	0.85	absent	-2.70	0.82	0.82	0.82	0.82	0.82	0.82	0.82	0.82	-0.223	1.000	0.000	0
250	moderate	17,000	0.85	moderate-positive	-2.70	0.82	0.82	0.82	0.82	0.82	0.82	0.82	0.82	-0.223	1.000	0.000	0
251	moderate	17,000	0.85	strong-positive	-2.70	0.82	0.82	0.82	0.82	0.82	0.82	0.82	0.82	-0.223	1.000	0.000	0
252	moderate	17,000	0.85	negative	-2.70	0.82	0.82	0.82	0.82	0.82	0.82	0.82	0.82	-0.223	1.000	0.000	0
253	moderate	4,250	0.75	absent	-2.08	0.49	0.49	0.49	0.49	0.49	0.49	0.49	0.49	-0.290	0.947	0.000	0
254	moderate	4,250	0.75	moderate-positive	-2.08	0.49	0.49	0.49	0.49	0.49	0.49	0.49	0.49	-0.290	0.947	0.000	0
255	moderate	4,250	0.75	strong-positive	-2.08	0.49	0.49	0.49	0.49	0.49	0.49	0.49	0.49	-0.290	0.947	0.000	0
256	moderate	4,250	0.75	negative	-2.08	0.49	0.49	0.49	0.49	0.49	0.49	0.49	0.49	-0.290	0.947	0.000	0
257	moderate	4,250	0.65	absent	-1.63	0.26	0.26	0.26	0.26	0.26	0.26	0.26	0.26	-0.300	0.934	0.000	0
258	moderate	4,250	0.65	moderate-positive	-1.63	0.26	0.26	0.26	0.26	0.26	0.26	0.26	0.26	-0.300	0.934	0.000	0
259	moderate	4,250	0.65	strong-positive	-1.63	0.26	0.26	0.26	0.26	0.26	0.26	0.26	0.26	-0.300	0.934	0.000	0
260	moderate	4,250	0.65	negative	-1.63	0.26	0.26	0.26	0.26	0.26	0.26	0.26	0.26	-0.300	0.934	0.000	0
261	moderate	4,250	0.85	absent	-2.70	0.82	0.82	0.82	0.82	0.82	0.82	0.82	0.82	-0.280	0.930	0.000	0
262	moderate	4,250	0.85	moderate-positive	-2.70	0.82	0.82	0.82	0.82	0.82	0.82	0.82	0.82	-0.280	0.930	0.000	0
263	moderate	4,250	0.85	strong-positive	-2.70	0.82	0.82	0.82	0.82	0.82	0.82	0.82	0.82	-0.280	0.930	0.000	0
264	moderate	4,250	0.85	negative	-2.70	0.82	0.82	0.82	0.82	0.82	0.82	0.82	0.82	-0.280	0.930	0.000	0
265	moderate	1,063	0.75	absent	-2.08	0.49	0.49	0.49	0.49	0.49	0.49	0.49	0.49	-0.290	0.947	0.000	0
266	moderate	1,063	0.75	moderate-positive	-2.08	0.49	0.49	0.49	0.49	0.49	0.49	0.49	0.49	-0.290	0.947	0.000	0

267	moderate	1,063	0.75	strong-positive	-2.08	0.49	0.49	0.49	0.49	0.49	0.49	0.49	0.49	-0.290	0.947	0.000	0
268	moderate	1,063	0.75	negative	-2.08	0.49	0.49	0.49	0.49	0.49	0.49	0.49	0.49	-0.290	0.947	0.000	0
269	moderate	1,063	0.65	absent	-1.63	0.26	0.26	0.26	0.26	0.26	0.26	0.26	0.26	-0.300	0.934	0.000	0
270	moderate	1,063	0.65	moderate-positive	-1.63	0.26	0.26	0.26	0.26	0.26	0.26	0.26	0.26	-0.300	0.934	0.000	0
271	moderate	1,063	0.65	strong-positive	-1.63	0.26	0.26	0.26	0.26	0.26	0.26	0.26	0.26	-0.300	0.934	0.000	0
272	moderate	1,063	0.65	negative	-1.63	0.26	0.26	0.26	0.26	0.26	0.26	0.26	0.26	-0.300	0.934	0.000	0
273	moderate	1,063	0.85	absent	-2.70	0.82	0.82	0.82	0.82	0.82	0.82	0.82	0.82	-0.280	0.930	0.000	0
274	moderate	1,063	0.85	moderate-positive	-2.70	0.82	0.82	0.82	0.82	0.82	0.82	0.82	0.82	-0.280	0.930	0.000	0
275	moderate	1,063	0.85	strong-positive	-2.70	0.82	0.82	0.82	0.82	0.82	0.82	0.82	0.82	-0.280	0.930	0.000	0
276	moderate	1,063	0.85	negative	-2.70	0.82	0.82	0.82	0.82	0.82	0.82	0.82	0.82	-0.280	0.930	0.000	0
277	moderate	17,000	0.75	absent	-2.08	0.49	0.49	0.49	0.49	0.49	0.49	0.49	0.49	-0.290	0.947	0.000	0
278	moderate	17,000	0.75	moderate-positive	-2.08	0.49	0.49	0.49	0.49	0.49	0.49	0.49	0.49	-0.290	0.947	0.000	0
279	moderate	17,000	0.75	strong-positive	-2.08	0.49	0.49	0.49	0.49	0.49	0.49	0.49	0.49	-0.290	0.947	0.000	0
280	moderate	17,000	0.75	negative	-2.08	0.49	0.49	0.49	0.49	0.49	0.49	0.49	0.49	-0.290	0.947	0.000	0
281	moderate	17,000	0.65	absent	-1.63	0.26	0.26	0.26	0.26	0.26	0.26	0.26	0.26	-0.300	0.934	0.000	0
282	moderate	17,000	0.65	moderate-positive	-1.63	0.26	0.26	0.26	0.26	0.26	0.26	0.26	0.26	-0.300	0.934	0.000	0
283	moderate	17,000	0.65	strong-positive	-1.63	0.26	0.26	0.26	0.26	0.26	0.26	0.26	0.26	-0.300	0.934	0.000	0
284	moderate	17,000	0.65	negative	-1.63	0.26	0.26	0.26	0.26	0.26	0.26	0.26	0.26	-0.300	0.934	0.000	0
285	moderate	17,000	0.85	absent	-2.70	0.82	0.82	0.82	0.82	0.82	0.82	0.82	0.82	-0.280	0.930	0.000	0
286	moderate	17,000	0.85	moderate-positive	-2.70	0.82	0.82	0.82	0.82	0.82	0.82	0.82	0.82	-0.280	0.930	0.000	0
287	moderate	17,000	0.85	strong-positive	-2.70	0.82	0.82	0.82	0.82	0.82	0.82	0.82	0.82	-0.280	0.930	0.000	0
288	moderate	17,000	0.85	negative	-2.70	0.82	0.82	0.82	0.82	0.82	0.82	0.82	0.82	-0.280	0.930	0.000	0
289	moderate	4,250	0.75	absent	-2.08	0.49	0.49	0.49	0.49	0.49	0.49	0.49	0.49	-0.460	0.796	0.000	0
290	moderate	4,250	0.75	moderate-positive	-2.08	0.49	0.49	0.49	0.49	0.49	0.49	0.49	0.49	-0.460	0.796	0.000	0
291	moderate	4,250	0.75	strong-positive	-2.08	0.49	0.49	0.49	0.49	0.49	0.49	0.49	0.49	-0.460	0.796	0.000	0
292	moderate	4,250	0.75	negative	-2.08	0.49	0.49	0.49	0.49	0.49	0.49	0.49	0.49	-0.460	0.796	0.000	0
293	moderate	4,250	0.65	absent	-1.63	0.26	0.26	0.26	0.26	0.26	0.26	0.26	0.26	-0.500	0.776	0.000	0
294	moderate	4,250	0.65	moderate-positive	-1.63	0.26	0.26	0.26	0.26	0.26	0.26	0.26	0.26	-0.500	0.776	0.000	0
295	moderate	4,250	0.65	strong-positive	-1.63	0.26	0.26	0.26	0.26	0.26	0.26	0.26	0.26	-0.500	0.776	0.000	0
296	moderate	4,250	0.65	negative	-1.63	0.26	0.26	0.26	0.26	0.26	0.26	0.26	0.26	-0.500	0.776	0.000	0
297	moderate	4,250	0.85	absent	-2.70	0.82	0.82	0.82	0.82	0.82	0.82	0.82	0.82	-0.410	0.785	0.000	0
298	moderate	4,250	0.85	moderate-positive	-2.70	0.82	0.82	0.82	0.82	0.82	0.82	0.82	0.82	-0.410	0.785	0.000	0
299	moderate	4,250	0.85	strong-positive	-2.70	0.82	0.82	0.82	0.82	0.82	0.82	0.82	0.82	-0.410	0.785	0.000	0
300	moderate	4,250	0.85	negative	-2.70	0.82	0.82	0.82	0.82	0.82	0.82	0.82	0.82	-0.410	0.785	0.000	0
301	moderate	1,063	0.75	absent	-2.08	0.49	0.49	0.49	0.49	0.49	0.49	0.49	0.49	-0.460	0.796	0.000	0
302	moderate	1,063	0.75	moderate-positive	-2.08	0.49	0.49	0.49	0.49	0.49	0.49	0.49	0.49	-0.460	0.796	0.000	0
303	moderate	1,063	0.75	strong-positive	-2.08	0.49	0.49	0.49	0.49	0.49	0.49	0.49	0.49	-0.460	0.796	0.000	0
304	moderate	1,063	0.75	negative	-2.08	0.49	0.49	0.49	0.49	0.49	0.49	0.49	0.49	-0.460	0.796	0.000	0
305	moderate	1,063	0.65	absent	-1.63	0.26	0.26	0.26	0.26	0.26	0.26	0.26	0.26	-0.500	0.776	0.000	0
306	moderate	1,063	0.65	moderate-positive	-1.63	0.26	0.26	0.26	0.26	0.26	0.26	0.26	0.26	-0.500	0.776	0.000	0
307	moderate	1,063	0.65	strong-positive	-1.63	0.26	0.26	0.26	0.26	0.26	0.26	0.26	0.26	-0.500	0.776	0.000	0
308	moderate	1,063	0.65	negative	-1.63	0.26	0.26	0.26	0.26	0.26	0.26	0.26	0.26	-0.500	0.776	0.000	0
309	moderate	1,063	0.85	absent	-2.70	0.82	0.82	0.82	0.82	0.82	0.82	0.82	0.82	-0.410	0.785	0.000	0
310	moderate	1,063	0.85	moderate-positive	-2.70	0.82	0.82	0.82	0.82	0.82	0.82	0.82	0.82	-0.410	0.785	0.000	0
311	moderate	1,063	0.85	strong-positive	-2.70	0.82	0.82	0.82	0.82	0.82	0.82	0.82	0.82	-0.410	0.785	0.000	0

312	moderate	1,063	0.85	negative	-2.70	0.82	0.82	0.82	0.82	0.82	0.82	0.82	0.82	-0.410	0.785	0.000	0
313	moderate	17,000	0.75	absent	-2.08	0.49	0.49	0.49	0.49	0.49	0.49	0.49	0.49	-0.460	0.796	0.000	0
314	moderate	17,000	0.75	moderate-positive	-2.08	0.49	0.49	0.49	0.49	0.49	0.49	0.49	0.49	-0.460	0.796	0.000	0
315	moderate	17,000	0.75	strong-positive	-2.08	0.49	0.49	0.49	0.49	0.49	0.49	0.49	0.49	-0.460	0.796	0.000	0
316	moderate	17,000	0.75	negative	-2.08	0.49	0.49	0.49	0.49	0.49	0.49	0.49	0.49	-0.460	0.796	0.000	0
317	moderate	17,000	0.65	absent	-1.63	0.26	0.26	0.26	0.26	0.26	0.26	0.26	0.26	-0.500	0.776	0.000	0
318	moderate	17,000	0.65	moderate-positive	-1.63	0.26	0.26	0.26	0.26	0.26	0.26	0.26	0.26	-0.500	0.776	0.000	0
319	moderate	17,000	0.65	strong-positive	-1.63	0.26	0.26	0.26	0.26	0.26	0.26	0.26	0.26	-0.500	0.776	0.000	0
320	moderate	17,000	0.65	negative	-1.63	0.26	0.26	0.26	0.26	0.26	0.26	0.26	0.26	-0.500	0.776	0.000	0
321	moderate	17,000	0.85	absent	-2.70	0.82	0.82	0.82	0.82	0.82	0.82	0.82	0.82	-0.410	0.785	0.000	0
322	moderate	17,000	0.85	moderate-positive	-2.70	0.82	0.82	0.82	0.82	0.82	0.82	0.82	0.82	-0.410	0.785	0.000	0
323	moderate	17,000	0.85	strong-positive	-2.70	0.82	0.82	0.82	0.82	0.82	0.82	0.82	0.82	-0.410	0.785	0.000	0
324	moderate	17,000	0.85	negative	-2.70	0.82	0.82	0.82	0.82	0.82	0.82	0.82	0.82	-0.410	0.785	0.000	0
325	moderate	4,250	0.75	absent	-2.08	0.49	0.49	0.49	0.49	0.49	0.49	0.49	0.49	-5.020	1.000	-0.013	-5
326	moderate	4,250	0.75	moderate-positive	-2.08	0.49	0.49	0.49	0.49	0.49	0.49	0.49	0.49	-5.020	1.000	-0.013	-5
327	moderate	4,250	0.75	strong-positive	-2.08	0.49	0.49	0.49	0.49	0.49	0.49	0.49	0.49	-5.020	1.000	-0.013	-5
328	moderate	4,250	0.75	negative	-2.08	0.49	0.49	0.49	0.49	0.49	0.49	0.49	0.49	-5.020	1.000	-0.013	-5
329	moderate	4,250	0.65	absent	-1.63	0.26	0.26	0.26	0.26	0.26	0.26	0.26	0.26	-4.990	1.000	-0.016	-5
330	moderate	4,250	0.65	moderate-positive	-1.63	0.26	0.26	0.26	0.26	0.26	0.26	0.26	0.26	-4.990	1.000	-0.016	-5
331	moderate	4,250	0.65	strong-positive	-1.63	0.26	0.26	0.26	0.26	0.26	0.26	0.26	0.26	-4.990	1.000	-0.016	-5
332	moderate	4,250	0.65	negative	-1.63	0.26	0.26	0.26	0.26	0.26	0.26	0.26	0.26	-4.990	1.000	-0.016	-5
333	moderate	4,250	0.85	absent	-2.70	0.82	0.82	0.82	0.82	0.82	0.82	0.82	0.82	-4.910	1.000	-0.016	-5
334	moderate	4,250	0.85	moderate-positive	-2.70	0.82	0.82	0.82	0.82	0.82	0.82	0.82	0.82	-4.910	1.000	-0.016	-5
335	moderate	4,250	0.85	strong-positive	-2.70	0.82	0.82	0.82	0.82	0.82	0.82	0.82	0.82	-4.910	1.000	-0.016	-5
336	moderate	4,250	0.85	negative	-2.70	0.82	0.82	0.82	0.82	0.82	0.82	0.82	0.82	-4.910	1.000	-0.016	-5
337	moderate	1,063	0.75	absent	-2.08	0.49	0.49	0.49	0.49	0.49	0.49	0.49	0.49	-5.020	1.000	-0.013	-5
338	moderate	1,063	0.75	moderate-positive	-2.08	0.49	0.49	0.49	0.49	0.49	0.49	0.49	0.49	-5.020	1.000	-0.013	-5
339	moderate	1,063	0.75	strong-positive	-2.08	0.49	0.49	0.49	0.49	0.49	0.49	0.49	0.49	-5.020	1.000	-0.013	-5
340	moderate	1,063	0.75	negative	-2.08	0.49	0.49	0.49	0.49	0.49	0.49	0.49	0.49	-5.020	1.000	-0.013	-5
341	moderate	1,063	0.65	absent	-1.63	0.26	0.26	0.26	0.26	0.26	0.26	0.26	0.26	-4.990	1.000	-0.016	-5
342	moderate	1,063	0.65	moderate-positive	-1.63	0.26	0.26	0.26	0.26	0.26	0.26	0.26	0.26	-4.990	1.000	-0.016	-5
343	moderate	1,063	0.65	strong-positive	-1.63	0.26	0.26	0.26	0.26	0.26	0.26	0.26	0.26	-4.990	1.000	-0.016	-5
344	moderate	1,063	0.65	negative	-1.63	0.26	0.26	0.26	0.26	0.26	0.26	0.26	0.26	-4.990	1.000	-0.016	-5
345	moderate	1,063	0.85	absent	-2.70	0.82	0.82	0.82	0.82	0.82	0.82	0.82	0.82	-4.910	1.000	-0.016	-5
346	moderate	1,063	0.85	moderate-positive	-2.70	0.82	0.82	0.82	0.82	0.82	0.82	0.82	0.82	-4.910	1.000	-0.016	-5
347	moderate	1,063	0.85	strong-positive	-2.70	0.82	0.82	0.82	0.82	0.82	0.82	0.82	0.82	-4.910	1.000	-0.016	-5
348	moderate	1,063	0.85	negative	-2.70	0.82	0.82	0.82	0.82	0.82	0.82	0.82	0.82	-4.910	1.000	-0.016	-5
349	moderate	17,000	0.75	absent	-2.08	0.49	0.49	0.49	0.49	0.49	0.49	0.49	0.49	-5.020	1.000	-0.013	-5
350	moderate	17,000	0.75	moderate-positive	-2.08	0.49	0.49	0.49	0.49	0.49	0.49	0.49	0.49	-5.020	1.000	-0.013	-5
351	moderate	17,000	0.75	strong-positive	-2.08	0.49	0.49	0.49	0.49	0.49	0.49	0.49	0.49	-5.020	1.000	-0.013	-5
352	moderate	17,000	0.75	negative	-2.08	0.49	0.49	0.49	0.49	0.49	0.49	0.49	0.49	-5.020	1.000	-0.013	-5
353	moderate	17,000	0.65	absent	-1.63	0.26	0.26	0.26	0.26	0.26	0.26	0.26	0.26	-4.990	1.000	-0.016	-5
354	moderate	17,000	0.65	moderate-positive	-1.63	0.26	0.26	0.26	0.26	0.26	0.26	0.26	0.26	-4.990	1.000	-0.016	-5
355	moderate	17,000	0.65	strong-positive	-1.63	0.26	0.26	0.26	0.26	0.26	0.26	0.26	0.26	-4.990	1.000	-0.016	-5
356	moderate	17,000	0.65	negative	-1.63	0.26	0.26	0.26	0.26	0.26	0.26	0.26	0.26	-4.990	1.000	-0.016	-5

357	moderate	17,000	0.85	absent	-2.70	0.82	0.82	0.82	0.82	0.82	0.82	0.82	0.82	-4.910	1.000	-0.016	-5
358	moderate	17,000	0.85	moderate-positive	-2.70	0.82	0.82	0.82	0.82	0.82	0.82	0.82	0.82	-4.910	1.000	-0.016	-5
359	moderate	17,000	0.85	strong-positive	-2.70	0.82	0.82	0.82	0.82	0.82	0.82	0.82	0.82	-4.910	1.000	-0.016	-5
360	moderate	17,000	0.85	negative	-2.70	0.82	0.82	0.82	0.82	0.82	0.82	0.82	0.82	-4.910	1.000	-0.016	-5
361	moderate	4,250	0.75	absent	-2.08	0.49	0.49	0.49	0.49	0.49	0.49	0.49	0.49	-4.420	1.000	-0.052	-5
362	moderate	4,250	0.75	moderate-positive	-2.08	0.49	0.49	0.49	0.49	0.49	0.49	0.49	0.49	-4.420	1.000	-0.052	-5
363	moderate	4,250	0.75	strong-positive	-2.08	0.49	0.49	0.49	0.49	0.49	0.49	0.49	0.49	-4.420	1.000	-0.052	-5
364	moderate	4,250	0.75	negative	-2.08	0.49	0.49	0.49	0.49	0.49	0.49	0.49	0.49	-4.420	1.000	-0.052	-5
365	moderate	4,250	0.65	absent	-1.63	0.26	0.26	0.26	0.26	0.26	0.26	0.26	0.26	-4.380	1.000	-0.059	-5
366	moderate	4,250	0.65	moderate-positive	-1.63	0.26	0.26	0.26	0.26	0.26	0.26	0.26	0.26	-4.380	1.000	-0.059	-5
367	moderate	4,250	0.65	strong-positive	-1.63	0.26	0.26	0.26	0.26	0.26	0.26	0.26	0.26	-4.380	1.000	-0.059	-5
368	moderate	4,250	0.65	negative	-1.63	0.26	0.26	0.26	0.26	0.26	0.26	0.26	0.26	-4.380	1.000	-0.059	-5
369	moderate	4,250	0.85	absent	-2.70	0.82	0.82	0.82	0.82	0.82	0.82	0.82	0.82	-4.130	1.000	-0.059	-5
370	moderate	4,250	0.85	moderate-positive	-2.70	0.82	0.82	0.82	0.82	0.82	0.82	0.82	0.82	-4.130	1.000	-0.059	-5
371	moderate	4,250	0.85	strong-positive	-2.70	0.82	0.82	0.82	0.82	0.82	0.82	0.82	0.82	-4.130	1.000	-0.059	-5
372	moderate	4,250	0.85	negative	-2.70	0.82	0.82	0.82	0.82	0.82	0.82	0.82	0.82	-4.130	1.000	-0.059	-5
373	moderate	1,063	0.75	absent	-2.08	0.49	0.49	0.49	0.49	0.49	0.49	0.49	0.49	-4.420	1.000	-0.052	-5
374	moderate	1,063	0.75	moderate-positive	-2.08	0.49	0.49	0.49	0.49	0.49	0.49	0.49	0.49	-4.420	1.000	-0.052	-5
375	moderate	1,063	0.75	strong-positive	-2.08	0.49	0.49	0.49	0.49	0.49	0.49	0.49	0.49	-4.420	1.000	-0.052	-5
376	moderate	1,063	0.75	negative	-2.08	0.49	0.49	0.49	0.49	0.49	0.49	0.49	0.49	-4.420	1.000	-0.052	-5
377	moderate	1,063	0.65	absent	-1.63	0.26	0.26	0.26	0.26	0.26	0.26	0.26	0.26	-4.380	1.000	-0.059	-5
378	moderate	1,063	0.65	moderate-positive	-1.63	0.26	0.26	0.26	0.26	0.26	0.26	0.26	0.26	-4.380	1.000	-0.059	-5
379	moderate	1,063	0.65	strong-positive	-1.63	0.26	0.26	0.26	0.26	0.26	0.26	0.26	0.26	-4.380	1.000	-0.059	-5
380	moderate	1,063	0.65	negative	-1.63	0.26	0.26	0.26	0.26	0.26	0.26	0.26	0.26	-4.380	1.000	-0.059	-5
381	moderate	1,063	0.85	absent	-2.70	0.82	0.82	0.82	0.82	0.82	0.82	0.82	0.82	-4.130	1.000	-0.059	-5
382	moderate	1,063	0.85	moderate-positive	-2.70	0.82	0.82	0.82	0.82	0.82	0.82	0.82	0.82	-4.130	1.000	-0.059	-5
383	moderate	1,063	0.85	strong-positive	-2.70	0.82	0.82	0.82	0.82	0.82	0.82	0.82	0.82	-4.130	1.000	-0.059	-5
384	moderate	1,063	0.85	negative	-2.70	0.82	0.82	0.82	0.82	0.82	0.82	0.82	0.82	-4.130	1.000	-0.059	-5
385	moderate	17,000	0.75	absent	-2.08	0.49	0.49	0.49	0.49	0.49	0.49	0.49	0.49	-4.420	1.000	-0.052	-5
386	moderate	17,000	0.75	moderate-positive	-2.08	0.49	0.49	0.49	0.49	0.49	0.49	0.49	0.49	-4.420	1.000	-0.052	-5
387	moderate	17,000	0.75	strong-positive	-2.08	0.49	0.49	0.49	0.49	0.49	0.49	0.49	0.49	-4.420	1.000	-0.052	-5
388	moderate	17,000	0.75	negative	-2.08	0.49	0.49	0.49	0.49	0.49	0.49	0.49	0.49	-4.420	1.000	-0.052	-5
389	moderate	17,000	0.65	absent	-1.63	0.26	0.26	0.26	0.26	0.26	0.26	0.26	0.26	-4.380	1.000	-0.059	-5
390	moderate	17,000	0.65	moderate-positive	-1.63	0.26	0.26	0.26	0.26	0.26	0.26	0.26	0.26	-4.380	1.000	-0.059	-5
391	moderate	17,000	0.65	strong-positive	-1.63	0.26	0.26	0.26	0.26	0.26	0.26	0.26	0.26	-4.380	1.000	-0.059	-5
392	moderate	17,000	0.65	negative	-1.63	0.26	0.26	0.26	0.26	0.26	0.26	0.26	0.26	-4.380	1.000	-0.059	-5
393	moderate	17,000	0.85	absent	-2.70	0.82	0.82	0.82	0.82	0.82	0.82	0.82	0.82	-4.130	1.000	-0.059	-5
394	moderate	17,000	0.85	moderate-positive	-2.70	0.82	0.82	0.82	0.82	0.82	0.82	0.82	0.82	-4.130	1.000	-0.059	-5
395	moderate	17,000	0.85	strong-positive	-2.70	0.82	0.82	0.82	0.82	0.82	0.82	0.82	0.82	-4.130	1.000	-0.059	-5
396	moderate	17,000	0.85	negative	-2.70	0.82	0.82	0.82	0.82	0.82	0.82	0.82	0.82	-4.130	1.000	-0.059	-5
397	moderate	4,250	0.75	absent	-2.08	0.49	0.49	0.49	0.49	0.49	0.49	0.49	0.49	0.173	1.560	0.105	0
398	moderate	4,250	0.75	moderate-positive	-2.08	0.49	0.49	0.49	0.49	0.49	0.49	0.49	0.49	0.173	1.560	0.105	0
399	moderate	4,250	0.75	strong-positive	-2.08	0.49	0.49	0.49	0.49	0.49	0.49	0.49	0.49	0.173	1.560	0.105	0
400	moderate	4,250	0.75	negative	-2.08	0.49	0.49	0.49	0.49	0.49	0.49	0.49	0.49	0.173	1.560	0.105	0
401	moderate	4,250	0.65	absent	-1.63	0.26	0.26	0.26	0.26	0.26	0.26	0.26	0.26	0.481	1.783	0.137	0

402	moderate	4,250	0.65	moderate-positive	-1.63	0.26	0.26	0.26	0.26	0.26	0.26	0.26	0.26	0.481	1.783	0.137	0
403	moderate	4,250	0.65	strong-positive	-1.63	0.26	0.26	0.26	0.26	0.26	0.26	0.26	0.26	0.481	1.783	0.137	0
404	moderate	4,250	0.65	negative	-1.63	0.26	0.26	0.26	0.26	0.26	0.26	0.26	0.26	0.481	1.783	0.137	0
405	moderate	4,250	0.85	absent	-2.70	0.82	0.82	0.82	0.82	0.82	0.82	0.82	0.82	-0.085	1.354	0.074	0
406	moderate	4,250	0.85	moderate-positive	-2.70	0.82	0.82	0.82	0.82	0.82	0.82	0.82	0.82	-0.085	1.354	0.074	0
407	moderate	4,250	0.85	strong-positive	-2.70	0.82	0.82	0.82	0.82	0.82	0.82	0.82	0.82	-0.085	1.354	0.074	0
408	moderate	4,250	0.85	negative	-2.70	0.82	0.82	0.82	0.82	0.82	0.82	0.82	0.82	-0.085	1.354	0.074	0
409	moderate	1,063	0.75	absent	-2.08	0.49	0.49	0.49	0.49	0.49	0.49	0.49	0.49	0.173	1.560	0.105	0
410	moderate	1,063	0.75	moderate-positive	-2.08	0.49	0.49	0.49	0.49	0.49	0.49	0.49	0.49	0.173	1.560	0.105	0
411	moderate	1,063	0.75	strong-positive	-2.08	0.49	0.49	0.49	0.49	0.49	0.49	0.49	0.49	0.173	1.560	0.105	0
412	moderate	1,063	0.75	negative	-2.08	0.49	0.49	0.49	0.49	0.49	0.49	0.49	0.49	0.173	1.560	0.105	0
413	moderate	1,063	0.65	absent	-1.63	0.26	0.26	0.26	0.26	0.26	0.26	0.26	0.26	0.481	1.783	0.137	0
414	moderate	1,063	0.65	moderate-positive	-1.63	0.26	0.26	0.26	0.26	0.26	0.26	0.26	0.26	0.481	1.783	0.137	0
415	moderate	1,063	0.65	strong-positive	-1.63	0.26	0.26	0.26	0.26	0.26	0.26	0.26	0.26	0.481	1.783	0.137	0
416	moderate	1,063	0.65	negative	-1.63	0.26	0.26	0.26	0.26	0.26	0.26	0.26	0.26	0.481	1.783	0.137	0
417	moderate	1,063	0.85	absent	-2.70	0.82	0.82	0.82	0.82	0.82	0.82	0.82	0.82	-0.085	1.354	0.074	0
418	moderate	1,063	0.85	moderate-positive	-2.70	0.82	0.82	0.82	0.82	0.82	0.82	0.82	0.82	-0.085	1.354	0.074	0
419	moderate	1,063	0.85	strong-positive	-2.70	0.82	0.82	0.82	0.82	0.82	0.82	0.82	0.82	-0.085	1.354	0.074	0
420	moderate	1,063	0.85	negative	-2.70	0.82	0.82	0.82	0.82	0.82	0.82	0.82	0.82	-0.085	1.354	0.074	0
421	moderate	17,000	0.75	absent	-2.08	0.49	0.49	0.49	0.49	0.49	0.49	0.49	0.49	0.173	1.560	0.105	0
422	moderate	17,000	0.75	moderate-positive	-2.08	0.49	0.49	0.49	0.49	0.49	0.49	0.49	0.49	0.173	1.560	0.105	0
423	moderate	17,000	0.75	strong-positive	-2.08	0.49	0.49	0.49	0.49	0.49	0.49	0.49	0.49	0.173	1.560	0.105	0
424	moderate	17,000	0.75	negative	-2.08	0.49	0.49	0.49	0.49	0.49	0.49	0.49	0.49	0.173	1.560	0.105	0
425	moderate	17,000	0.65	absent	-1.63	0.26	0.26	0.26	0.26	0.26	0.26	0.26	0.26	0.481	1.783	0.137	0
426	moderate	17,000	0.65	moderate-positive	-1.63	0.26	0.26	0.26	0.26	0.26	0.26	0.26	0.26	0.481	1.783	0.137	0
427	moderate	17,000	0.65	strong-positive	-1.63	0.26	0.26	0.26	0.26	0.26	0.26	0.26	0.26	0.481	1.783	0.137	0
428	moderate	17,000	0.65	negative	-1.63	0.26	0.26	0.26	0.26	0.26	0.26	0.26	0.26	0.481	1.783	0.137	0
429	moderate	17,000	0.85	absent	-2.70	0.82	0.82	0.82	0.82	0.82	0.82	0.82	0.82	-0.085	1.354	0.074	0
430	moderate	17,000	0.85	moderate-positive	-2.70	0.82	0.82	0.82	0.82	0.82	0.82	0.82	0.82	-0.085	1.354	0.074	0
431	moderate	17,000	0.85	strong-positive	-2.70	0.82	0.82	0.82	0.82	0.82	0.82	0.82	0.82	-0.085	1.354	0.074	0
432	moderate	17,000	0.85	negative	-2.70	0.82	0.82	0.82	0.82	0.82	0.82	0.82	0.82	-0.085	1.354	0.074	0
433	high	4,250	0.75	absent	-2.08	0.49	0.49	0.49	0.49	0.49	0.49	0.49	0.49	-0.693	1.000	0.000	0
434	high	4,250	0.75	moderate-positive	-2.08	0.49	0.49	0.49	0.49	0.49	0.49	0.49	0.49	-0.693	1.000	0.000	0
435	high	4,250	0.75	strong-positive	-2.08	0.49	0.49	0.49	0.49	0.49	0.49	0.49	0.49	-0.693	1.000	0.000	0
436	high	4,250	0.75	negative	-2.08	0.49	0.49	0.49	0.49	0.49	0.49	0.49	0.49	-0.693	1.000	0.000	0
437	high	4,250	0.65	absent	-1.63	0.26	0.26	0.26	0.26	0.26	0.26	0.26	0.26	-0.693	1.000	0.000	0
438	high	4,250	0.65	moderate-positive	-1.63	0.26	0.26	0.26	0.26	0.26	0.26	0.26	0.26	-0.693	1.000	0.000	0
439	high	4,250	0.65	strong-positive	-1.63	0.26	0.26	0.26	0.26	0.26	0.26	0.26	0.26	-0.693	1.000	0.000	0
440	high	4,250	0.65	negative	-1.63	0.26	0.26	0.26	0.26	0.26	0.26	0.26	0.26	-0.693	1.000	0.000	0
441	high	4,250	0.85	absent	-2.70	0.82	0.82	0.82	0.82	0.82	0.82	0.82	0.82	-0.693	1.000	0.000	0
442	high	4,250	0.85	moderate-positive	-2.70	0.82	0.82	0.82	0.82	0.82	0.82	0.82	0.82	-0.693	1.000	0.000	0
443	high	4,250	0.85	strong-positive	-2.70	0.82	0.82	0.82	0.82	0.82	0.82	0.82	0.82	-0.693	1.000	0.000	0
444	high	4,250	0.85	negative	-2.70	0.82	0.82	0.82	0.82	0.82	0.82	0.82	0.82	-0.693	1.000	0.000	0
445	high	1,063	0.75	absent	-2.08	0.49	0.49	0.49	0.49	0.49	0.49	0.49	0.49	-0.693	1.000	0.000	0
446	high	1,063	0.75	moderate-positive	-2.08	0.49	0.49	0.49	0.49	0.49	0.49	0.49	0.49	-0.693	1.000	0.000	0

447	high	1,063	0.75	strong-positive	-2.08	0.49	0.49	0.49	0.49	0.49	0.49	0.49	0.49	-0.693	1.000	0.000	0
448	high	1,063	0.75	negative	-2.08	0.49	0.49	0.49	0.49	0.49	0.49	0.49	0.49	-0.693	1.000	0.000	0
449	high	1,063	0.65	absent	-1.63	0.26	0.26	0.26	0.26	0.26	0.26	0.26	0.26	-0.693	1.000	0.000	0
450	high	1,063	0.65	moderate-positive	-1.63	0.26	0.26	0.26	0.26	0.26	0.26	0.26	0.26	-0.693	1.000	0.000	0
451	high	1,063	0.65	strong-positive	-1.63	0.26	0.26	0.26	0.26	0.26	0.26	0.26	0.26	-0.693	1.000	0.000	0
452	high	1,063	0.65	negative	-1.63	0.26	0.26	0.26	0.26	0.26	0.26	0.26	0.26	-0.693	1.000	0.000	0
453	high	1,063	0.85	absent	-2.70	0.82	0.82	0.82	0.82	0.82	0.82	0.82	0.82	-0.693	1.000	0.000	0
454	high	1,063	0.85	moderate-positive	-2.70	0.82	0.82	0.82	0.82	0.82	0.82	0.82	0.82	-0.693	1.000	0.000	0
455	high	1,063	0.85	strong-positive	-2.70	0.82	0.82	0.82	0.82	0.82	0.82	0.82	0.82	-0.693	1.000	0.000	0
456	high	1,063	0.85	negative	-2.70	0.82	0.82	0.82	0.82	0.82	0.82	0.82	0.82	-0.693	1.000	0.000	0
457	high	17,000	0.75	absent	-2.08	0.49	0.49	0.49	0.49	0.49	0.49	0.49	0.49	-0.693	1.000	0.000	0
458	high	17,000	0.75	moderate-positive	-2.08	0.49	0.49	0.49	0.49	0.49	0.49	0.49	0.49	-0.693	1.000	0.000	0
459	high	17,000	0.75	strong-positive	-2.08	0.49	0.49	0.49	0.49	0.49	0.49	0.49	0.49	-0.693	1.000	0.000	0
460	high	17,000	0.75	negative	-2.08	0.49	0.49	0.49	0.49	0.49	0.49	0.49	0.49	-0.693	1.000	0.000	0
461	high	17,000	0.65	absent	-1.63	0.26	0.26	0.26	0.26	0.26	0.26	0.26	0.26	-0.693	1.000	0.000	0
462	high	17,000	0.65	moderate-positive	-1.63	0.26	0.26	0.26	0.26	0.26	0.26	0.26	0.26	-0.693	1.000	0.000	0
463	high	17,000	0.65	strong-positive	-1.63	0.26	0.26	0.26	0.26	0.26	0.26	0.26	0.26	-0.693	1.000	0.000	0
464	high	17,000	0.65	negative	-1.63	0.26	0.26	0.26	0.26	0.26	0.26	0.26	0.26	-0.693	1.000	0.000	0
465	high	17,000	0.85	absent	-2.70	0.82	0.82	0.82	0.82	0.82	0.82	0.82	0.82	-0.693	1.000	0.000	0
466	high	17,000	0.85	moderate-positive	-2.70	0.82	0.82	0.82	0.82	0.82	0.82	0.82	0.82	-0.693	1.000	0.000	0
467	high	17,000	0.85	strong-positive	-2.70	0.82	0.82	0.82	0.82	0.82	0.82	0.82	0.82	-0.693	1.000	0.000	0
468	high	17,000	0.85	negative	-2.70	0.82	0.82	0.82	0.82	0.82	0.82	0.82	0.82	-0.693	1.000	0.000	0
469	high	4,250	0.75	absent	-2.08	0.49	0.49	0.49	0.49	0.49	0.49	0.49	0.49	-0.750	0.947	0.000	0
470	high	4,250	0.75	moderate-positive	-2.08	0.49	0.49	0.49	0.49	0.49	0.49	0.49	0.49	-0.750	0.947	0.000	0
471	high	4,250	0.75	strong-positive	-2.08	0.49	0.49	0.49	0.49	0.49	0.49	0.49	0.49	-0.750	0.947	0.000	0
472	high	4,250	0.75	negative	-2.08	0.49	0.49	0.49	0.49	0.49	0.49	0.49	0.49	-0.750	0.947	0.000	0
473	high	4,250	0.65	absent	-1.63	0.26	0.26	0.26	0.26	0.26	0.26	0.26	0.26	-0.770	0.934	0.000	0
474	high	4,250	0.65	moderate-positive	-1.63	0.26	0.26	0.26	0.26	0.26	0.26	0.26	0.26	-0.770	0.934	0.000	0
475	high	4,250	0.65	strong-positive	-1.63	0.26	0.26	0.26	0.26	0.26	0.26	0.26	0.26	-0.770	0.934	0.000	0
476	high	4,250	0.65	negative	-1.63	0.26	0.26	0.26	0.26	0.26	0.26	0.26	0.26	-0.770	0.934	0.000	0
477	high	4,250	0.85	absent	-2.70	0.82	0.82	0.82	0.82	0.82	0.82	0.82	0.82	-0.730	0.930	0.000	0
478	high	4,250	0.85	moderate-positive	-2.70	0.82	0.82	0.82	0.82	0.82	0.82	0.82	0.82	-0.730	0.930	0.000	0
479	high	4,250	0.85	strong-positive	-2.70	0.82	0.82	0.82	0.82	0.82	0.82	0.82	0.82	-0.730	0.930	0.000	0
480	high	4,250	0.85	negative	-2.70	0.82	0.82	0.82	0.82	0.82	0.82	0.82	0.82	-0.730	0.930	0.000	0
481	high	1,063	0.75	absent	-2.08	0.49	0.49	0.49	0.49	0.49	0.49	0.49	0.49	-0.750	0.947	0.000	0
482	high	1,063	0.75	moderate-positive	-2.08	0.49	0.49	0.49	0.49	0.49	0.49	0.49	0.49	-0.750	0.947	0.000	0
483	high	1,063	0.75	strong-positive	-2.08	0.49	0.49	0.49	0.49	0.49	0.49	0.49	0.49	-0.750	0.947	0.000	0
484	high	1,063	0.75	negative	-2.08	0.49	0.49	0.49	0.49	0.49	0.49	0.49	0.49	-0.750	0.947	0.000	0
485	high	1,063	0.65	absent	-1.63	0.26	0.26	0.26	0.26	0.26	0.26	0.26	0.26	-0.770	0.934	0.000	0
486	high	1,063	0.65	moderate-positive	-1.63	0.26	0.26	0.26	0.26	0.26	0.26	0.26	0.26	-0.770	0.934	0.000	0
487	high	1,063	0.65	strong-positive	-1.63	0.26	0.26	0.26	0.26	0.26	0.26	0.26	0.26	-0.770	0.934	0.000	0
488	high	1,063	0.65	negative	-1.63	0.26	0.26	0.26	0.26	0.26	0.26	0.26	0.26	-0.770	0.934	0.000	0
489	high	1,063	0.85	absent	-2.70	0.82	0.82	0.82	0.82	0.82	0.82	0.82	0.82	-0.730	0.930	0.000	0
490	high	1,063	0.85	moderate-positive	-2.70	0.82	0.82	0.82	0.82	0.82	0.82	0.82	0.82	-0.730	0.930	0.000	0
491	high	1,063	0.85	strong-positive	-2.70	0.82	0.82	0.82	0.82	0.82	0.82	0.82	0.82	-0.730	0.930	0.000	0

492	high	1,063	0.85	negative	-2.70	0.82	0.82	0.82	0.82	0.82	0.82	0.82	0.82	-0.730	0.930	0.000	0
493	high	17,000	0.75	absent	-2.08	0.49	0.49	0.49	0.49	0.49	0.49	0.49	0.49	-0.750	0.947	0.000	0
494	high	17,000	0.75	moderate-positive	-2.08	0.49	0.49	0.49	0.49	0.49	0.49	0.49	0.49	-0.750	0.947	0.000	0
495	high	17,000	0.75	strong-positive	-2.08	0.49	0.49	0.49	0.49	0.49	0.49	0.49	0.49	-0.750	0.947	0.000	0
496	high	17,000	0.75	negative	-2.08	0.49	0.49	0.49	0.49	0.49	0.49	0.49	0.49	-0.750	0.947	0.000	0
497	high	17,000	0.65	absent	-1.63	0.26	0.26	0.26	0.26	0.26	0.26	0.26	0.26	-0.770	0.934	0.000	0
498	high	17,000	0.65	moderate-positive	-1.63	0.26	0.26	0.26	0.26	0.26	0.26	0.26	0.26	-0.770	0.934	0.000	0
499	high	17,000	0.65	strong-positive	-1.63	0.26	0.26	0.26	0.26	0.26	0.26	0.26	0.26	-0.770	0.934	0.000	0
500	high	17,000	0.65	negative	-1.63	0.26	0.26	0.26	0.26	0.26	0.26	0.26	0.26	-0.770	0.934	0.000	0
501	high	17,000	0.85	absent	-2.70	0.82	0.82	0.82	0.82	0.82	0.82	0.82	0.82	-0.730	0.930	0.000	0
502	high	17,000	0.85	moderate-positive	-2.70	0.82	0.82	0.82	0.82	0.82	0.82	0.82	0.82	-0.730	0.930	0.000	0
503	high	17,000	0.85	strong-positive	-2.70	0.82	0.82	0.82	0.82	0.82	0.82	0.82	0.82	-0.730	0.930	0.000	0
504	high	17,000	0.85	negative	-2.70	0.82	0.82	0.82	0.82	0.82	0.82	0.82	0.82	-0.730	0.930	0.000	0
505	high	4,250	0.75	absent	-2.08	0.49	0.49	0.49	0.49	0.49	0.49	0.49	0.49	-0.900	0.796	0.000	0
506	high	4,250	0.75	moderate-positive	-2.08	0.49	0.49	0.49	0.49	0.49	0.49	0.49	0.49	-0.900	0.796	0.000	0
507	high	4,250	0.75	strong-positive	-2.08	0.49	0.49	0.49	0.49	0.49	0.49	0.49	0.49	-0.900	0.796	0.000	0
508	high	4,250	0.75	negative	-2.08	0.49	0.49	0.49	0.49	0.49	0.49	0.49	0.49	-0.900	0.796	0.000	0
509	high	4,250	0.65	absent	-1.63	0.26	0.26	0.26	0.26	0.26	0.26	0.26	0.26	-0.960	0.776	0.000	0
510	high	4,250	0.65	moderate-positive	-1.63	0.26	0.26	0.26	0.26	0.26	0.26	0.26	0.26	-0.960	0.776	0.000	0
511	high	4,250	0.65	strong-positive	-1.63	0.26	0.26	0.26	0.26	0.26	0.26	0.26	0.26	-0.960	0.776	0.000	0
512	high	4,250	0.65	negative	-1.63	0.26	0.26	0.26	0.26	0.26	0.26	0.26	0.26	-0.960	0.776	0.000	0
513	high	4,250	0.85	absent	-2.70	0.82	0.82	0.82	0.82	0.82	0.82	0.82	0.82	-0.840	0.785	0.000	0
514	high	4,250	0.85	moderate-positive	-2.70	0.82	0.82	0.82	0.82	0.82	0.82	0.82	0.82	-0.840	0.785	0.000	0
515	high	4,250	0.85	strong-positive	-2.70	0.82	0.82	0.82	0.82	0.82	0.82	0.82	0.82	-0.840	0.785	0.000	0
516	high	4,250	0.85	negative	-2.70	0.82	0.82	0.82	0.82	0.82	0.82	0.82	0.82	-0.840	0.785	0.000	0
517	high	1,063	0.75	absent	-2.08	0.49	0.49	0.49	0.49	0.49	0.49	0.49	0.49	-0.900	0.796	0.000	0
518	high	1,063	0.75	moderate-positive	-2.08	0.49	0.49	0.49	0.49	0.49	0.49	0.49	0.49	-0.900	0.796	0.000	0
519	high	1,063	0.75	strong-positive	-2.08	0.49	0.49	0.49	0.49	0.49	0.49	0.49	0.49	-0.900	0.796	0.000	0
520	high	1,063	0.75	negative	-2.08	0.49	0.49	0.49	0.49	0.49	0.49	0.49	0.49	-0.900	0.796	0.000	0
521	high	1,063	0.65	absent	-1.63	0.26	0.26	0.26	0.26	0.26	0.26	0.26	0.26	-0.960	0.776	0.000	0
522	high	1,063	0.65	moderate-positive	-1.63	0.26	0.26	0.26	0.26	0.26	0.26	0.26	0.26	-0.960	0.776	0.000	0
523	high	1,063	0.65	strong-positive	-1.63	0.26	0.26	0.26	0.26	0.26	0.26	0.26	0.26	-0.960	0.776	0.000	0
524	high	1,063	0.65	negative	-1.63	0.26	0.26	0.26	0.26	0.26	0.26	0.26	0.26	-0.960	0.776	0.000	0
525	high	1,063	0.85	absent	-2.70	0.82	0.82	0.82	0.82	0.82	0.82	0.82	0.82	-0.840	0.785	0.000	0
526	high	1,063	0.85	moderate-positive	-2.70	0.82	0.82	0.82	0.82	0.82	0.82	0.82	0.82	-0.840	0.785	0.000	0
527	high	1,063	0.85	strong-positive	-2.70	0.82	0.82	0.82	0.82	0.82	0.82	0.82	0.82	-0.840	0.785	0.000	0
528	high	1,063	0.85	negative	-2.70	0.82	0.82	0.82	0.82	0.82	0.82	0.82	0.82	-0.840	0.785	0.000	0
529	high	17,000	0.75	absent	-2.08	0.49	0.49	0.49	0.49	0.49	0.49	0.49	0.49	-0.900	0.796	0.000	0
530	high	17,000	0.75	moderate-positive	-2.08	0.49	0.49	0.49	0.49	0.49	0.49	0.49	0.49	-0.900	0.796	0.000	0
531	high	17,000	0.75	strong-positive	-2.08	0.49	0.49	0.49	0.49	0.49	0.49	0.49	0.49	-0.900	0.796	0.000	0
532	high	17,000	0.75	negative	-2.08	0.49	0.49	0.49	0.49	0.49	0.49	0.49	0.49	-0.900	0.796	0.000	0
533	high	17,000	0.65	absent	-1.63	0.26	0.26	0.26	0.26	0.26	0.26	0.26	0.26	-0.960	0.776	0.000	0
534	high	17,000	0.65	moderate-positive	-1.63	0.26	0.26	0.26	0.26	0.26	0.26	0.26	0.26	-0.960	0.776	0.000	0
535	high	17,000	0.65	strong-positive	-1.63	0.26	0.26	0.26	0.26	0.26	0.26	0.26	0.26	-0.960	0.776	0.000	0
536	high	17,000	0.65	negative	-1.63	0.26	0.26	0.26	0.26	0.26	0.26	0.26	0.26	-0.960	0.776	0.000	0

537	high	17,000	0.85	absent	-2.70	0.82	0.82	0.82	0.82	0.82	0.82	0.82	0.82	-0.840	0.785	0.000	0
538	high	17,000	0.85	moderate-positive	-2.70	0.82	0.82	0.82	0.82	0.82	0.82	0.82	0.82	-0.840	0.785	0.000	0
539	high	17,000	0.85	strong-positive	-2.70	0.82	0.82	0.82	0.82	0.82	0.82	0.82	0.82	-0.840	0.785	0.000	0
540	high	17,000	0.85	negative	-2.70	0.82	0.82	0.82	0.82	0.82	0.82	0.82	0.82	-0.840	0.785	0.000	0
541	high	4,250	0.75	absent	-2.08	0.49	0.49	0.49	0.49	0.49	0.49	0.49	0.49	-5.480	1.000	-0.013	-5
542	high	4,250	0.75	moderate-positive	-2.08	0.49	0.49	0.49	0.49	0.49	0.49	0.49	0.49	-5.480	1.000	-0.013	-5
543	high	4,250	0.75	strong-positive	-2.08	0.49	0.49	0.49	0.49	0.49	0.49	0.49	0.49	-5.480	1.000	-0.013	-5
544	high	4,250	0.75	negative	-2.08	0.49	0.49	0.49	0.49	0.49	0.49	0.49	0.49	-5.480	1.000	-0.013	-5
545	high	4,250	0.65	absent	-1.63	0.26	0.26	0.26	0.26	0.26	0.26	0.26	0.26	-5.460	1.000	-0.016	-5
546	high	4,250	0.65	moderate-positive	-1.63	0.26	0.26	0.26	0.26	0.26	0.26	0.26	0.26	-5.460	1.000	-0.016	-5
547	high	4,250	0.65	strong-positive	-1.63	0.26	0.26	0.26	0.26	0.26	0.26	0.26	0.26	-5.460	1.000	-0.016	-5
548	high	4,250	0.65	negative	-1.63	0.26	0.26	0.26	0.26	0.26	0.26	0.26	0.26	-5.460	1.000	-0.016	-5
549	high	4,250	0.85	absent	-2.70	0.82	0.82	0.82	0.82	0.82	0.82	0.82	0.82	-5.350	1.000	-0.016	-5
550	high	4,250	0.85	moderate-positive	-2.70	0.82	0.82	0.82	0.82	0.82	0.82	0.82	0.82	-5.350	1.000	-0.016	-5
551	high	4,250	0.85	strong-positive	-2.70	0.82	0.82	0.82	0.82	0.82	0.82	0.82	0.82	-5.350	1.000	-0.016	-5
552	high	4,250	0.85	negative	-2.70	0.82	0.82	0.82	0.82	0.82	0.82	0.82	0.82	-5.350	1.000	-0.016	-5
553	high	1,063	0.75	absent	-2.08	0.49	0.49	0.49	0.49	0.49	0.49	0.49	0.49	-5.480	1.000	-0.013	-5
554	high	1,063	0.75	moderate-positive	-2.08	0.49	0.49	0.49	0.49	0.49	0.49	0.49	0.49	-5.480	1.000	-0.013	-5
555	high	1,063	0.75	strong-positive	-2.08	0.49	0.49	0.49	0.49	0.49	0.49	0.49	0.49	-5.480	1.000	-0.013	-5
556	high	1,063	0.75	negative	-2.08	0.49	0.49	0.49	0.49	0.49	0.49	0.49	0.49	-5.480	1.000	-0.013	-5
557	high	1,063	0.65	absent	-1.63	0.26	0.26	0.26	0.26	0.26	0.26	0.26	0.26	-5.460	1.000	-0.016	-5
558	high	1,063	0.65	moderate-positive	-1.63	0.26	0.26	0.26	0.26	0.26	0.26	0.26	0.26	-5.460	1.000	-0.016	-5
559	high	1,063	0.65	strong-positive	-1.63	0.26	0.26	0.26	0.26	0.26	0.26	0.26	0.26	-5.460	1.000	-0.016	-5
560	high	1,063	0.65	negative	-1.63	0.26	0.26	0.26	0.26	0.26	0.26	0.26	0.26	-5.460	1.000	-0.016	-5
561	high	1,063	0.85	absent	-2.70	0.82	0.82	0.82	0.82	0.82	0.82	0.82	0.82	-5.350	1.000	-0.016	-5
562	high	1,063	0.85	moderate-positive	-2.70	0.82	0.82	0.82	0.82	0.82	0.82	0.82	0.82	-5.350	1.000	-0.016	-5
563	high	1,063	0.85	strong-positive	-2.70	0.82	0.82	0.82	0.82	0.82	0.82	0.82	0.82	-5.350	1.000	-0.016	-5
564	high	1,063	0.85	negative	-2.70	0.82	0.82	0.82	0.82	0.82	0.82	0.82	0.82	-5.350	1.000	-0.016	-5
565	high	17,000	0.75	absent	-2.08	0.49	0.49	0.49	0.49	0.49	0.49	0.49	0.49	-5.480	1.000	-0.013	-5
566	high	17,000	0.75	moderate-positive	-2.08	0.49	0.49	0.49	0.49	0.49	0.49	0.49	0.49	-5.480	1.000	-0.013	-5
567	high	17,000	0.75	strong-positive	-2.08	0.49	0.49	0.49	0.49	0.49	0.49	0.49	0.49	-5.480	1.000	-0.013	-5
568	high	17,000	0.75	negative	-2.08	0.49	0.49	0.49	0.49	0.49	0.49	0.49	0.49	-5.480	1.000	-0.013	-5
569	high	17,000	0.65	absent	-1.63	0.26	0.26	0.26	0.26	0.26	0.26	0.26	0.26	-5.460	1.000	-0.016	-5
570	high	17,000	0.65	moderate-positive	-1.63	0.26	0.26	0.26	0.26	0.26	0.26	0.26	0.26	-5.460	1.000	-0.016	-5
571	high	17,000	0.65	strong-positive	-1.63	0.26	0.26	0.26	0.26	0.26	0.26	0.26	0.26	-5.460	1.000	-0.016	-5
572	high	17,000	0.65	negative	-1.63	0.26	0.26	0.26	0.26	0.26	0.26	0.26	0.26	-5.460	1.000	-0.016	-5
573	high	17,000	0.85	absent	-2.70	0.82	0.82	0.82	0.82	0.82	0.82	0.82	0.82	-5.350	1.000	-0.016	-5
574	high	17,000	0.85	moderate-positive	-2.70	0.82	0.82	0.82	0.82	0.82	0.82	0.82	0.82	-5.350	1.000	-0.016	-5
575	high	17,000	0.85	strong-positive	-2.70	0.82	0.82	0.82	0.82	0.82	0.82	0.82	0.82	-5.350	1.000	-0.016	-5
576	high	17,000	0.85	negative	-2.70	0.82	0.82	0.82	0.82	0.82	0.82	0.82	0.82	-5.350	1.000	-0.016	-5
577	high	4,250	0.75	absent	-2.08	0.49	0.49	0.49	0.49	0.49	0.49	0.49	0.49	-4.860	1.000	-0.052	-5
578	high	4,250	0.75	moderate-positive	-2.08	0.49	0.49	0.49	0.49	0.49	0.49	0.49	0.49	-4.860	1.000	-0.052	-5
579	high	4,250	0.75	strong-positive	-2.08	0.49	0.49	0.49	0.49	0.49	0.49	0.49	0.49	-4.860	1.000	-0.052	-5
580	high	4,250	0.75	negative	-2.08	0.49	0.49	0.49	0.49	0.49	0.49	0.49	0.49	-4.860	1.000	-0.052	-5
581	high	4,250	0.65	absent	-1.63	0.26	0.26	0.26	0.26	0.26	0.26	0.26	0.26	-4.840	1.000	-0.059	-5

582	high	4,250	0.65	moderate-positive	-1.63	0.26	0.26	0.26	0.26	0.26	0.26	0.26	0.26	-4.840	1.000	-0.059	-5
583	high	4,250	0.65	strong-positive	-1.63	0.26	0.26	0.26	0.26	0.26	0.26	0.26	0.26	-4.840	1.000	-0.059	-5
584	high	4,250	0.65	negative	-1.63	0.26	0.26	0.26	0.26	0.26	0.26	0.26	0.26	-4.840	1.000	-0.059	-5
585	high	4,250	0.85	absent	-2.70	0.82	0.82	0.82	0.82	0.82	0.82	0.82	0.82	-4.510	1.000	-0.059	-5
586	high	4,250	0.85	moderate-positive	-2.70	0.82	0.82	0.82	0.82	0.82	0.82	0.82	0.82	-4.510	1.000	-0.059	-5
587	high	4,250	0.85	strong-positive	-2.70	0.82	0.82	0.82	0.82	0.82	0.82	0.82	0.82	-4.510	1.000	-0.059	-5
588	high	4,250	0.85	negative	-2.70	0.82	0.82	0.82	0.82	0.82	0.82	0.82	0.82	-4.510	1.000	-0.059	-5
589	high	1,063	0.75	absent	-2.08	0.49	0.49	0.49	0.49	0.49	0.49	0.49	0.49	-4.860	1.000	-0.052	-5
590	high	1,063	0.75	moderate-positive	-2.08	0.49	0.49	0.49	0.49	0.49	0.49	0.49	0.49	-4.860	1.000	-0.052	-5
591	high	1,063	0.75	strong-positive	-2.08	0.49	0.49	0.49	0.49	0.49	0.49	0.49	0.49	-4.860	1.000	-0.052	-5
592	high	1,063	0.75	negative	-2.08	0.49	0.49	0.49	0.49	0.49	0.49	0.49	0.49	-4.860	1.000	-0.052	-5
593	high	1,063	0.65	absent	-1.63	0.26	0.26	0.26	0.26	0.26	0.26	0.26	0.26	-4.840	1.000	-0.059	-5
594	high	1,063	0.65	moderate-positive	-1.63	0.26	0.26	0.26	0.26	0.26	0.26	0.26	0.26	-4.840	1.000	-0.059	-5
595	high	1,063	0.65	strong-positive	-1.63	0.26	0.26	0.26	0.26	0.26	0.26	0.26	0.26	-4.840	1.000	-0.059	-5
596	high	1,063	0.65	negative	-1.63	0.26	0.26	0.26	0.26	0.26	0.26	0.26	0.26	-4.840	1.000	-0.059	-5
597	high	1,063	0.85	absent	-2.70	0.82	0.82	0.82	0.82	0.82	0.82	0.82	0.82	-4.510	1.000	-0.059	-5
598	high	1,063	0.85	moderate-positive	-2.70	0.82	0.82	0.82	0.82	0.82	0.82	0.82	0.82	-4.510	1.000	-0.059	-5
599	high	1,063	0.85	strong-positive	-2.70	0.82	0.82	0.82	0.82	0.82	0.82	0.82	0.82	-4.510	1.000	-0.059	-5
600	high	1,063	0.85	negative	-2.70	0.82	0.82	0.82	0.82	0.82	0.82	0.82	0.82	-4.510	1.000	-0.059	-5
601	high	17,000	0.75	absent	-2.08	0.49	0.49	0.49	0.49	0.49	0.49	0.49	0.49	-4.860	1.000	-0.052	-5
602	high	17,000	0.75	moderate-positive	-2.08	0.49	0.49	0.49	0.49	0.49	0.49	0.49	0.49	-4.860	1.000	-0.052	-5
603	high	17,000	0.75	strong-positive	-2.08	0.49	0.49	0.49	0.49	0.49	0.49	0.49	0.49	-4.860	1.000	-0.052	-5
604	high	17,000	0.75	negative	-2.08	0.49	0.49	0.49	0.49	0.49	0.49	0.49	0.49	-4.860	1.000	-0.052	-5
605	high	17,000	0.65	absent	-1.63	0.26	0.26	0.26	0.26	0.26	0.26	0.26	0.26	-4.840	1.000	-0.059	-5
606	high	17,000	0.65	moderate-positive	-1.63	0.26	0.26	0.26	0.26	0.26	0.26	0.26	0.26	-4.840	1.000	-0.059	-5
607	high	17,000	0.65	strong-positive	-1.63	0.26	0.26	0.26	0.26	0.26	0.26	0.26	0.26	-4.840	1.000	-0.059	-5
608	high	17,000	0.65	negative	-1.63	0.26	0.26	0.26	0.26	0.26	0.26	0.26	0.26	-4.840	1.000	-0.059	-5
609	high	17,000	0.85	absent	-2.70	0.82	0.82	0.82	0.82	0.82	0.82	0.82	0.82	-4.510	1.000	-0.059	-5
610	high	17,000	0.85	moderate-positive	-2.70	0.82	0.82	0.82	0.82	0.82	0.82	0.82	0.82	-4.510	1.000	-0.059	-5
611	high	17,000	0.85	strong-positive	-2.70	0.82	0.82	0.82	0.82	0.82	0.82	0.82	0.82	-4.510	1.000	-0.059	-5
612	high	17,000	0.85	negative	-2.70	0.82	0.82	0.82	0.82	0.82	0.82	0.82	0.82	-4.510	1.000	-0.059	-5
613	high	4,250	0.75	absent	-2.08	0.49	0.49	0.49	0.49	0.49	0.49	0.49	0.49	-0.084	2.035	0.210	0
614	high	4,250	0.75	moderate-positive	-2.08	0.49	0.49	0.49	0.49	0.49	0.49	0.49	0.49	-0.084	2.035	0.210	0
615	high	4,250	0.75	strong-positive	-2.08	0.49	0.49	0.49	0.49	0.49	0.49	0.49	0.49	-0.084	2.035	0.210	0
616	high	4,250	0.75	negative	-2.08	0.49	0.49	0.49	0.49	0.49	0.49	0.49	0.49	-0.084	2.035	0.210	0
617	high	4,250	0.65	absent	-1.63	0.26	0.26	0.26	0.26	0.26	0.26	0.26	0.26	0.786	2.762	0.321	0
618	high	4,250	0.65	moderate-positive	-1.63	0.26	0.26	0.26	0.26	0.26	0.26	0.26	0.26	0.786	2.762	0.321	0
619	high	4,250	0.65	strong-positive	-1.63	0.26	0.26	0.26	0.26	0.26	0.26	0.26	0.26	0.786	2.762	0.321	0
620	high	4,250	0.65	negative	-1.63	0.26	0.26	0.26	0.26	0.26	0.26	0.26	0.26	0.786	2.762	0.321	0
621	high	4,250	0.85	absent	-2.70	0.82	0.82	0.82	0.82	0.82	0.82	0.82	0.82	-0.621	1.566	0.138	0
622	high	4,250	0.85	moderate-positive	-2.70	0.82	0.82	0.82	0.82	0.82	0.82	0.82	0.82	-0.621	1.566	0.138	0
623	high	4,250	0.85	strong-positive	-2.70	0.82	0.82	0.82	0.82	0.82	0.82	0.82	0.82	-0.621	1.566	0.138	0
624	high	4,250	0.85	negative	-2.70	0.82	0.82	0.82	0.82	0.82	0.82	0.82	0.82	-0.621	1.566	0.138	0
625	high	1,063	0.75	absent	-2.08	0.49	0.49	0.49	0.49	0.49	0.49	0.49	0.49	-0.084	2.035	0.210	0
626	high	1,063	0.75	moderate-positive	-2.08	0.49	0.49	0.49	0.49	0.49	0.49	0.49	0.49	-0.084	2.035	0.210	0

627	high	1,063	0.75	strong-positive	-2.08	0.49	0.49	0.49	0.49	0.49	0.49	0.49	0.49	-0.084	2.035	0.210	0
628	high	1,063	0.75	negative	-2.08	0.49	0.49	0.49	0.49	0.49	0.49	0.49	0.49	-0.084	2.035	0.210	0
629	high	1,063	0.65	absent	-1.63	0.26	0.26	0.26	0.26	0.26	0.26	0.26	0.26	0.786	2.762	0.321	0
630	high	1,063	0.65	moderate-positive	-1.63	0.26	0.26	0.26	0.26	0.26	0.26	0.26	0.26	0.786	2.762	0.321	0
631	high	1,063	0.65	strong-positive	-1.63	0.26	0.26	0.26	0.26	0.26	0.26	0.26	0.26	0.786	2.762	0.321	0
632	high	1,063	0.65	negative	-1.63	0.26	0.26	0.26	0.26	0.26	0.26	0.26	0.26	0.786	2.762	0.321	0
633	high	1,063	0.85	absent	-2.70	0.82	0.82	0.82	0.82	0.82	0.82	0.82	0.82	-0.621	1.566	0.138	0
634	high	1,063	0.85	moderate-positive	-2.70	0.82	0.82	0.82	0.82	0.82	0.82	0.82	0.82	-0.621	1.566	0.138	0
635	high	1,063	0.85	strong-positive	-2.70	0.82	0.82	0.82	0.82	0.82	0.82	0.82	0.82	-0.621	1.566	0.138	0
636	high	1,063	0.85	negative	-2.70	0.82	0.82	0.82	0.82	0.82	0.82	0.82	0.82	-0.621	1.566	0.138	0
637	high	17,000	0.75	absent	-2.08	0.49	0.49	0.49	0.49	0.49	0.49	0.49	0.49	-0.084	2.035	0.210	0
638	high	17,000	0.75	moderate-positive	-2.08	0.49	0.49	0.49	0.49	0.49	0.49	0.49	0.49	-0.084	2.035	0.210	0
639	high	17,000	0.75	strong-positive	-2.08	0.49	0.49	0.49	0.49	0.49	0.49	0.49	0.49	-0.084	2.035	0.210	0
640	high	17,000	0.75	negative	-2.08	0.49	0.49	0.49	0.49	0.49	0.49	0.49	0.49	-0.084	2.035	0.210	0
641	high	17,000	0.65	absent	-1.63	0.26	0.26	0.26	0.26	0.26	0.26	0.26	0.26	0.786	2.762	0.321	0
642	high	17,000	0.65	moderate-positive	-1.63	0.26	0.26	0.26	0.26	0.26	0.26	0.26	0.26	0.786	2.762	0.321	0
643	high	17,000	0.65	strong-positive	-1.63	0.26	0.26	0.26	0.26	0.26	0.26	0.26	0.26	0.786	2.762	0.321	0
644	high	17,000	0.65	negative	-1.63	0.26	0.26	0.26	0.26	0.26	0.26	0.26	0.26	0.786	2.762	0.321	0
645	high	17,000	0.85	absent	-2.70	0.82	0.82	0.82	0.82	0.82	0.82	0.82	0.82	-0.621	1.566	0.138	0
646	high	17,000	0.85	moderate-positive	-2.70	0.82	0.82	0.82	0.82	0.82	0.82	0.82	0.82	-0.621	1.566	0.138	0
647	high	17,000	0.85	strong-positive	-2.70	0.82	0.82	0.82	0.82	0.82	0.82	0.82	0.82	-0.621	1.566	0.138	0
648	high	17,000	0.85	negative	-2.70	0.82	0.82	0.82	0.82	0.82	0.82	0.82	0.82	-0.621	1.566	0.138	0

3 Plausible scenario settings

In this section we present specific scenarios from our simulation settings in which evolution of benefit followed similar patterns to [1]. In this case patients were stratified into risk quarters based on their true baseline risk. Within each risk quarter we constructed boxplots of true benefit.

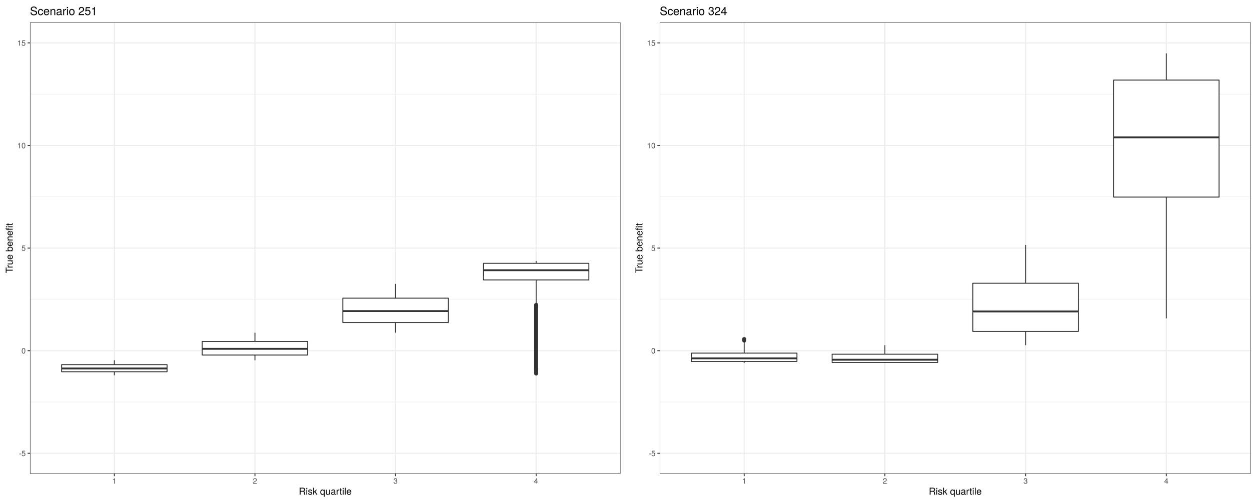

Figure S1: Simulation scenarios that closely follow trials. In this case, we see increasing absolute benefits with increasing baseline risk.

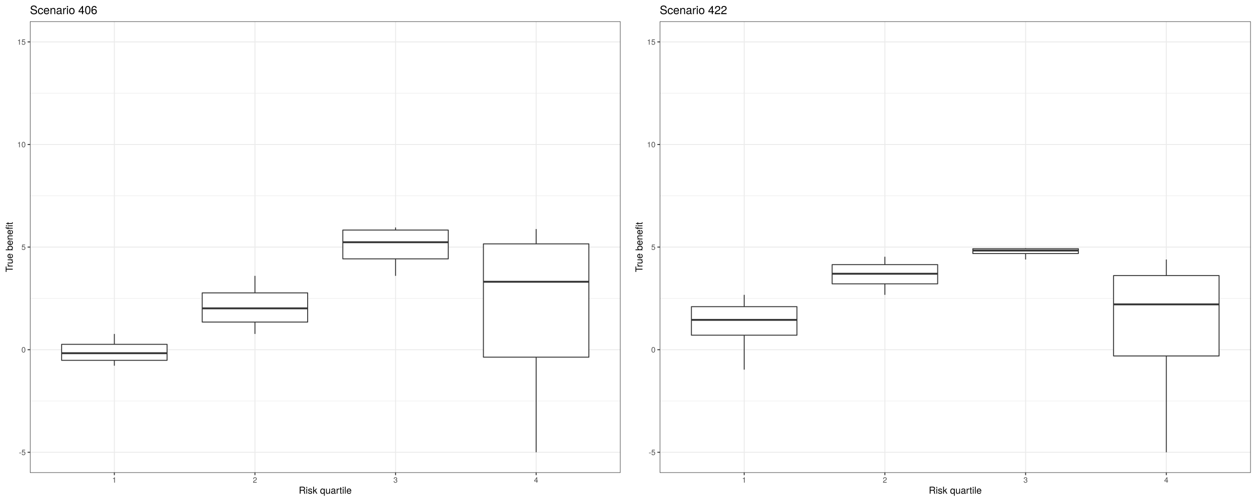

Figure S2: Simulation scenarios that closely follow trials. In this case, we see increasing absolute benefits with increasing baseline risk up to the third risk quarter. In the fourth risk quarter this trend is interrupted and benefits are diminished.

4 Approaches to individualize benefit predictions

4.1 Risk modeling

Merging treatment arms, we develop prediction models including a constant relative treatment effect:

$$P(Y = 1 \mid X = x, Z = z) = g(x^t\beta + \delta_1 z) \tag{3}$$

We derive baseline risk predictions for patients by setting $Z = 0$ in (4.1). All methods for individualizing benefit predictions are 2-stage methods, that start by fitting a model for predicting baseline risk. The estimated linear predictor of this model is

$$\hat{lp} = lp(x; \hat{\beta}) = x^t\hat{\beta}$$

4.2 Risk stratification

Derive a prediction model using the same approach as above and divide the population in equally sized risk-based subgroups. Estimate subgroup-specific absolute benefit from the observed absolute differences. Subject-specific benefit predictions are made by attributing to individuals their corresponding subgroup-specific estimate.

4.3 Constant treatment effect

Assuming a constant relative treatment effect, fit the adjusted model in (4.1). Then, predict absolute benefit using

$$\hat{\tau}(x; \hat{\beta}, \hat{\gamma}) = g(f(\hat{lp}, 0)) - g(f(\hat{lp}, 1)), \tag{4}$$

where $f(\hat{lp}, z) = \hat{lp} + \hat{\delta}_1 z$, with $\hat{\delta}_1$ the estimated relative treatment effect (log odds ratio).

4.4 Linear interaction

We relax the assumption of a constant relative treatment effect in (4) by setting

$$f(\hat{lp}, z) = \delta_0 + \delta_1 z + \delta_2 \hat{lp} + \delta_3 z \hat{lp}$$

4.5 Restricted cubic splines

Finally, we drop the linearity assumption and predict absolute benefit using smoothing with restricted cubic splines with $k = 3, 4$ and 5 knots. More specifically, we set:

$$f(\hat{lp}, z) = \delta_0 + \delta_1 z + zs(\hat{lp})$$

where

$$s(x) = \alpha_0 + \alpha_1 h_1(x) + \alpha_2 h_2(x) + \cdots + \alpha_{k-1} h_{k-1}(x)$$

with $h_1(x) = x$ and for $j = 2, \ldots, k-2$

$$h_{j+1}(x) = (x - t_j)^3 - (x - t_{k-1})^3_+ \frac{t_k - t_j}{t_k - t_{k-1}} + (x - t_k)^3_+ \frac{t_{k-1} - t_j}{t_k - t_{k-1}}$$

where $t_1, \ldots, t_k$ are the selected knots [2].

5 Adaptive model selection frequencies

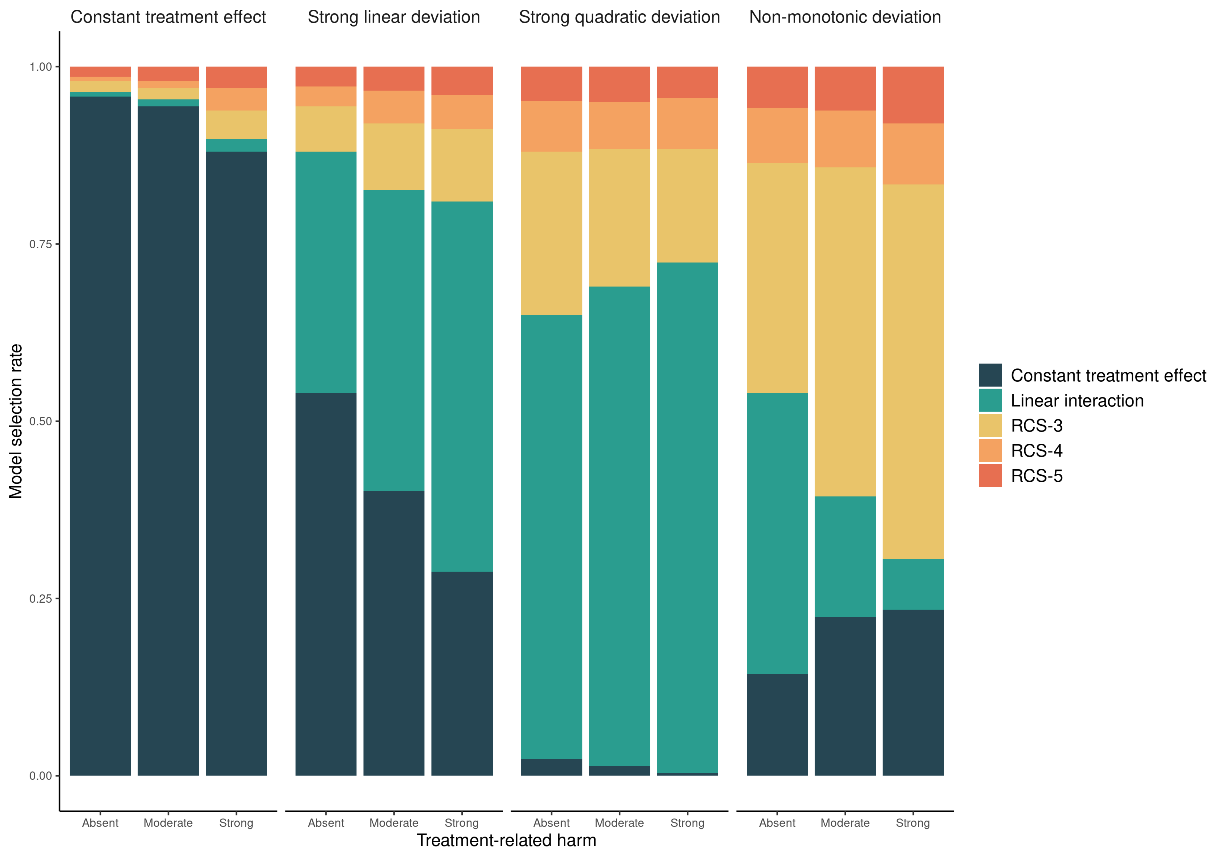

Figure S3: Model selection frequencies of the adaptive approach based on Akaike's Information Criterion across 500 replications. The scenario with the true constant relative treatment effect (first panel) had a true prediction AUC of 0.75 and sample size of 4,250.

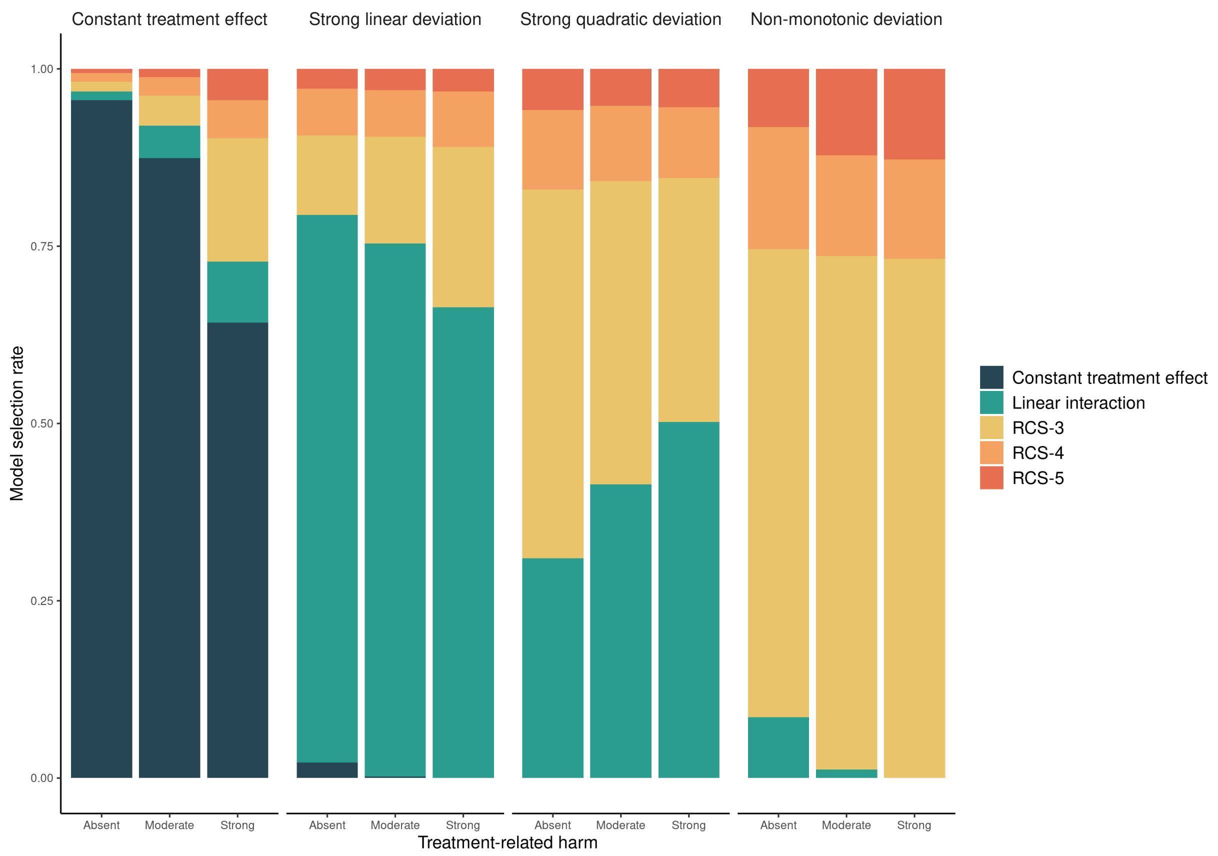

Figure S4: Model selection frequencies of the adaptive approach based on Akaike's Information Criterion across 500 replications. Sample size is 17,000 rather than 4,250 in Figure S3

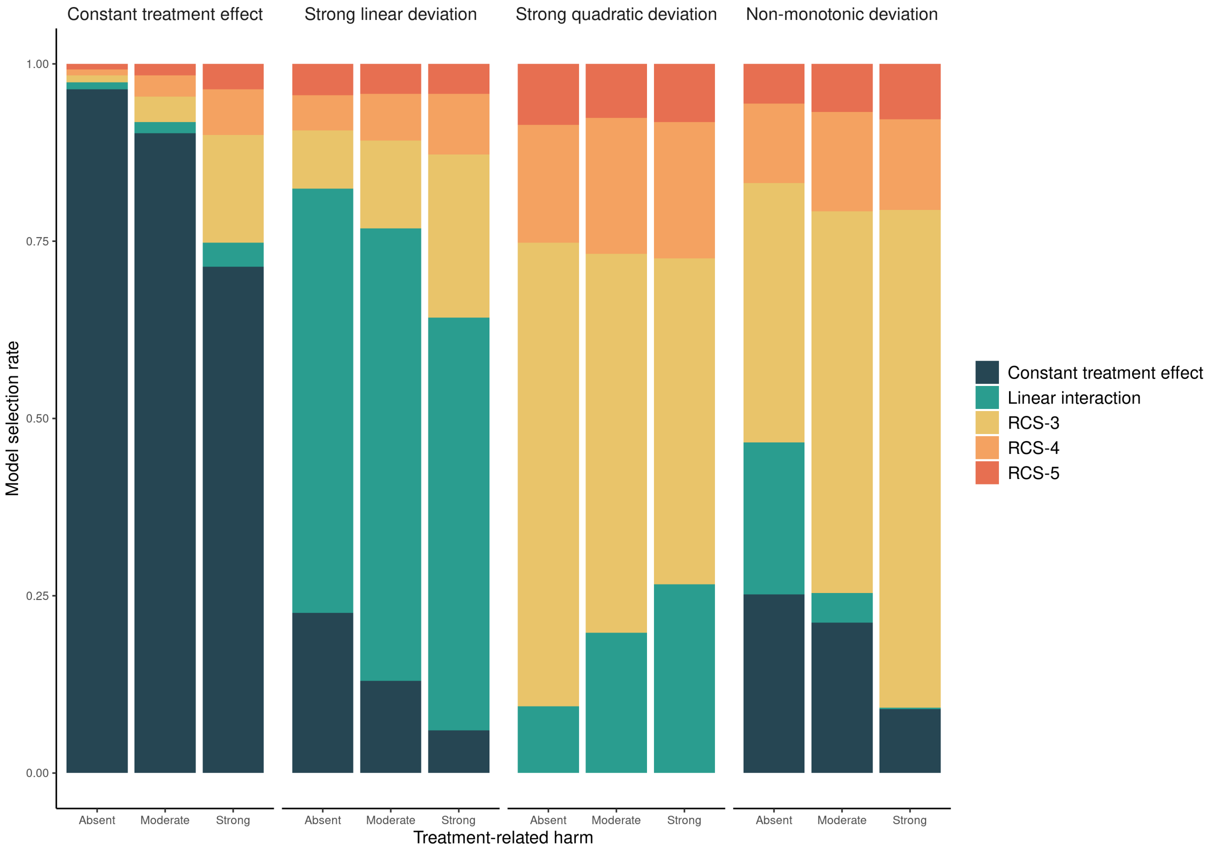

Figure S5: Model selection frequencies of the adaptive approach based on Akaike's Information Criterion across 500 replications. AUC is 0.85 rather than 0.75 in Figure S3

6 Discrimination and calibration for benefit

The c-for-benefit represents the probability that from two randomly chosen matched patient pairs with unequal observed benefit, the pair with greater observed benefit also has a higher predicted benefit. To be able to calculate observed benefit, patients in each treatment arm are ranked based on their predicted benefit and then matched 1:1 across treatment arms. Observed treatment benefit is defined as the difference of observed outcomes between the untreated and the treated patient of each matched patient pair. Predicted benefit is defined as the average of predicted benefit within each matched patient pair.

We evaluated calibration in a similar manner, using the integrated calibration index (ICI) for benefit [3]. The observed benefits are regressed on the predicted benefits using a locally weighted scatterplot smoother (loess). The ICI-for-benefit is the average absolute difference between predicted and smooth observed benefit. Values closer to represent better calibration.

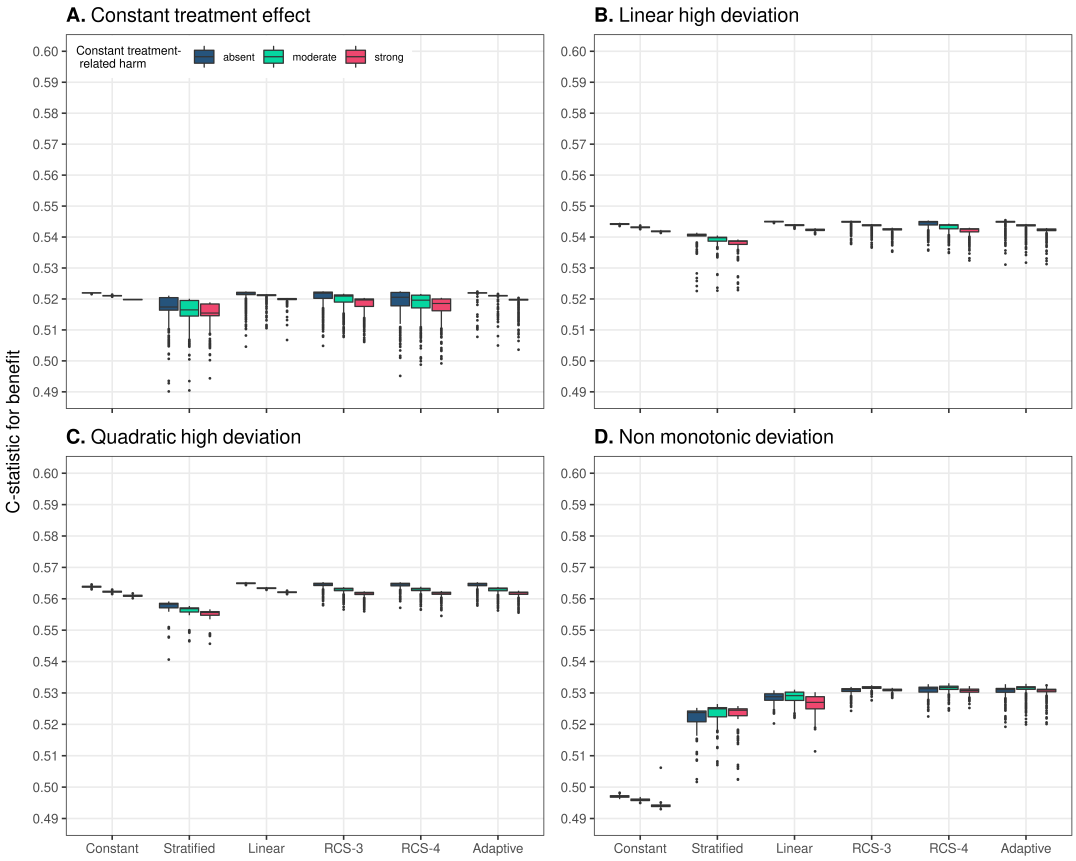

Figure S6: Discrimination for benefit of the considered methods across 500 replications calculated in a simulated sample of size 500,000. True prediction AUC of 0.75 and sample size of 17,000

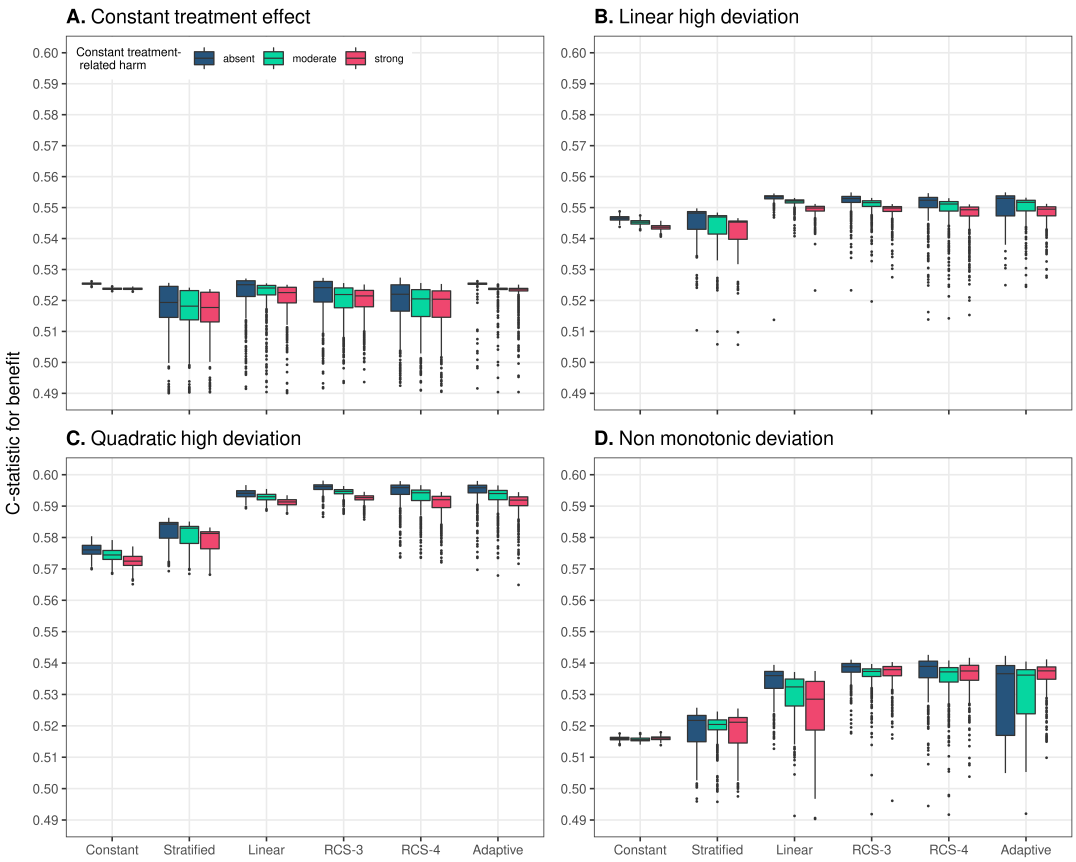

Figure S7: Discrimination for benefit of the considered methods across 500 replications calculated in a simulated sample of size 500,000. True prediction AUC of 0.85 and sample size of 4,250

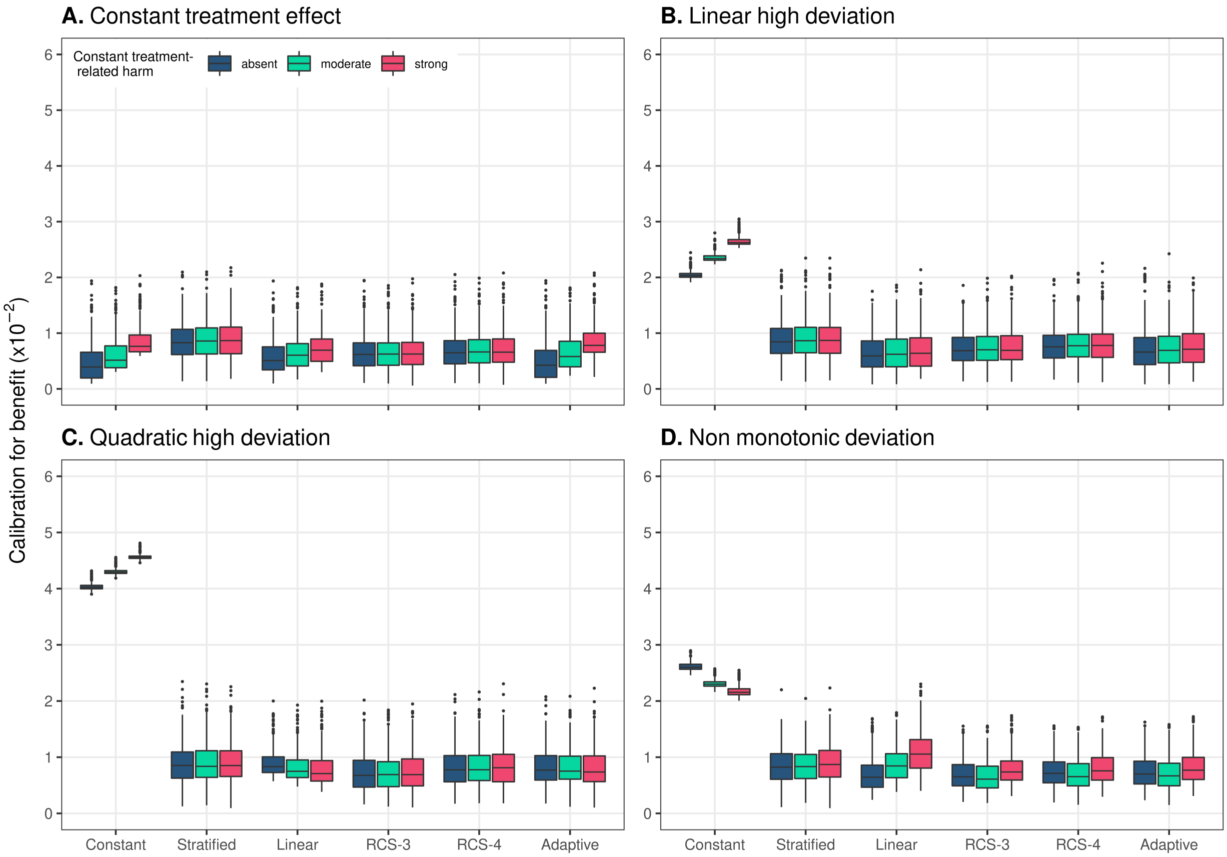

Figure S8: Calibration for benefit of the considered methods across 500 replications calculated in a simulated sample of size 500,000. True prediction AUC of 0.75 and sample size of 17,000

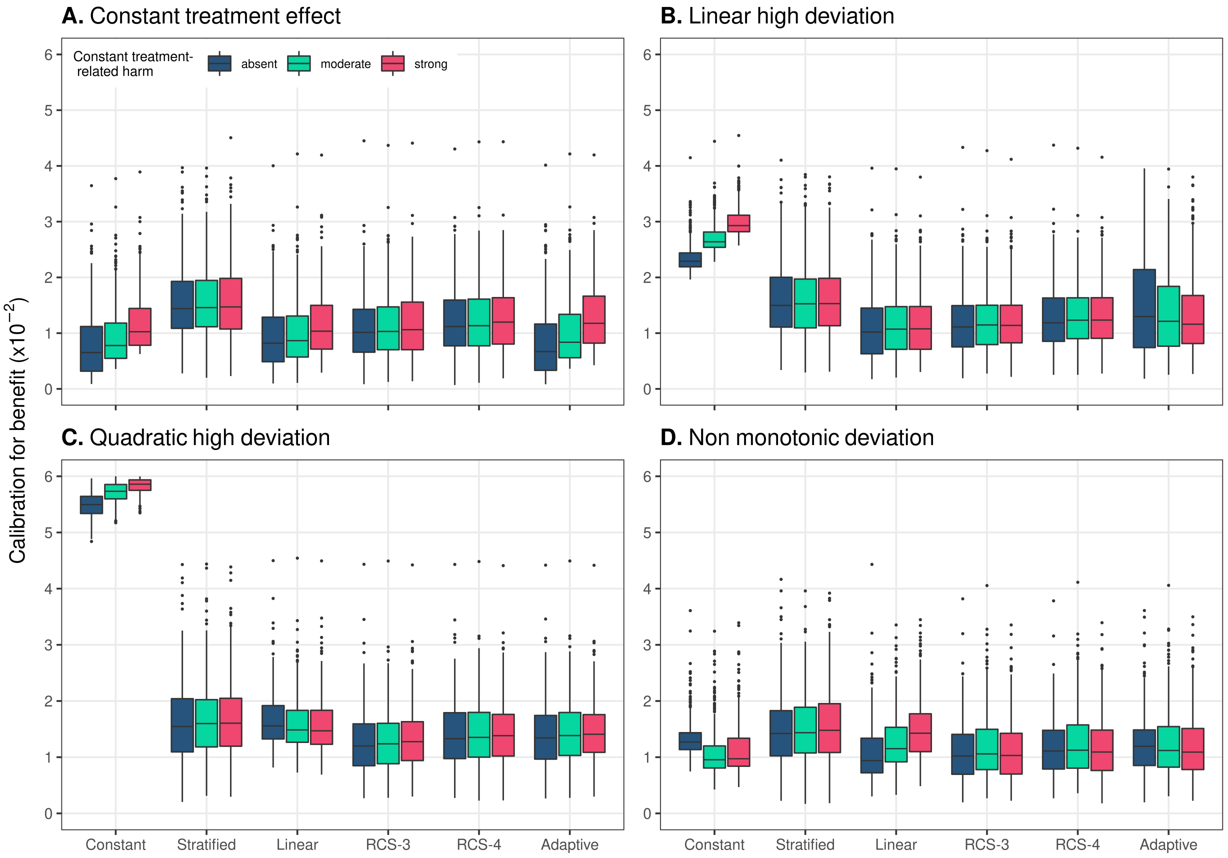

Figure S9: Calibration for benefit of the considered methods across 500 replications calculated in a simulated sample of size 500,000. True prediction AUC of 0.85 and sample size of 4,250

7 Strong relative treatment effect

Here we present the root mean squared error of the considered methods using strong constant relative treatment effect (OR = 0.5) as the reference. Again, the same sample size and prediction performance settings were considered along with the same settings for linear, quadratic and non-monotonic deviations from the base case scenario of constant relative treatment effects are considered. All results can be found at https://arekkas.shinyapps.io/simulation_viewer/.

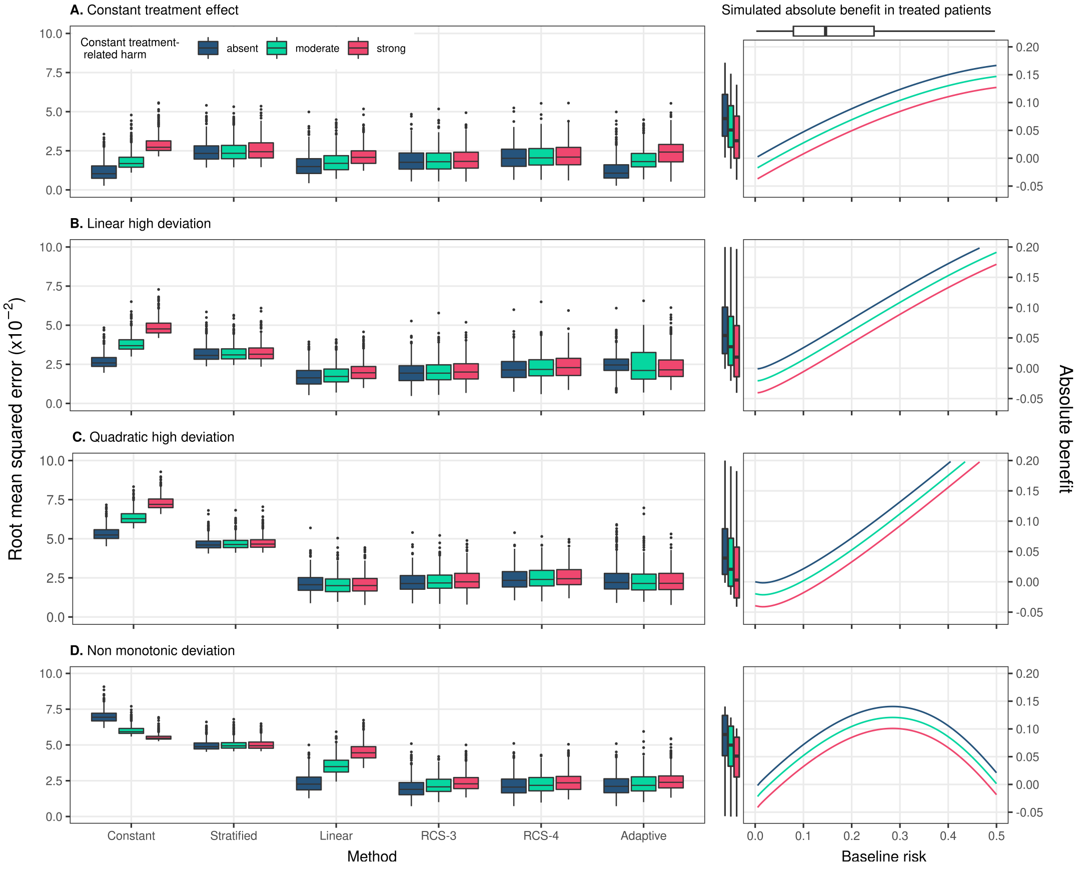

Figure S10: RMSE of the considered methods across 500 replications calculated in a simulated super-population of size 500,000. The scenario with true constant relative treatment effect (panel A) had a true prediction AUC of 0.75 and sample size of 4,250. The RMSE is also presented for strong linear (panel B), strong quadratic (panel C), and non-monotonic (panel D) deviations from constant relative treatment effects. Panels on the right side present the true relationship between baseline risk (x-axis) and absolute treatment benefit (y-axis). The 2.5, 25, 75 and 97.5 percentiles of the risk distribution are expressed in the boxplot.

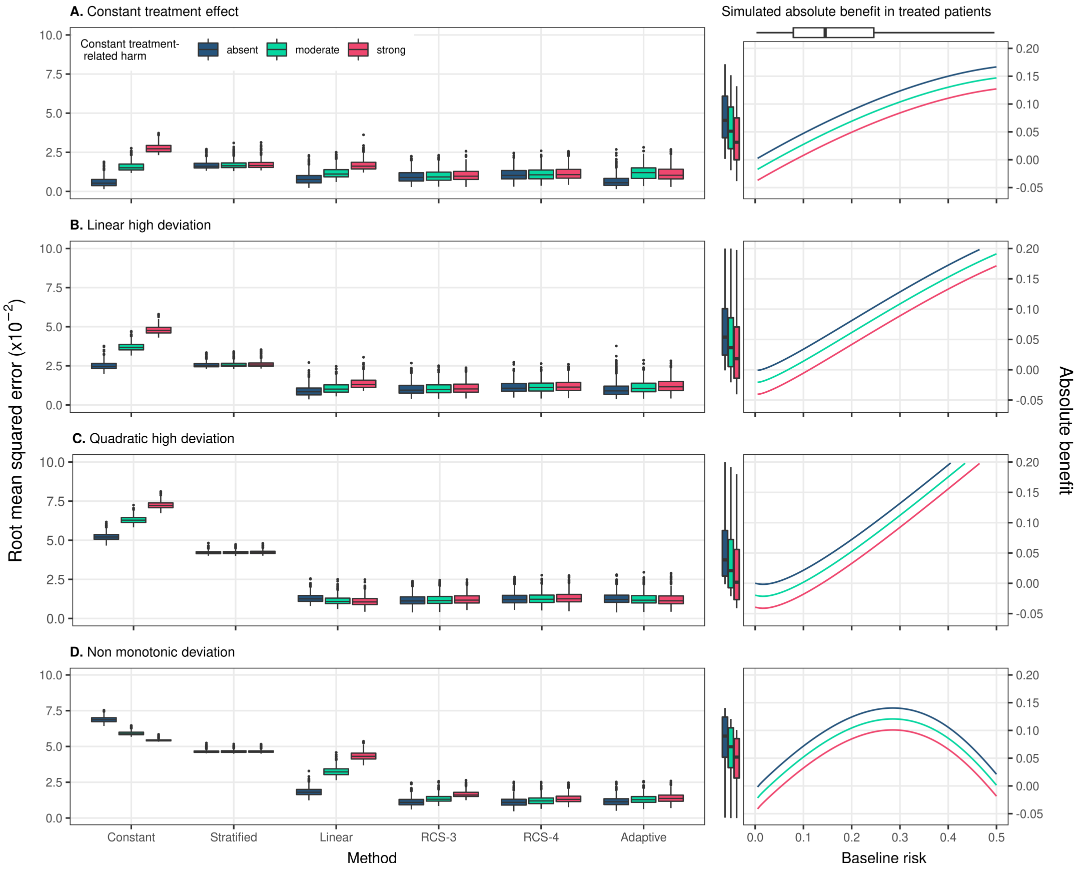

Figure S11: RMSE of the considered methods across 500 replcations calculated in a simulated sample of size 500,000. Sample size is 17,000 rather than 4,250 in Figure S10.

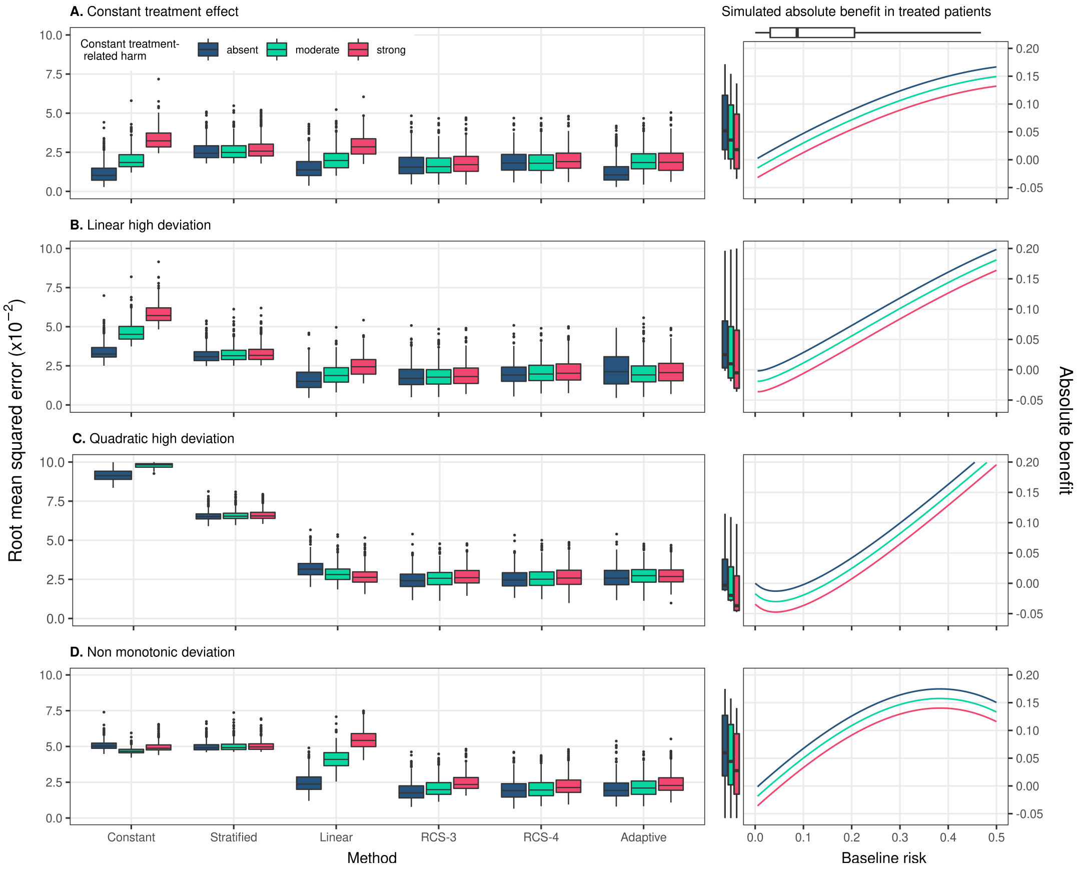

Figure S12: RMSE of the considered methods across 500 replcations calculated in a simulated sample of size 500,000. AUC is 0.85 rather than in Figure S10.

8 Treatment interactions

We carried out a smaller set of simulations, in which we assumed true treatment-covariate interactions. Sample size was set to 4,250 and the AUC of the true prediction model was set to 0.75. The following scenarios were considered: 1) 4 true weak positive interactions ($\text{OR}_{Z=1}/\text{OR}_{Z=0} = 0.83$); 2) 4 strong positive interactions ($\text{OR}_{Z=1}/\text{OR}_{Z=0} = 0.61$); 3) 2 weak and 2 strong positive interactions; 4) 4 weak negative interactions ($\text{OR}_{Z=1}/\text{OR}_{Z=0} = 1.17$); 5) 4 strong negative interactions ($\text{OR}_{Z=1}/\text{OR}_{Z=0} = 1.39$); 6) 2 weak and 2 strong negative interactions; 7) combined positive and negative strong interactions. We also considered constant treatment-related harms applied on the absolute scale to all treated patients. The exact settings were: 1) absent treatment-related harms; 2) moderate treatment-related harms, defined as 25% of the average true benefit of the scenario without treatment-related harms; 3) strong treatment-related harms defined as 50% of the true average benefit of the scenario without treatment-related harms; 4) negative treatment-related harms (benefit), defined as an absolute risk reduction for treated patients of 50% of the true average benefit of the scenario without treatment-related harms. The exact settings can be found in Table S2.

Table S2: Scenario settings of the treatment-covariate interaction scenarios.

Analysis ID						Baseline risk									True treatment effect				
Scenario	Base	Type	N	AUC	Treatment-related harm	b0	b1	b2	b3	b4	b5	b6	b7	b8	g0	g1	g2	g5	g6
649	interaction	weak	4,250	0.75	absent	-2.08	0.49	0.49	0.49	0.49	0.49	0.49	0.49	0.49	-0.69	-0.19	-0.19	-0.19	-0.19
650	interaction	weak	4,250	0.75	moderate-positive	-2.08	0.49	0.49	0.49	0.49	0.49	0.49	0.49	0.49	-0.69	-0.19	-0.19	-0.19	-0.19
651	interaction	weak	4,250	0.75	strong-positive	-2.08	0.49	0.49	0.49	0.49	0.49	0.49	0.49	0.49	-0.69	-0.19	-0.19	-0.19	-0.19
652	interaction	weak	4,250	0.75	negative	-2.08	0.49	0.49	0.49	0.49	0.49	0.49	0.49	0.49	-0.69	-0.19	-0.19	-0.19	-0.19
653	interaction	mixed	4,250	0.75	absent	-2.08	0.49	0.49	0.49	0.49	0.49	0.49	0.49	0.49	-0.69	-0.19	-0.49	-0.19	-0.49
654	interaction	mixed	4,250	0.75	moderate-positive	-2.08	0.49	0.49	0.49	0.49	0.49	0.49	0.49	0.49	-0.69	-0.19	-0.49	-0.19	-0.49
655	interaction	mixed	4,250	0.75	strong-positive	-2.08	0.49	0.49	0.49	0.49	0.49	0.49	0.49	0.49	-0.69	-0.19	-0.49	-0.19	-0.49
656	interaction	mixed	4,250	0.75	negative	-2.08	0.49	0.49	0.49	0.49	0.49	0.49	0.49	0.49	-0.69	-0.19	-0.49	-0.19	-0.49
657	interaction	strong	4,250	0.75	absent	-2.08	0.49	0.49	0.49	0.49	0.49	0.49	0.49	0.49	-0.69	-0.49	-0.49	-0.49	-0.49
658	interaction	strong	4,250	0.75	moderate-positive	-2.08	0.49	0.49	0.49	0.49	0.49	0.49	0.49	0.49	-0.69	-0.49	-0.49	-0.49	-0.49
659	interaction	strong	4,250	0.75	strong-positive	-2.08	0.49	0.49	0.49	0.49	0.49	0.49	0.49	0.49	-0.69	-0.49	-0.49	-0.49	-0.49
660	interaction	strong	4,250	0.75	negative	-2.08	0.49	0.49	0.49	0.49	0.49	0.49	0.49	0.49	-0.69	-0.49	-0.49	-0.49	-0.49
661	interaction	negative-weak	4,250	0.75	absent	-2.08	0.49	0.49	0.49	0.49	0.49	0.49	0.49	0.49	-0.69	0.16	0.16	0.16	0.16
662	interaction	negative-weak	4,250	0.75	moderate-positive	-2.08	0.49	0.49	0.49	0.49	0.49	0.49	0.49	0.49	-0.69	0.16	0.16	0.16	0.16
663	interaction	negative-weak	4,250	0.75	strong-positive	-2.08	0.49	0.49	0.49	0.49	0.49	0.49	0.49	0.49	-0.69	0.16	0.16	0.16	0.16
664	interaction	negative-weak	4,250	0.75	negative	-2.08	0.49	0.49	0.49	0.49	0.49	0.49	0.49	0.49	-0.69	0.16	0.16	0.16	0.16
665	interaction	negative-mixed	4,250	0.75	absent	-2.08	0.49	0.49	0.49	0.49	0.49	0.49	0.49	0.49	-0.69	0.16	0.33	0.16	0.33
666	interaction	negative-mixed	4,250	0.75	moderate-positive	-2.08	0.49	0.49	0.49	0.49	0.49	0.49	0.49	0.49	-0.69	0.16	0.33	0.16	0.33
667	interaction	negative-mixed	4,250	0.75	strong-positive	-2.08	0.49	0.49	0.49	0.49	0.49	0.49	0.49	0.49	-0.69	0.16	0.33	0.16	0.33
668	interaction	negative-mixed	4,250	0.75	negative	-2.08	0.49	0.49	0.49	0.49	0.49	0.49	0.49	0.49	-0.69	0.16	0.33	0.16	0.33
669	interaction	negative-strong	4,250	0.75	absent	-2.08	0.49	0.49	0.49	0.49	0.49	0.49	0.49	0.49	-0.69	0.33	0.33	0.33	0.33
670	interaction	negative-strong	4,250	0.75	moderate-positive	-2.08	0.49	0.49	0.49	0.49	0.49	0.49	0.49	0.49	-0.69	0.33	0.33	0.33	0.33
671	interaction	negative-strong	4,250	0.75	strong-positive	-2.08	0.49	0.49	0.49	0.49	0.49	0.49	0.49	0.49	-0.69	0.33	0.33	0.33	0.33
672	interaction	negative-strong	4,250	0.75	negative	-2.08	0.49	0.49	0.49	0.49	0.49	0.49	0.49	0.49	-0.69	0.33	0.33	0.33	0.33
673	interaction	combined	4,250	0.75	absent	-2.08	0.49	0.49	0.49	0.49	0.49	0.49	0.49	0.49	-0.69	-0.49	0.33	-0.49	0.33
674	interaction	combined	4,250	0.75	moderate-positive	-2.08	0.49	0.49	0.49	0.49	0.49	0.49	0.49	0.49	-0.69	-0.49	0.33	-0.49	0.33
675	interaction	combined	4,250	0.75	strong-positive	-2.08	0.49	0.49	0.49	0.49	0.49	0.49	0.49	0.49	-0.69	-0.49	0.33	-0.49	0.33
676	interaction	combined	4,250	0.75	negative	-2.08	0.49	0.49	0.49	0.49	0.49	0.49	0.49	0.49	-0.69	-0.49	0.33	-0.49	0.33

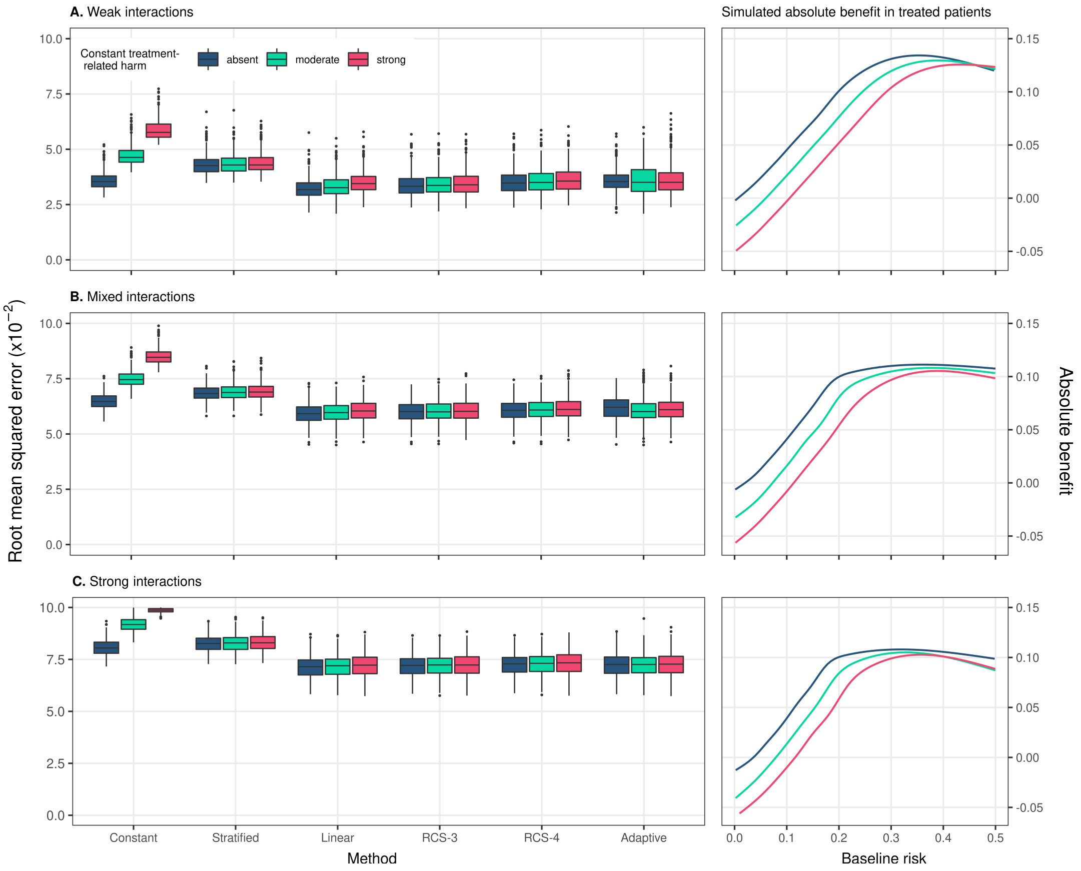

Figure S13: RMSE of the considered methods across 500 replications calculated in a simulated sample of size 500,000 where treatment-covariate interactions all favoring treatment were considered.

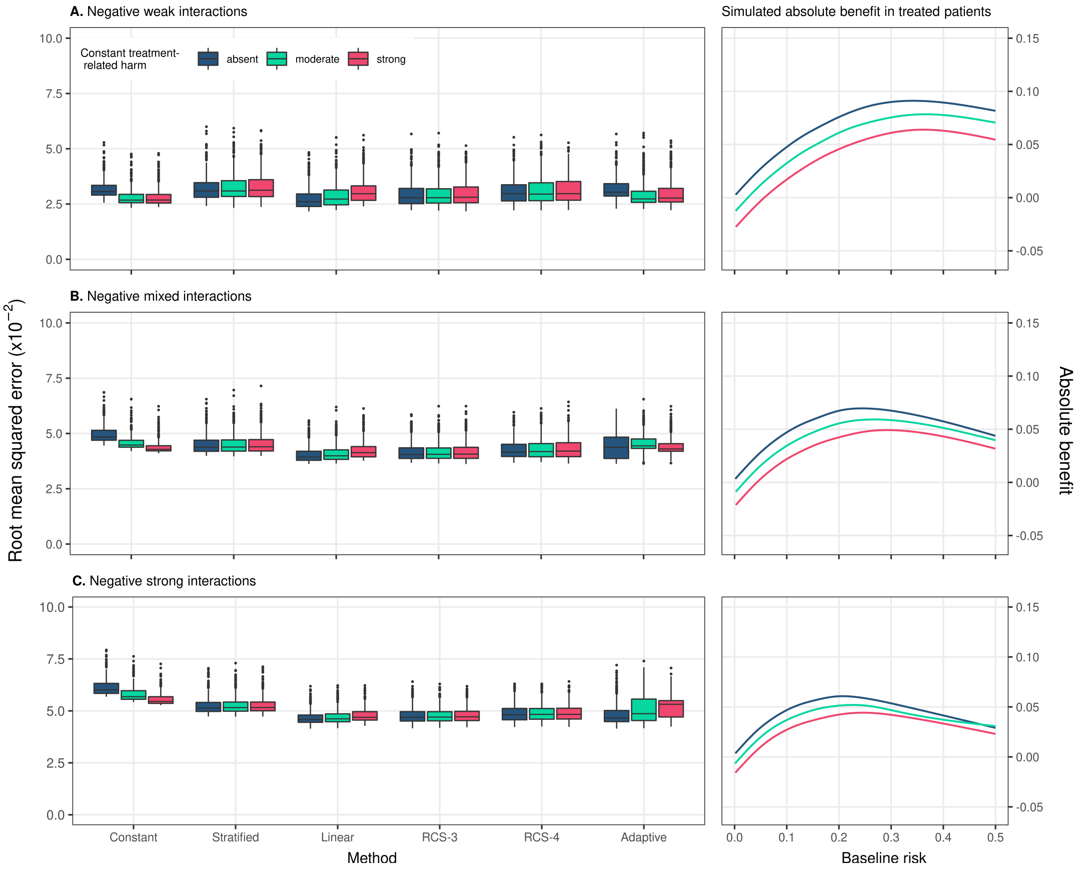

Figure S14: RMSE of the considered methods across 500 replications calculated in a simulated sample of size 500,000 where treatment-covariate interactions all favoring the control were considered.

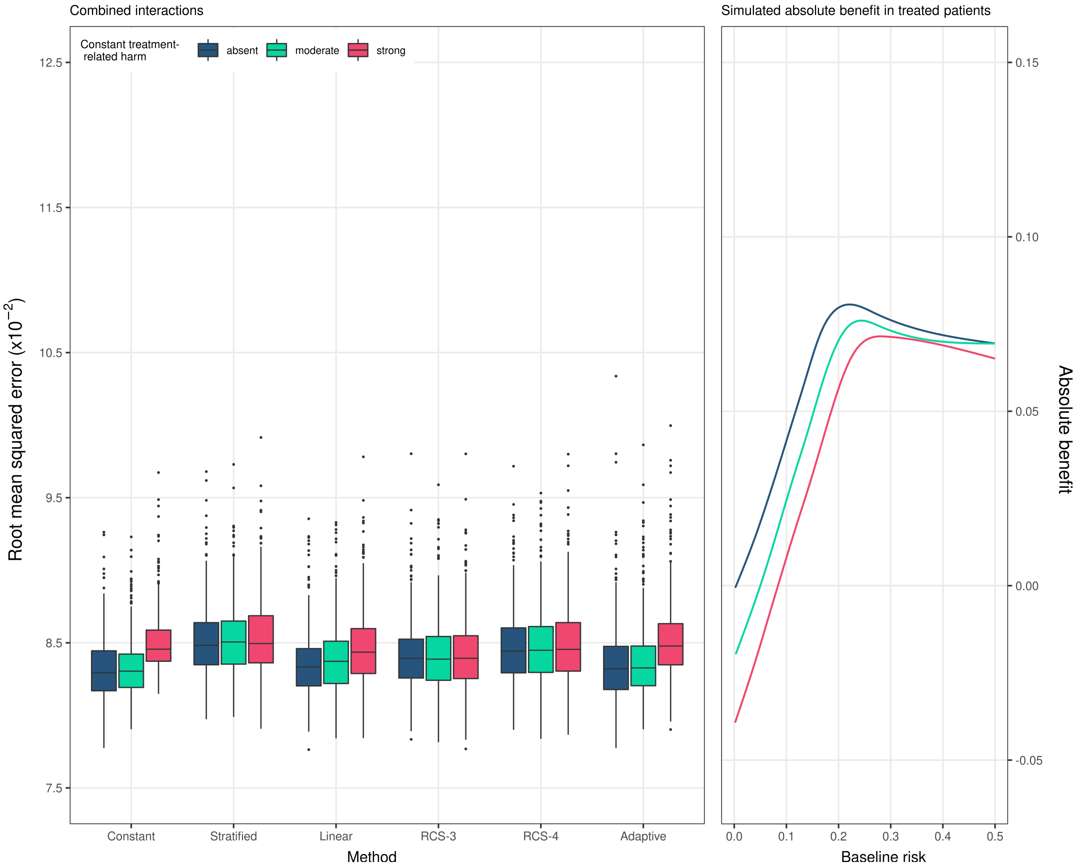

Figure S15: RMSE of the considered methods across 500 replications calculated in a simulated sample of size 500,000 where treatment-covariate interactions 2 favoring treatment and 2 favoring the control were considered.

9 Correlated covariates

We analyzed the sensitivity of our results to correlation between baseline characteristics by incuding additional simulation scenarios. We sampled covariates $W_1, \dots, W_8 \sim N(0, \Sigma)$. We generated four continuous baseline covariates $X_1 = W_1, \dots, X_4 = W_4$ and four binary covariates with 20% prevalence $X_5 = I(W_5 > z_{0.8}), X_8 = I(W_8 > z_{0.8})$, where I is the indicator function. We selected the covariance matrix Σ such that $\text{cor}(X_i, X_j) = 0.5$, for any $i \neq j$. More precisely, we set Σ as can be seen below:

$$\Sigma = \begin{pmatrix} 1 & 0.5 & 0.5 & 0.5 & 0.708 & 0.708 & 0.708 & 0.708 \\ 0.5 & 1 & 0.5 & 0.5 & 0.708 & 0.708 & 0.708 & 0.708 \\ 0.5 & 0.5 & 1 & 0.5 & 0.708 & 0.708 & 0.708 & 0.708 \\ 0.5 & 0.5 & 0.5 & 1 & 0.708 & 0.708 & 0.708 & 0.708 \\ 0.708 & 0.708 & 0.708 & 0.708 & 1 & 0.745 & 0.745 & 0.745 \\ 0.708 & 0.708 & 0.708 & 0.708 & 0.745 & 1 & 0.745 & 0.745 \\ 0.708 & 0.708 & 0.708 & 0.708 & 0.745 & 0.745 & 1 & 0.745 \\ 0.708 & 0.708 & 0.708 & 0.708 & 0.745 & 0.745 & 0.745 & 1 \end{pmatrix}$$

In order to ensure that the simulated datasets were comparable to the original main simulation scenarios, i.e. control arm event rate of 20% and true risk model c-statistic (AUC) equal to the target, we needed to adjust the coefficients of the true risk model. The exact settings of the simlation scenarios for the sensitivity analyses can be found in Table S3.

Table S3: Scenario settings of the entire simulation study.

Analysis ID					Baseline risk									True treatment effect			
Scenario	Base	N	AUC	Treatment-related harm	b0	b1	b2	b3	b4	b5	b6	b7	b8	g0	g1	g2	c
677	moderate	4,250	0.75	absent	-1.69	0.18	0.18	0.18	0.18	0.18	0.18	0.18	0.18	-0.223	1.000	0.000	0
678	moderate	4,250	0.75	moderate-positive	-1.69	0.18	0.18	0.18	0.18	0.18	0.18	0.18	0.18	-0.223	1.000	0.000	0
679	moderate	4,250	0.75	strong-positive	-1.69	0.18	0.18	0.18	0.18	0.18	0.18	0.18	0.18	-0.223	1.000	0.000	0
680	moderate	4,250	0.75	negative	-1.69	0.18	0.18	0.18	0.18	0.18	0.18	0.18	0.18	-0.223	1.000	0.000	0
681	moderate	4,250	0.75	absent	-1.69	0.18	0.18	0.18	0.18	0.18	0.18	0.18	0.18	-0.460	0.796	0.000	0
682	moderate	4,250	0.75	moderate-positive	-1.69	0.18	0.18	0.18	0.18	0.18	0.18	0.18	0.18	-0.460	0.796	0.000	0
683	moderate	4,250	0.75	strong-positive	-1.69	0.18	0.18	0.18	0.18	0.18	0.18	0.18	0.18	-0.460	0.796	0.000	0
684	moderate	4,250	0.75	negative	-1.69	0.18	0.18	0.18	0.18	0.18	0.18	0.18	0.18	-0.460	0.796	0.000	0
685	moderate	4,250	0.75	absent	-1.69	0.18	0.18	0.18	0.18	0.18	0.18	0.18	0.18	-4.420	1.000	-0.052	-5
686	moderate	4,250	0.75	moderate-positive	-1.69	0.18	0.18	0.18	0.18	0.18	0.18	0.18	0.18	-4.420	1.000	-0.052	-5
687	moderate	4,250	0.75	strong-positive	-1.69	0.18	0.18	0.18	0.18	0.18	0.18	0.18	0.18	-4.420	1.000	-0.052	-5
688	moderate	4,250	0.75	negative	-1.69	0.18	0.18	0.18	0.18	0.18	0.18	0.18	0.18	-4.420	1.000	-0.052	-5
689	moderate	4,250	0.75	absent	-1.69	0.18	0.18	0.18	0.18	0.18	0.18	0.18	0.18	0.173	1.560	0.105	0
690	moderate	4,250	0.75	moderate-positive	-1.69	0.18	0.18	0.18	0.18	0.18	0.18	0.18	0.18	0.173	1.560	0.105	0
691	moderate	4,250	0.75	strong-positive	-1.69	0.18	0.18	0.18	0.18	0.18	0.18	0.18	0.18	0.173	1.560	0.105	0
692	moderate	4,250	0.75	negative	-1.69	0.18	0.18	0.18	0.18	0.18	0.18	0.18	0.18	0.173	1.560	0.105	0
693	moderate	17,000	0.75	absent	-1.69	0.18	0.18	0.18	0.18	0.18	0.18	0.18	0.18	-0.223	1.000	0.000	0
694	moderate	17,000	0.75	moderate-positive	-1.69	0.18	0.18	0.18	0.18	0.18	0.18	0.18	0.18	-0.223	1.000	0.000	0
695	moderate	17,000	0.75	strong-positive	-1.69	0.18	0.18	0.18	0.18	0.18	0.18	0.18	0.18	-0.223	1.000	0.000	0
696	moderate	17,000	0.75	negative	-1.69	0.18	0.18	0.18	0.18	0.18	0.18	0.18	0.18	-0.223	1.000	0.000	0
697	moderate	17,000	0.75	absent	-1.69	0.18	0.18	0.18	0.18	0.18	0.18	0.18	0.18	-0.460	0.796	0.000	0
698	moderate	17,000	0.75	moderate-positive	-1.69	0.18	0.18	0.18	0.18	0.18	0.18	0.18	0.18	-0.460	0.796	0.000	0
699	moderate	17,000	0.75	strong-positive	-1.69	0.18	0.18	0.18	0.18	0.18	0.18	0.18	0.18	-0.460	0.796	0.000	0
700	moderate	17,000	0.75	negative	-1.69	0.18	0.18	0.18	0.18	0.18	0.18	0.18	0.18	-0.460	0.796	0.000	0
701	moderate	17,000	0.75	absent	-1.69	0.18	0.18	0.18	0.18	0.18	0.18	0.18	0.18	-4.420	1.000	-0.052	-5
702	moderate	17,000	0.75	moderate-positive	-1.69	0.18	0.18	0.18	0.18	0.18	0.18	0.18	0.18	-4.420	1.000	-0.052	-5
703	moderate	17,000	0.75	strong-positive	-1.69	0.18	0.18	0.18	0.18	0.18	0.18	0.18	0.18	-4.420	1.000	-0.052	-5
704	moderate	17,000	0.75	negative	-1.69	0.18	0.18	0.18	0.18	0.18	0.18	0.18	0.18	-4.420	1.000	-0.052	-5
705	moderate	17,000	0.75	absent	-1.69	0.18	0.18	0.18	0.18	0.18	0.18	0.18	0.18	0.173	1.560	0.105	0
706	moderate	17,000	0.75	moderate-positive	-1.69	0.18	0.18	0.18	0.18	0.18	0.18	0.18	0.18	0.173	1.560	0.105	0
707	moderate	17,000	0.75	strong-positive	-1.69	0.18	0.18	0.18	0.18	0.18	0.18	0.18	0.18	0.173	1.560	0.105	0
708	moderate	17,000	0.75	negative	-1.69	0.18	0.18	0.18	0.18	0.18	0.18	0.18	0.18	0.173	1.560	0.105	0
709	moderate	4,250	0.85	absent	-2.15	0.35	0.35	0.35	0.35	0.35	0.35	0.35	0.35	-0.223	1.000	0.000	0
710	moderate	4,250	0.85	moderate-positive	-2.15	0.35	0.35	0.35	0.35	0.35	0.35	0.35	0.35	-0.223	1.000	0.000	0
711	moderate	4,250	0.85	strong-positive	-2.15	0.35	0.35	0.35	0.35	0.35	0.35	0.35	0.35	-0.223	1.000	0.000	0
712	moderate	4,250	0.85	negative	-2.15	0.35	0.35	0.35	0.35	0.35	0.35	0.35	0.35	-0.223	1.000	0.000	0
713	moderate	4,250	0.85	absent	-2.15	0.35	0.35	0.35	0.35	0.35	0.35	0.35	0.35	-0.410	0.785	0.000	0
714	moderate	4,250	0.85	moderate-positive	-2.15	0.35	0.35	0.35	0.35	0.35	0.35	0.35	0.35	-0.410	0.785	0.000	0
715	moderate	4,250	0.85	strong-positive	-2.15	0.35	0.35	0.35	0.35	0.35	0.35	0.35	0.35	-0.410	0.785	0.000	0
716	moderate	4,250	0.85	negative	-2.15	0.35	0.35	0.35	0.35	0.35	0.35	0.35	0.35	-0.410	0.785	0.000	0
717	moderate	4,250	0.85	absent	-2.15	0.35	0.35	0.35	0.35	0.35	0.35	0.35	0.35	-4.130	1.000	-0.059	-5

718	moderate	4,250	0.85	moderate-positive	-2.15	0.35	0.35	0.35	0.35	0.35	0.35	0.35	0.35	-4.130	1.000	-0.059	-5
719	moderate	4,250	0.85	strong-positive	-2.15	0.35	0.35	0.35	0.35	0.35	0.35	0.35	0.35	-4.130	1.000	-0.059	-5
720	moderate	4,250	0.85	negative	-2.15	0.35	0.35	0.35	0.35	0.35	0.35	0.35	0.35	-4.130	1.000	-0.059	-5
721	moderate	4,250	0.85	absent	-2.15	0.35	0.35	0.35	0.35	0.35	0.35	0.35	0.35	-0.085	1.354	0.074	0
722	moderate	4,250	0.85	moderate-positive	-2.15	0.35	0.35	0.35	0.35	0.35	0.35	0.35	0.35	-0.085	1.354	0.074	0
723	moderate	4,250	0.85	strong-positive	-2.15	0.35	0.35	0.35	0.35	0.35	0.35	0.35	0.35	-0.085	1.354	0.074	0
724	moderate	4,250	0.85	negative	-2.15	0.35	0.35	0.35	0.35	0.35	0.35	0.35	0.35	-0.085	1.354	0.074	0

The results of the sensitivity analyses can be explored in Figures . . . We found no noticeable differences between methods for individualizing treatment benefit predictions compared to the results of the simulation scenarios where baseline covariates were assumed to be statistically independent.

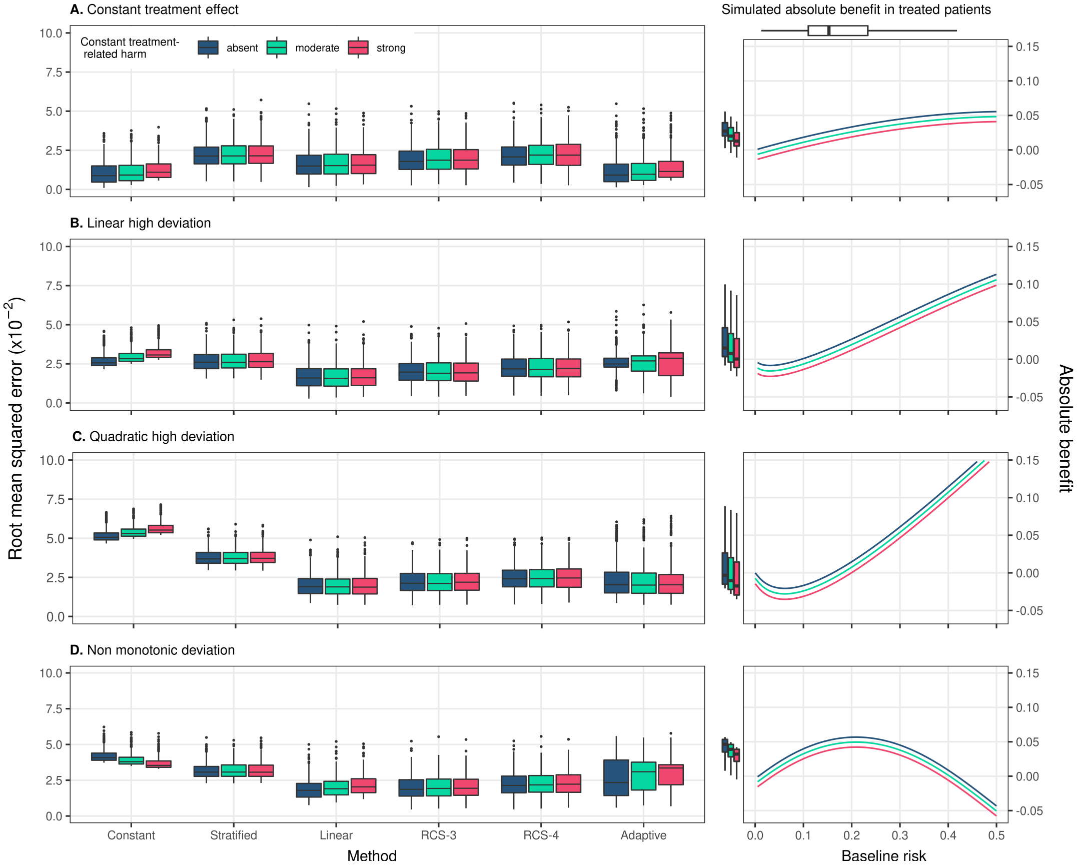

Figure S16: RMSE of the sensitivity analyses assuming correlated baseline covariates. All considered methods were derived using simulated samples of size 4,250 and true prediction c-statistic of 0.75

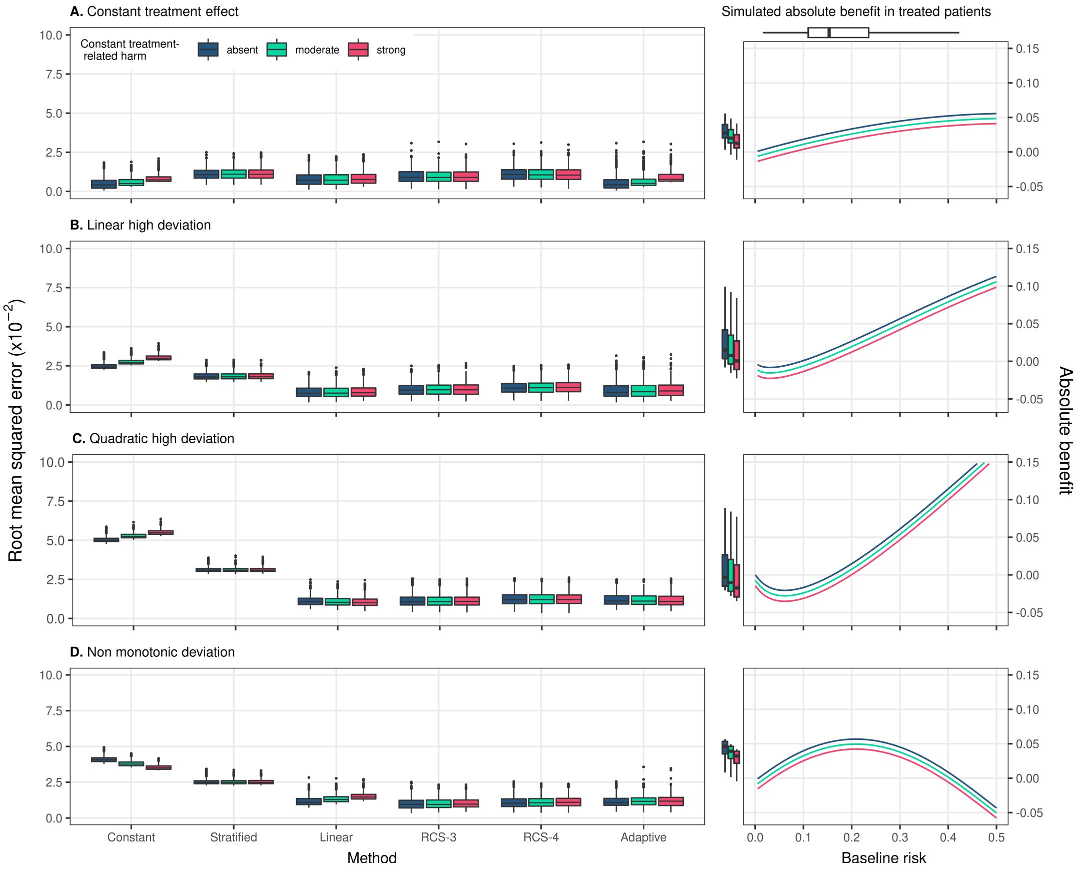

Figure S17: RMSE of the sensitivity analyses assuming correlated baseline covariates. All considered methods were derived using simulated samples of size 17,000 and true prediction c-statistic of 0.75

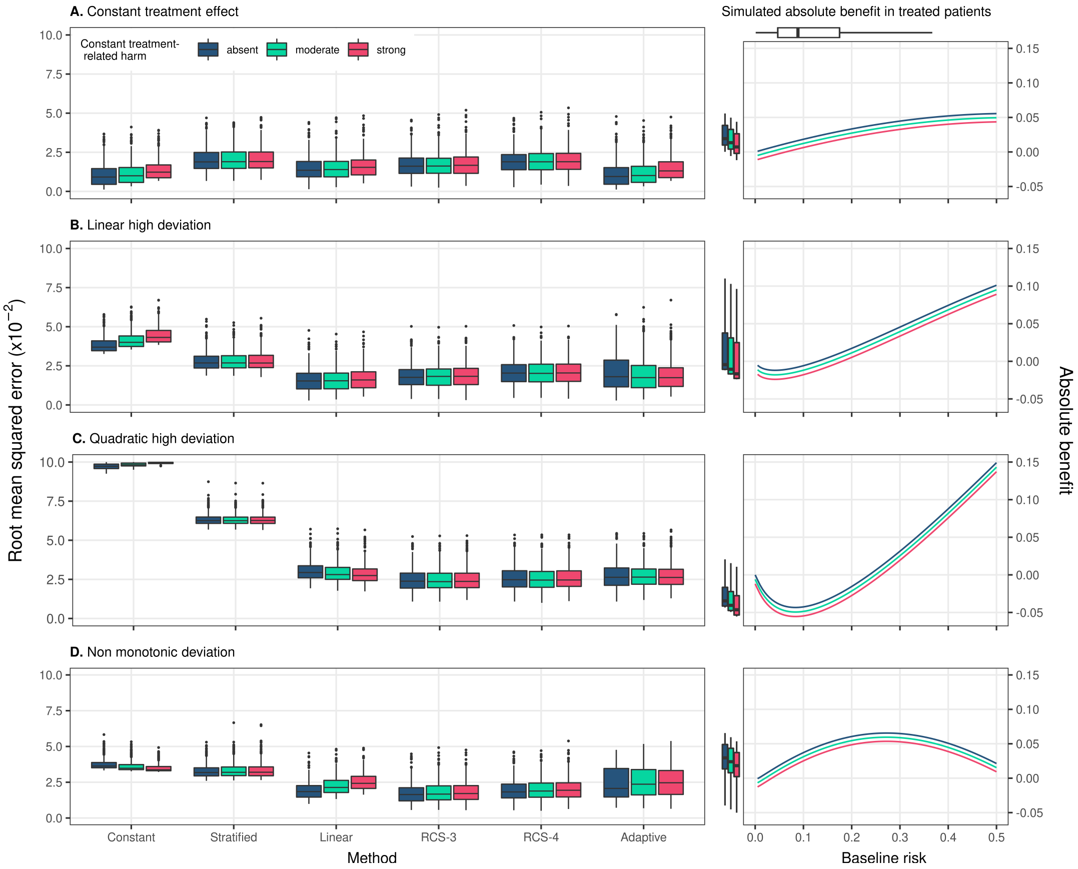

Figure S18: RMSE of the sensitivity analyses assuming correlated baseline covariates. All considered methods were derived using simulated samples of size 4,250 and true prediction c-statistic of 0.85

10 Empirical illustration

10.1 Prediction model

For predicting baseline risk of 30-day mortality we fitted a logistic regression model with age, Killip class (*Killip*), systolic blood pressure (*sysbp*), pulse rate (*pulse*), prior myocardial infarction (*pmi*), location of myocardial infarction (*miloc*) and treatment as the covariates. Baseline predictions were made setting treatment to 0.

$$P(outcome = 1|X = x) = \text{expit}(lp(x)),$$

where

$$\begin{aligned} lp(x) = &\beta_0 + \beta_1\text{age} + \beta_2 I(\text{Killip} = II) + \beta_3 I(\text{Killip} = III)+ \\ &\beta_4 I(\text{Killip} = IV) + \beta_5 min(\text{sysbp}, 120) + \beta_6\text{pulse}+ \\ &\beta_7 max(\text{pulse - 50, 0}) + \beta_8 I(\text{pmi} = yes)+ \\ &\beta_9 I(\text{miloc} = Anterior) + \beta_9 I(\text{miloc} = Other)+ \\ &\gamma \times \text{treatment} \end{aligned}$$

and $expit(x) = \frac{e^x}{1+e^x}$

Table S4: Coefficients of the prediction model for 30-day mortality, based on the data from GUSTO-I trial.

Variable	Estimate	stderror	zvalue	pvalue
Intercept	-3.020	0.797	-3.788	0.000
Age	-0.208	0.053	-3.935	0.000
Killip class = II	0.077	0.002	31.280	0.000
Killip class = III	0.614	0.059	10.423	0.000
Killip class = IV	1.161	0.121	9.566	0.000
Systolic blood pressure	1.921	0.162	11.872	0.000
Pulse rate (1)	-0.039	0.002	-20.332	0.000
Pulse rate (2)	-0.024	0.016	-1.521	0.128
Previous MI (yes)	0.043	0.016	2.675	0.007
MI location (Other)	0.447	0.056	7.964	0.000
MI location (Anterior)	0.286	0.135	2.126	0.033
Treatment	0.543	0.051	10.625	0.000

10.2 Bootstrap confidence intervals

Bootstrap confidence intervals in Figure 6 of the main manuscript were derived using the following approach:

1. Draw with replacement a sample D^* from the original dataset D of the same size as D.
2. Fit a logistic regression model m^* in D^* to predict 30-day mortality using the same covariates as the initial model m estimated in D.
3. Using the linear predictor $\hat{lp}^*$ of model m^*, fit models for constant relative treatment effect, linear interaction of the linear predictor with treatment, and interaction of treatment with a restricted cubic splines transformation of the linear predictor in D^*, as described in the *Methods* section.
4. Based on m^*, use predicted risks to stratify D^* into risk quarters and estimate absolute treatment effects within risk strata.
5. Repeat steps 1 through 4 for a total of $b = 10000$ times.
6. Derive the confidence band for each continuous method (not risk stratification) as follows:

a. Split the range $(0, 0.25]$ of baseline risk values into 499 equal-length intervals of the form $(p_0, p_1], \dots, (p_{498}, p_{499}]$.
b. For a specific point $p_k, k = 1 \dots, 499$ and each method i, use the 2.5 and 97.5 percentiles of the b absolute benefit estimates to define the confidence interval $(q_{0.025}^{i,p_k}, q_{0.975}^{i,p_k})$.

7. Derive two sets of confidence intervals for the risk stratification approach as follows:

   a. *Treatment effect:* For each risk quarter identified by the original model m developed on D, the confidence interval for the mean absolute treatment effect is derived from the 2.5 and 97.5 percentiles of the mean absolute treatment effects estimated within each risk quarter across the b bootstrap samples.
   b. *Mean predicted risk:* For each risk quarter identified by the original model m, the confidence interval for the quarter-specific mean predicted risk is derived from the 2.5 and 97.5 percentiles of the mean predicted risks estimated within each risk quarter across the b bootstrap samples.

11 References

[1] Kent DM, Nelson J, Dahabreh IJ, Rothwell PM, Altman DG, Hayward RA. Risk and treatment effect heterogeneity: Re-analysis of individual participant data from 32 large clinical trials. International Journal of Epidemiology 2016:dyw118. https://doi.org/10.1093/ije/dyw118.

[2] Harrell FE. Regression modeling strategies. vol. 330. Springer; 2017.

[3] Austin PC, Steyerberg EW. The integrated calibration index (ICI) and related metrics for quantifying the calibration of logistic regression models. Statistics in Medicine 2019;38:4051–65. https://doi.org/10.1002/sim.8281.
